# An On‐Demand Neuromorphic Vision System Enabled by a Multi‐Paradigm Neuromorphic Device and Hierarchical Reconfigurability Designed from Device to System Level

**DOI:** 10.1002/advs.202520448

**Published:** 2026-02-11

**Authors:** Biyi Jiang, Jiayi Xu, Liang Ran, Xinhe Feng, Khalil Harrabi, Yida Li, Longyang Lin, Feichi Zhou

**Affiliations:** ^1^ School of Microelectronics Southern University of Science and Technology Shenzhen China; ^2^ Beijing Pixelcore Technology Co., Ltd Beijing China; ^3^ Department of Physics, College of Engineering and Physics King Fahd University of Petroleum and Minerals Dhahran Saudi Arabia; ^4^ Interdisciplinary Research Center for Advanced Quantum Computing King Fahd University of Petroleum and Minerals (KFUPM) Dhahran Saudi Arabia

**Keywords:** general purpose, neuromorphic device, vision system

## Abstract

There are two general approaches in guiding intelligent vision system development: one emphasizes ultra‐flexibility (reconfigurability) for adapting to various scenarios, and the other emphasizes ultrahigh power efficiency tailored to specific applications. The pinnacle design is geared toward the biological vision system with concurrent high levels of on‐demand intelligence, efficiency, and flexibility. However, current state‐of‐the‐art intelligent vision systems are far behind, relying on heterogeneously integrated and limited‐function single devices, alongside rigid sensing/computing architecture, thus preventing flexibility for low area and power efficiency toward dynamic and unpredictable scenarios. This work bridges the neuromorphic gap with an on‐demand ultra‐reconfigurable vision system, demonstrating true reconfigurability across device, cell, array and system levels. This is enabled by a multi‐paradigm device array capable of seamless switching between spiking, non‐spiking, neuromorphic imaging (NI), and artificial intelligence (AI) computing modes, as well as a reconfigurable circuit and architecture design. The system is capable of on‐demand allocating resources between NI and AI functionalities for high‐quality smart imaging and high‐accuracy recognition tasks, and transitioning between spiking and non‐spiking modes for frameless dynamic and frame‐based static scenarios. Superior power efficiencies of up to 52.6 TOPS/W for NI‐centric computing and 75.5 TOPS/W for NI/AI hybrid computing are achieved, which are up to two orders of magnitude larger than the state‐of‐the‐art intelligent vision system.

## Graphical abstract

An on‐demand ultra‐reconfigurable intelligent vision system with hierarchical reconfigurability from device to system levels is demonstrated. Through co‐design of a multi‐paradigm device, reconfigurable circuits, and adaptive system architecture/algorithms, the system enables seamless switching among spiking, non‐spiking, neuromorphic imaging (NI), and artificial intelligence (AI) modes, achieving up to two orders of magnitude higher energy efficiency (75.5 TOPS/W) than state‐of‐the‐art intelligent vision systems.

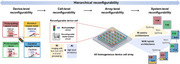

## 1 Introduction

An ideal intelligent vision system must simultaneously fulfil two critical design objectives: high flexibility (reconfigurability) to adapt to diverse scenarios, and high power/area efficiency for specific applications, in order to achieve the next evolutionary breakthrough in intelligent vision technology. Current implementations, however, face a fundamental compromise between these competing goals, limiting their overall performance. This challenge becomes particularly prominent when such systems are deployed for edge computing due to constraints in limited hardware resources and stringent power budgets, but is still expected to handle the multi‐dynamics and unpredictability of vision events in unconstrained environments.

Conventional intelligent vision systems are constrained to specialized processing paradigms, such as employing a spiking approach for frame‐free motion scenarios [1, 2] or a non‐spiking approach for frame‐based static scenarios [3], leading to compromised adaptability and computational inefficiency in dynamic and unpredictable real‐world environments. While there has been some progress in recent state‐of‐the‐art reconfigurable CMOS intelligent vision systems in achieving spiking/non‐spiking reconfigurability and algorithm deployment (at chip and block levels) in front‐end imaging hardware or back‐end artificial intelligence vision processors for versatile scenario adaptation [4, 5, 6, 7, 8], their overall performances are still lacklustre. This is because they fall short from a fundamental device and architecture point of view. Specifically, at the device cell level, they mainly rely on heterogeneously integrated and single‐function devices, lacking multi‐paradigm reconfigurability. Moreover, at an architectural level, the rigid sensing‐computing architecture prevents the system's flexibility and efficiencies across a wide variety of scenarios with specific demands (e.g., neuromorphic imaging‐oriented scenarios with high‐quality smart imaging requirements and AI computing‐oriented scenarios with high‐accuracy AI computing requirements).

Hence, guidance is often drawn from biological vision systems, which are highly capable of adapting to ever‐changing scenarios and performing reliable visual sensing and processing with extremely high on‐demand intelligence, flexibility, and efficiency [9]. This capability stems from the rich dynamics of visual neurons in the retina and cortex, as well as inherent structural homogeneity and extensive connectivity. As a result, the human vision system operates in a task‐dependent manner through “cognitive” architectures [9, 10, 11, 12, 13], enabling ultra‐flexible reconfiguration of neural pathways to maximize efficiency in response to real‐time computational demands. Motivated by these biological principles, a variety of neuromorphic devices with multiple computing modes have been actively explored [14, 15, 16], including three‐terminal, tripartite‐synapse‐inspired phototransistors [17]; electrochemical transistor‐based synapses [18]; multi‐mode optoelectronic memristors [19]; and one‐phototransistor‐one‐memristor cell [20]. Despite incorporating advanced biological visual functionalities, state‐of‐the‐art neuromorphic vision systems based on emerging reconfigurable devices still face fundamental limitations due to the restricted device‐level reconfigurability in seamlessly supporting both retina‐like neuromorphic imaging (NI) and cortex‐like artificial intelligence (AI) computing across spiking and non‐spiking operational modes. More critically, inadequate circuit, architecture, and algorithm designs that fail to build hierarchical reconfigurability across all levels, results in a “device‐flexible‐but‐system‐rigid” dilemma, compromising overall system power and area efficiency [19, 20, 21, 22, 23, 24, 25, 26, 27].

**FIGURE 1:**
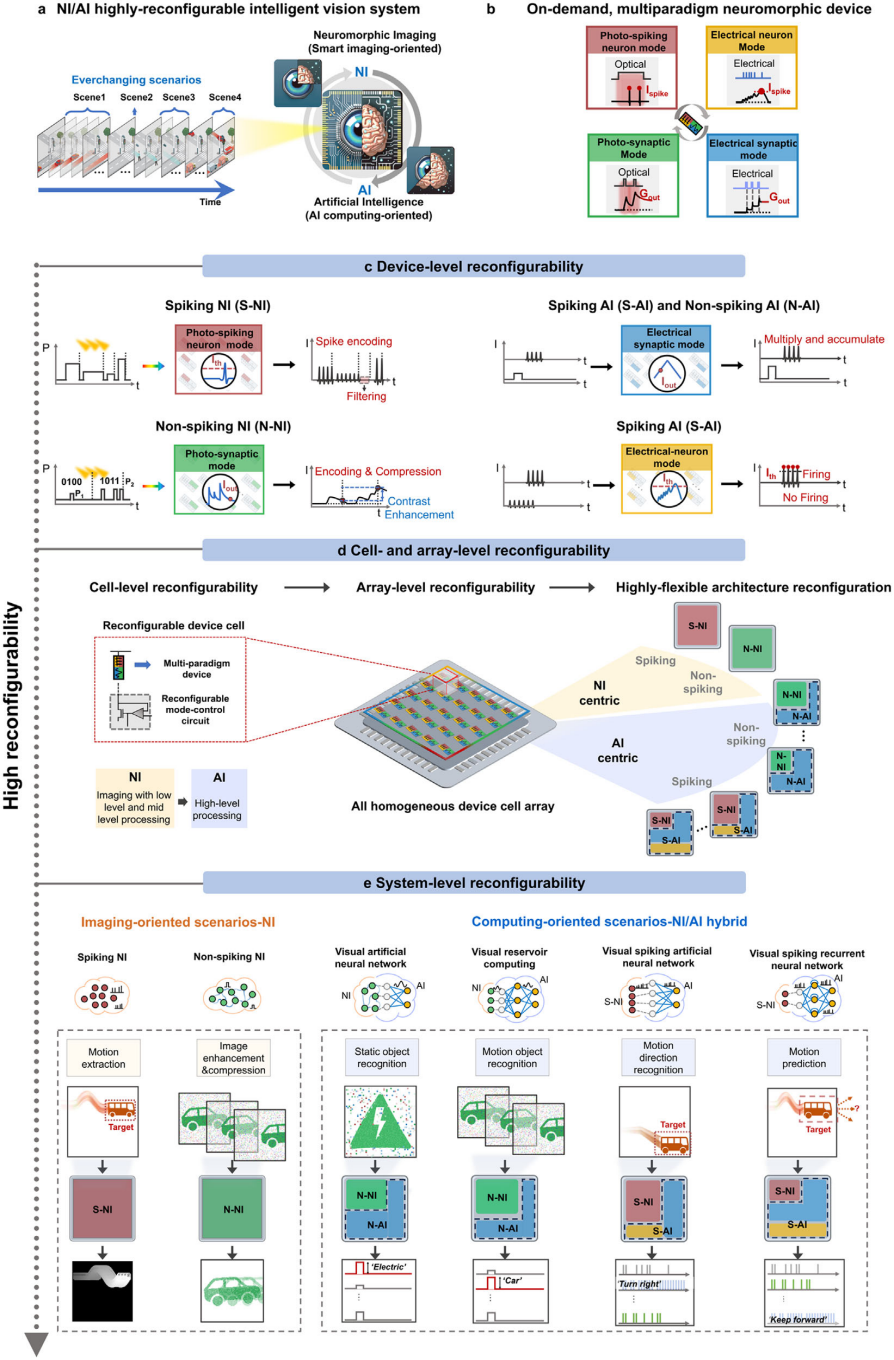
On‐demand, highly reconfigurable intelligent vision system with multi‐paradigm device and hierarchical reconfigurability across device to system levels. (a) A highly flexible NI/AI reconfigurable intelligent vision system enabled by (b) a multi‐paradigm neuromorphic vision device with co‐existence of four neuromorphic behaviors, mimicking the elemental functionalities in the retina (NI) and cortex (AI). (c) Device‐level reconfigurability: device‐algorithm co‐design of achieving four visual processing modes, including: photo‐spiking neuron mode for spiking NI with in‐sensor spike encoding and filtering capabilities, photo‐synaptic mode for non‐spiking NI with in‐sensor data compression and enhancement capabilities, electrical synaptic and electrical neuron mode for spiking and non‐spiking AI with in‐memory multiplication capability and threshold comparison capability. (d) Cell‐ and array‐level reconfigurability: an all‐homogeneous cell array with cell‐ and array‐level reconfigurability is demonstrated, enabled by a reconfigurable mode‐control circuit design. This array support flexible architecture reconfigurations with different NI/AI sizes in both spiking and non‐spiking modes, by flexibly switching the four cell modes. (e) System‐level reconfigurability: an on‐demand intelligent vision system with across device‐to‐system levels reconfigurability supports a broad spectrum of intelligent vision applications, spanning from spatiotemporally frame‐free motion to spatially frame‐based static scenarios, and from imaging‐oriented to AI computing‐oriented scenarios. Demonstrated configurations include two NI‐centric configurations (spiking NI and non‐spiking NI), and four hybrid NI/AI configurations (visual spiking recurrent neural network‐VSRNN, visual spiking artificial neural network‐VSANN, visual artificial neural network‐VANN, and visual reservoir computing‐VRC, covering versatile scenarios including motion extraction, image enhancement, image compression, object recognition, motion recognition, and motion direction prediction.

Here, we address these challenges by presenting an on‐demand and highly reconfigurable intelligent vision system (Figure 1a,b), enabled by the employment of a multi‐paradigm device, and the synergistic co‐design of reconfigurable circuits and an adaptable dataflow architecture, which enables full spiking/non‐spiking/NI/AI reconfigurability across device‐to‐system levels. We achieve four sensory computing modes by co‐designing device algorithms with the multi‐paradigm device behaviors reported in our recent work [28] (Figure 1b), including photo‐spiking neuron mode for spiking NI, photo‐synaptic mode for non‐spiking NI, and electrical synaptic and electrical neuron modes for spiking and non‐spiking AI computing (Figure 1c). With a reconfigurable mode‐control circuit design, an all‐homogeneous cell array with cell‐ and array‐level reconfigurability is demonstrated, which can support flexible architecture reconfigurations with different NI/AI sizes in both spiking and non‐spiking modes, by flexibly switching the four cell modes (Figure 1d). An on‐demand neuromorphic vision system with spiking/non‐spiking/NI/AI reconfigurability across device to system levels (Figure 1e) is then constructed. To address dynamic requirements across imaging‐oriented to computing‐oriented scenarios, and frame‐free motion to frame‐based static scenarios, we demonstrate six on‐demand configurations using our system within a unified platform. These include two imaging‐oriented NI configurations: (1) A spiking NI configuration and (2) A non‐spiking NI configuration, both supporting high‐resolution motion and static imaging with preprocessing, as well as four computing‐oriented hybrid NI/AI configurations are implemented: (3) Visual spiking artificial neural network (V‐SANN) and (4) Visual spiking recurrent neural network (V‐SRNN) for motion direction recognition and prediction, as well as (5) Visual non‐spiking artificial neural network (V‐ANN) and (6) Visual reservoir computing (V‐RC) for static and motion object recognition. The flexibility in reconfiguring to different NI/AI sizes in our system allows maximal resource allocation within a unified array, thus enabling high accuracy, power and area efficiency as compared to NI/AI non‐reconfigurable vision system (Figure S1).

## 2 Results and Discussion

### 2.1 Device‐Level Reconfigurability

We present the device‐level reconfigurability and algorithm in achieving four sensory computing modes by utilising a multi‐paradigm device with a stack structure of Pd/CuO_x_/ITO reported earlier [28] but with device size further scaled down in this work (Figure S2). Similarly, four different behaviors can be accessed at different voltage regimes, including photo‐spiking neuron (PN) mode at V_bias_ = 0 V, photo‐synaptic (PS) mode at −0.1 V < V_bias_ < −0.04 V, electrical synaptic (ES) mode at 0.1 V < V_bias_ < 0.5 V, and electrical neuron (EN) mode at V_bias_ > 1.3 V, as shown in the basic *I*–*V* characteristics (Figure S3). The details of the device's working mechanisms are provided in Note S1 and Figure S4, with a more comprehensive explanation available in our previous publication [28]. The device's behaviors are then modelled together with the co‐designed device algorithms to achieve four sensory computing modes (Table 1), for use in the subsequent system‐level algorithms for dedicated functions. All sensory computing modes are operated with low energy consumption (Table S1 and S2 and Note S2), and the benchmarking with other neuromorphic devices is provided in Table S3 and S4 [19, 20, 24, 29, 30, 31, 32, 33, 34, 35, 36]. In addition, the device exhibits excellent time stability, suggesting no degradation for all four modes during four months (Figure S5). Descriptions of each sensory computing mode are as follows:

**TABLE 1:** Summary of the basic behaviors of the four sensory computing modes, along with the corresponding implementable algorithms and applications.

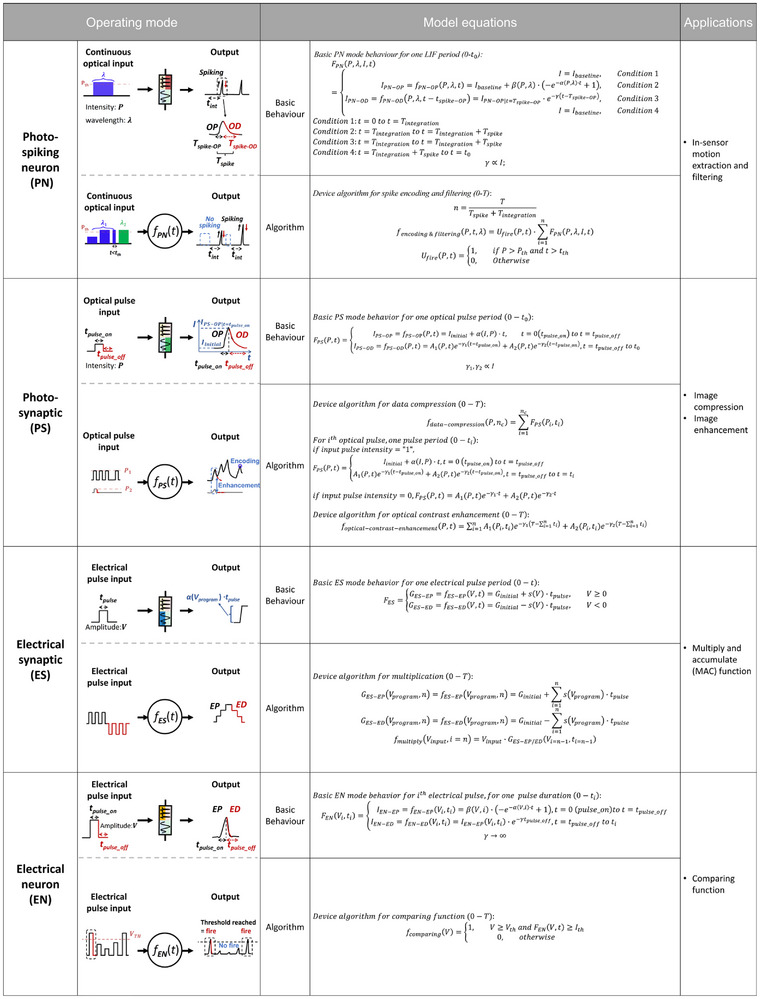

#### 2.1.1 Photo‐Spiking Neuron (PN) Mode

**FIGURE 2:**
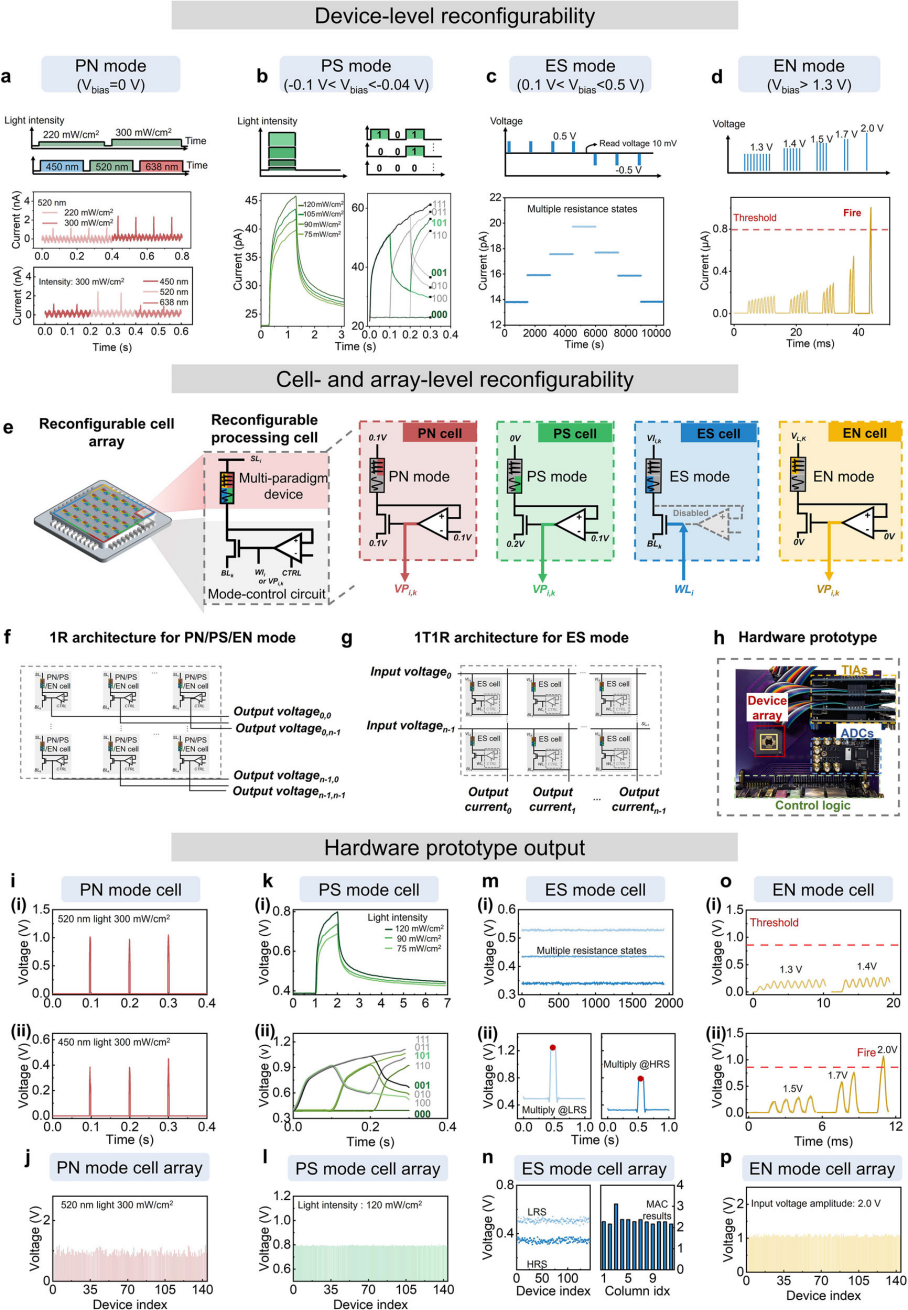
Device‐, cell‐, and array‐level reconfigurability. Device‐level reconfigurability: (a) PN mode: photoresponsive LIF spiking characteristics of the device under light intensities of 220 and 300 mW/cm^2^ (Upper panel). Photoresponsive LIF spiking characteristics of the device in PN mode under 450, 520, and 638 nm light illumination with intensity of 300 mW/cm^2^ (Lower panel). (b) PS mode: Device response under 520 nm, 1 s light pulse with varying optical pulse intensities ranging from 75 to 120 mW/cm^2^ (Left panel). Device response under eight different 3‐bit light pulse stimuli pattern denoted by “000” to “111” for data compression (pulse intensities: 0 mW/cm^2^ for “0” and 1200 mW/cm^2^ for “1”, pulse width = 100 ms, V_read_ = −40 mV, 520 nm wavelength) (Right panel). (c) ES mode: LTP and LTD behaviors under 0.5 and −0.5 V electrical pulses with pulse width of 50 ms, respectively. (d) EN mode: LIF characteristics under electrical pulse stimuli with varying amplitudes and pulse numbers (from 1.3 V for 10 pulses to 2.0 V for 1 pulse), with a pulse width of 0.5 ms and a pulse interval of 0.5 ms. Cell‐ and array‐level reconfigurability: (e) Reconfigurable cell array architecture. Each cell integrates a multi‐paradigm device and mode‐control circuit. (f) 1R‐cell array architecture for PN/PS/EN modes. (g) 1T1R cross‐point cell array architecture for ES mode. (h) Photograph of the board‐level reconfigurable system prototype. (i) PN mode cell output under (i) 520 nm and (ii) 450 nm illumination with light intensity of 300 mW/cm^2^. (j) Spiking results of the 12 ×12 cell array reconfigured to PN mode under 520 nm illumination with light intensity of 300 mW/cm^2^. (k) PS mode cell output under (i) single 520 nm, 1 s light pulse with varying illumination intensities ranging from 75 to 120 mW/cm^2^, and (ii) eight different input optical temporal patterns (“000”—“111”). (l) Photoresponse of the 12 × 12 array reconfigured to PS mode under 520 nm, 1 s light pulse with intensity of 120 mW/cm^2^. (m) ES mode cell output showing i. different resistance states under a constant read voltage of 0.01 V and ii. Multiplication results at HRS and LRS when a 0.015 V voltage pulse is applied. (n) Readout results at HRS and LRS, and MAC results of the 12 × 12 ES mode cell array. (o) EN mode cell response under varying pulse amplitudes and numbers (i) 1.3 V (10 pulses), 1.4 V (7 pulses), and (ii) 1.5 V (4 pulses), 1.7 V (2 pulses), 2.0 V (1 pulse), with pulse width and interval of 0.5 ms. (p) Firing results of the 12 × 12 EN mode cell array under a 2 V, 0.5 ms electrical pulse.

At a voltage bias of 0 V, the device operates in PN mode with broadband photoresponsive leaky integrate and fire (LIF) spiking characteristics that can directly convert and encode the continuous lights into current spikes, as shown in Figure 2a. An increase in light intensity from 220 to 300 mW/cm^2^ results in a larger spike amplitude (Figure 2a upper panel). Figure 2a (lower panel) shows the photoresponsive LIF spiking under different wavelengths (450, 520, and 638 nm) under the light intensity of 300 mW/cm^2^, exhibiting wavelength‐dependent spiking amplitudes. The PN mode also shows light intensity‐ and illumination duration‐based threshold spiking features, where the spiking occurs only when the intensity and illumination duration exceeds their thresholds. More systematic studies of the spiking behaviors under different wavelengths and light intensities are shown in Figure S6. The basic behavior of the device for one LIF spiking period and device algorithm describing the in‐sensor spike encoding and filtering capabilities are described by the following functions:

*Basic PN Mode Behavior for one LIF Period (0‐t*_0_)

(1) $$F_{\mathrm{PN}}\left(P,\lambda ,I,t\right)=\left\{\begin{array}{c} I=I_{\mathrm{base}\mathrm{line}},\;\mathrm{Cond}\mathrm{ition}\;1\\ I_{\mathrm{PN}-\mathrm{OP}}=f_{\mathrm{PN}-\mathrm{OP}}\left(P,\lambda ,\;t\right)=I_{\mathrm{base}\mathrm{line}}+\beta \left(P,\;\lambda \right)\cdot \left(-e^{-\alpha \left(P,\;\lambda \right)\cdot t}+1\right),\;\mathrm{Cond}\mathrm{ition}\;2\\ I_{\mathrm{PN}-\mathrm{OD}}=f_{\mathrm{PN}-\mathrm{OD}}\left(P,\lambda ,\;t-t_{\mathrm{spike}-\mathrm{OP}}\right)=I_{\mathrm{PN}-\mathrm{OP}\;|\;t=T_{\mathrm{spike}-\mathrm{OP}}}\cdot e^{-\gamma \left(t-T_{\mathrm{spike}-\mathrm{OP}}\right)},\;\mathrm{Cond}\mathrm{ition}\;3\\ I=I_{\mathrm{base}\mathrm{line}},\;\mathrm{Cond}\mathrm{ition}\;4 \end{array}\right.$$

$$Condition\;1:t=0\;to\;t=T_{integration}$$

$$Condition\;2:t=T_{integration}\;to\;t=T_{integration}+T_{spike}$$

$$Condition\;3:t=T_{integration}\;to\;t=T_{integration}+T_{spike}$$

$$Condition\;4:t=T_{integration}+T_{spike}\;to\;t=t_{0}$$

γ∝I;

where *spike‐OP* and *spike‐OD* are the potentiation section and depression section in one spike respectively, *P* is the applied light power, *λ* is the light wavelength, *I_baseline_* is baseline current, *T_spike‐OP_* is the time required for the potentiation section, *T_spike‐OD_* is the time required for the depression section, *T_integration_* is the time required before the firing occurs, *T_spike_* is the time duration of each spike, *β* is the spike amplitude, *α* is the rise factor, and *γ* is the decay factor. Building upon the basic behavior of the PN mode, the device algorithm is designed correspondingly to perform spike encoding and filtering of the input optical data as follows.

*Device Algorithm for Spike Encoding And Filtering (0‐T)*

$$n=\frac{T}{T_{spike}+T_{integration}}$$

(2) $$f_{\mathrm{encoding}\&\mathrm{filtering}}\left(P,t,\lambda \right)=U_{\mathrm{fire}}\left(P,t\right)\cdot \sum_{i=1}^{n}F_{\mathrm{PN}}\left(P,\lambda ,I,t\right)$$

(3) $$U_{\mathrm{fire}}\left(P,t\right)=\left\{\begin{array}{c} 1,\;\mathrm{if}\;P>P_{\mathrm{th}}\;\mathrm{and}\;t>t_{\mathrm{th}}\\ 0,\;\mathrm{Othe}\mathrm{rwise} \end{array}\right.$$

where *n* is the number of spikes within period T, *U_fire_* (*P*,*t*) is the firing condition with respect to the light intensity and illumination time, *P_th_* is the threshold light intensity for firing, *t_th_* is the threshold integration time for firing. Based on the spike encoding and filtering device algorithms designed above, the device can encode the light information into spike amplitude and filter the light signal with below‐threshold light intensity and integration time.

#### 2.1.2 Photo‐Synaptic (PS) Mode

The device operates in PS mode within a voltage range of −0.1 to −0.04 V. In this mode, when subjected to light pulse sequence stimulation, the device current initially undergoes continuous potentiation (optical potentiation‐OP), followed by nonlinear relaxation (optical depression‐OD) after stimulus removal, as shown in Figure 2b and Table 1. The PS mode exhibits nonlinear synaptic plasticity‐dependent on optical pulse intensity, pulse number, and pulse interval (Figures S7–S9). These properties enable in‐sensor optical data enhancement (Figure 2b (left panel)) and compression (Figure 2b (right panel)). Figure 2b (left panel) shows the current responses under varying light intensities (75–120 mW/cm^2^, pulse width: 1 s, pulse number: 1), suggesting that higher intensities induce larger current amplitudes with slow decay rates, while lower intensities produce rapidly decaying currents. This temporal amplification of output differences enhances contrast between distinct light intensities. Figure 2b (right panel) illustrates data compression achieved through the pulse temporal pattern‐dependent nonlinear synaptic plasticity. This enables encoding of 3‐bit light pulse sequences (“000”‐ “111”, where “1” represents a 100 ms light pulse at 1200 mW/cm^2^ and “0” denotes no pulse) into eight distinct 1‐bit current magnitudes, achieving data compression with compression ratio of 3. The basic behavior of the device in the PS mode within one optical pulse period and device algorithms describing data enhancement and compression functions are described by the following functions (indicated in Table 1):

*Basic* *PS* *Mode* *Behavior* *for* *One* *Optical* *Pulse* *Period* (0 − *t*_0_)

(4) $$F_{\mathrm{PS}}\left(P,t\right)=\left\{\begin{array}{c} I_{\mathrm{PS}-\mathrm{OP}}=f_{\mathrm{PS}-\mathrm{OP}}\left(P,t\right)=I_{\mathrm{init}\mathrm{ial}}+\alpha \left(I,P\right)\cdot t,\\ \;t=0\left(t_{\mathrm{pulse}\_\mathrm{on}}\right)\mathrm{to}\;t=t_{\mathrm{pulse}\_\mathrm{off}}\\ I_{\mathrm{PS}-\mathrm{OD}}=f_{\mathrm{PS}-\mathrm{OD}}\left(P,t\right)=A_{1}\left(P,t\right)e^{-\gamma_{1}\left(t-t_{\mathrm{pulse}\_\mathrm{on}}\right)}\\ \;+A_{2}\left(P,t\right)e^{-\gamma_{2}\left(t-t_{\mathrm{pulse}\_\mathrm{on}}\right)},\;t=t_{\mathrm{pulse}\_\mathrm{off}}\;\mathrm{to}\;t_{0} \end{array}\right.$$

where *OP* is the potentiation process, *OD* is the depression process, *I_initial_* is the instantaneous current before the start of the process, *t_pulse_*___ *_on_* is the time where the light is on, *t_pulse_off_* is the time where the light is off, *α* is the rise factor, *γ_1_* and *γ_2_* are the decay factors corresponding to the two exponential decay processes, and *A_1_* and *A_2_* are the scale factors for the respective exponential decay components.

*Device* *Algorithm* *for* *Optical* *Contrast* *Enhancement* (0 − *T*)

(5) $$\begin{array}{ccc} f_{\mathrm{opti}\mathrm{cal}-\mathrm{cont}\mathrm{rast}-\mathrm{enha}\mathrm{ncem}\mathrm{ent}}\left(P,t\right) & = & \sum_{i=1}^{n}A_{1}\left(P_{i},t_{i}\right)e^{-\gamma_{1}\left(T-\sum_{i=1}^{n}t_{i}\right)}\\ & & +A_{2}\left(P_{i},t_{i}\right)e^{-\gamma_{2}\left(T-\sum_{i=1}^{n}t_{i}\right)} \end{array}$$

*Device* *Algorithm* *for* *Data* *Compression* (0 − *T*)

(6) $$f_{\mathrm{data}-\mathrm{comp}\mathrm{ress}\mathrm{ion}}\left(P,n_{c}\right)=\sum_{i=1}^{n_{c}}F_{\mathrm{PS}}\left(P_{i},t_{i}\right)\;$$

*For* *i^th^* *optical* *pulse*, *one* *pulse* *period* (0 − *t_i_*):

 *if input pulse intensity* = "1",

(7) $$F_{\mathrm{PS}}\left(P,t\right)=\left\{\begin{array}{c} I_{\mathrm{init}\mathrm{ial}}+\alpha \left(I,P\right)\cdot t,\;t=0\;\left(t_{\mathrm{pulse}\_\mathrm{on}}\right)\;\mathrm{to}\;t=t_{\mathrm{pulse}\_\mathrm{off}}\\ A_{1}\left(P,t\right)e^{-\gamma_{1}\left(t-t_{\mathrm{pulse}\_\mathrm{on}}\right)}+A_{2}\left(P,t\right)e^{-\gamma_{2}\left(t-t_{\mathrm{pulse}\_\mathrm{on}}\right)},\\ \;t=t_{\mathrm{pulse}\_\mathrm{off}}\;\mathrm{to}\;t=t_{i} \end{array}\right.\;$$

*if input pulse intensity* = 0

(8) $$F_{PS}\;\left(P,t\right)=A_{1}\left(P,t\right)e^{-\gamma_{1}\cdot t}+A_{2}\left(P,t\right)e^{-\gamma_{2}\cdot t}$$

where *n* is the number of training cycles, *n_c_* is the compression ratio, and the rest of the variables are as described above.

#### 2.1.3 Electrical Synaptic (ES) Mode

The device functions as an ES mode with a low programming voltage of 0.1 to 0.5 V, demonstrating analogue and non‐volatile electrical resistive switching behaviors. The basic behavior of the device in this mode results in long‐term potentiation (LTP) and long‐term depression (LTD) characteristics under application of constant positive (0.5 V, 50 ms) and negative (−0.5 V, 50 ms) voltage pulses, respectively, as illustrated in Figure 2c. Multiple analog resistance states remain stable and reliably maintainable. Figure S10 further shows the cyclic LTP/LTD behaviors with excellent linearity. The basic behavior for one electrical pulse period and the device algorithm describing the multiplication operation are described by the following functions (indicated in Table 1):

*Basic* *ES* *Mode* *Behavior* *for* *One* *Electrical* *Pulse* *Period* (0 − *t*)

(9) $$F_{ES}=\left\{\begin{array}{c} G_{ES-EP}=f_{ES-EP}\left(V,t\right)=G_{initial}+s\left(V\right)\cdot t_{pulse},\;V\geq 0\\ G_{ES-ED}=f_{ES-ED}\left(V,t\right)=G_{initial}-s\left(V\right)\cdot t_{pulse},\;V<0 \end{array}\right.$$

where *G* is the conductance of the device, *EP* is the potentiation process, *ED* is the depression process, *G_initial_* is the initial conductance of the device, *t_pulse_* is the time duration when the electrical pulse is on, and *s* is the scaling factor for the increase in conductance.

*Device* *Algorithm* *for* *Multiplication* (0 − *T*)

(10) $$\begin{array}{ccc} G_{\mathrm{ES}-\mathrm{EP}}\left(V_{\mathrm{prog}\mathrm{ram}},n\right) & = & f_{\mathrm{ES}-\mathrm{EP}}\left(V_{\mathrm{prog}\mathrm{ram}},n\right)\\ & = & G_{\mathrm{init}\mathrm{ial}}+\sum_{i=1}^{n}s\left(V_{\mathrm{prog}\mathrm{ram}}\right)\cdot t_{\mathrm{pulse}} \end{array}$$

(11) $$\begin{array}{ccc} G_{\mathrm{ES}-\mathrm{ED}}\left(V_{\mathrm{prog}\mathrm{ram}},n\right) & = & f_{\mathrm{ES}-\mathrm{ED}}\left(V_{\mathrm{prog}\mathrm{ram}},n\right)\\ & = & G_{\mathrm{init}\mathrm{ial}}\;-\sum_{i=1}^{n}s\left(V_{\mathrm{prog}\mathrm{ram}}\right)\cdot t_{\mathrm{pulse}} \end{array}$$

(12) $$f_{\mathrm{mult}\mathrm{iply}}\left(V_{\mathrm{input}},\;i=n\right)=V_{\mathrm{input}}\cdot G_{\mathrm{ES}-\mathrm{EP}/\mathrm{ED}}\left(V_{i=n-1},t_{i=n-1}\right)$$

where *V_program_* is the programming voltage, *n* is the number of pulses applied to the device, and the rest of the variables are as described above. The device‐level multiplication algorithm ultimately supports multiply‐and‐accumulate (MAC) operations in cross‐point cell array configurations for practical applications (Figure S11).

#### 2.1.4 Electrical Neuron (EN) Mode

The device operates in EN mode at a larger voltage range of >1.3 V, exhibiting LIF behavior under continuous electrical pulse stimuli. Figure 2d presents the current responses to electrical pulses with varying amplitudes and numbers: 1.3 V (10 pulses), 1.4 V (7 pulses), 1.5 V (4 pulses), 1.7 V (2 pulses), and 2.0 V (1 pulse), using 0.5 ms pulse width/interval. The firing occurs only when the pulse amplitude exceeds the threshold levels (V ≥ 2.0 V). Figure S12 reveals frequency‐dependent firing characteristics under constant amplitude, successful firing requires minimum 2.0 ms pulse widths. The firing only initiates when input stimulus strength surpasses critical thresholds. The pulse amplitude‐, pulse frequency‐, and pulse number‐dependent LIF can be used for mimicking neurons for selecting above‐threshold signals and facilitating the decision‐making process in spiking‐based neural networks. The basic EN mode behavior for one pulse duration and a mathematical comparison function model accounting for pulse amplitude thresholds can be described by the following functions:

*Basic* *EN* *Mode* *Behavior* *for* *i^th^* *Electrical* *Pulse*, *for* *One* *Pulse* *Duration* (0 − *t_i_*)

(13) $$F_{\mathrm{EN}}\left(V_{i},t_{i}\right)=\left\{\begin{array}{c} I_{\mathrm{EN}-\mathrm{EP}}=f_{\mathrm{EN}-\mathrm{EP}}\left(V_{i},t_{i}\right)=\beta \left(V,i\right)\cdot \left(-e^{-\alpha \left(V,i\right)\cdot t}+1\right),\\ \;t=0\;\left(\mathrm{pulse}\_\mathrm{on}\right)\;\mathrm{to}\;t=t_{\mathrm{pulse}\_\mathrm{off}}\\ I_{\mathrm{EN}-\mathrm{ED}}=f_{\mathrm{EN}-\mathrm{ED}}\left(V_{i},t_{i}\right)=I_{\mathrm{EN}-\mathrm{EP}}\left(V_{i},t_{i}\right)\cdot e^{-\gamma t_{\mathrm{pulse}\_\mathrm{off}}},\\ \;t=t_{\mathrm{pulse}\_\mathrm{off}}\;\mathrm{to}\;t_{i} \end{array}\right.$$

γ→∞

where *EP* is the potentiation process, *E* *D* is the depression process, *α* is the rise factor, *γ* is the decay factor, $t_{pulse\_on}$ is the duration where the electrical pulse in on, $t_{pulse\_off}$ is the duration where the electrical pulse is off, *β* is the amplitude of the device current at *i^th^* step.

*Device* *Algorithm* *for* *Comparing* *Function* (0 − *T*)

(14) $$f_{\mathrm{comp}\mathrm{aring}}\left(V\right)=\left\{\begin{array}{c} 1,\;V\geq V_{\mathrm{th}}\;\mathrm{and}\;F_{\mathrm{EN}}\left(V,t\right)\geq I_{\mathrm{th}}\\ 0,\;\mathrm{othe}\mathrm{rwise} \end{array}\right.$$

where *V_th_* is the threshold voltage and *I_th_* the required threshold current for firing. This device‐level comparison algorithm enables above‐threshold signal selection and final decision‐making in 1R‐cell array configurations for applied systems.

A summary of the basic behaviors of the four sensory computing modes, along with the corresponding implementable algorithms and applications, is presented in Table 1.

### 2.2 Cell and Array‐Level Reconfigurability

Based on the device‐level reconfigurability, we further experimentally demonstrate a reconfigurable system hardware prototype with cell‐ and array‐level reconfigurability, utilizing the multi‐paradigm device array and reconfigurable circuitry (Figure 2e–h). Figure 2e schematically illustrates the reconfigurable cell array architecture, where each cell integrates a multi‐paradigm device and a reconfigurable mode‐control circuit. The reconfigurable mode‐control circuit consists of an NMOS transistor and a low‐power, leakage‐based amplifier (Figure 2e). This circuit provides tuneable device biases by operating as a voltage clamp via negative feedback loops, maintaining mode‐specific biases: 0 V (PN mode), −0.1 to −0.04 V (PS mode), 0.1 to 0.5 V (ES mode), and >1.3 V (EN mode). During voltage clamping, simultaneous device readout is achieved by utilising the mode‐control circuit as a transimpedance amplifier (TIA) for current‐to‐voltage conversion. Additionally, the circuit supports different cell array architecture reconfigurations, including 1R‐cell array (PN/PS/EN modes) (Figure 2f) and 1T1R cross‐point cell array (ES mode) configurations (Figure 2j). When configured to 1T1R configuration, the amplifier is disabled to form a 1T1R cell unit for cell in ES mode, and the entire array can form a 1T1R structure for MAC operations. This dedicated circuit design maintains a unified architecture, which not only enables individual control of all four sensory modes per device but also supports flexible array configurations, thereby enabling further system‐level visual algorithms. The board‐level hardware prototype in Figure 2h is used for functional verification for this reconfigurable cell array.

Figure 2i–p experimentally validates the cell design's full access to four sensory modes and array architecture reconfigurability by presenting outputs from both single reconfigurable cells and a 12 × 12 cell array across operational modes, completely readout by the prototype platform. For PN mode (Figure 2i), continuous light illumination at 520 and 450 nm generates wavelength‐dependent optical LIF spiking. The 12 × 12 cell array's spiking output under 520 nm illumination further verifies 1R‐cell array functionality with good uniformity (Figure 2j). A PS mode cell demonstrates the behavior of pulse intensity‐dependent nonlinear synaptic plasticity and encoding capability (Figure 2k). The 12 × 12 cell array, when reconfigured to PS mode (Figure 2l) responds uniformly to the “001” pattern, generating 144 matching voltage amplitudes under a 1R architecture. An ES mode cell (Figure 2m) exhibits non‐volatile, multilevel resistive switching, as well as multiplication capability. Furthermore, the corresponding cell outputs in the array and MAC results when the array is configured in a 1T1R architecture are examined, as shown in Figure 2n, indicating uniform and effective processing capability. EN mode cell exhibits amplitude‐dependent LIF behavior under electrical pulses (1.3–2.0 V), with firing activation exclusively at 2.0 V (Figure 2o).  Corresponding array outputs (Figure 2p) show effective firing across 144 device cells when stimulated at 2.0 V, exceeding the firing threshold uniformly. Further details on the cycle‐to‐cycle and device‐to‐device performance variations are provided in Figures S13 and S14. This verified device‐to‐array reconfigurability enables the deployment of diverse system‐level NI/AI algorithms that combine spiking and non‐spiking paradigms on the prototype platform.

### 2.3 System‐Level Reconfigurability

**FIGURE 3:**
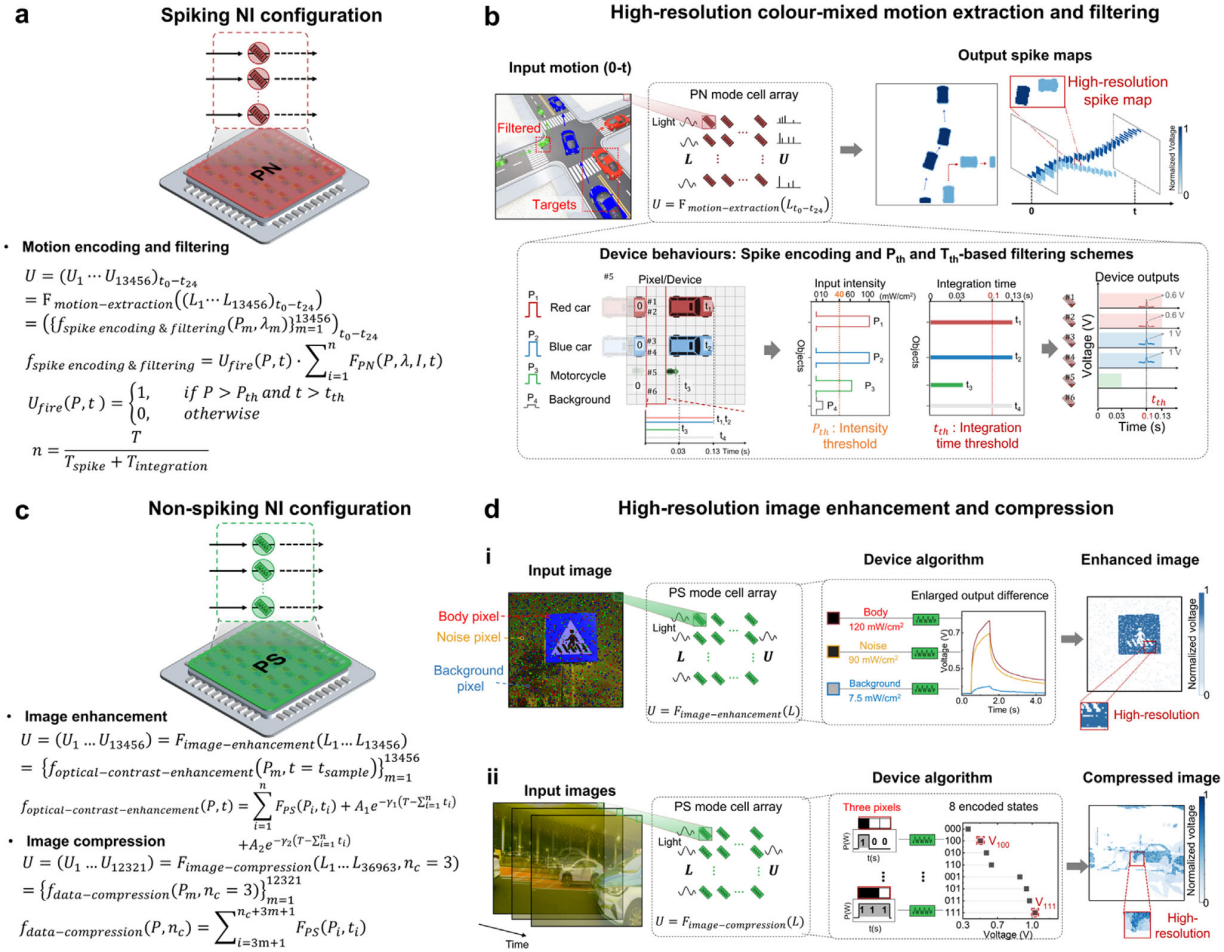
NI‐centric configurations and visual algorithms for imaging‐oriented scenarios. (a) Spiking NI configuration with all reconfigurable cells in the array configured into PN mode and the corresponding motion encoding and filtering algorithms. (b) Scenarios and output spike map results of event‐based color‐mixed motion encoding and filtering. (c) Non‐spiking NI configuration with all cells configured into PS mode and the corresponding image enhancement and compression algorithms. (d) (i) Image enhancement results, and (ii) image compression results.

**FIGURE 4:**
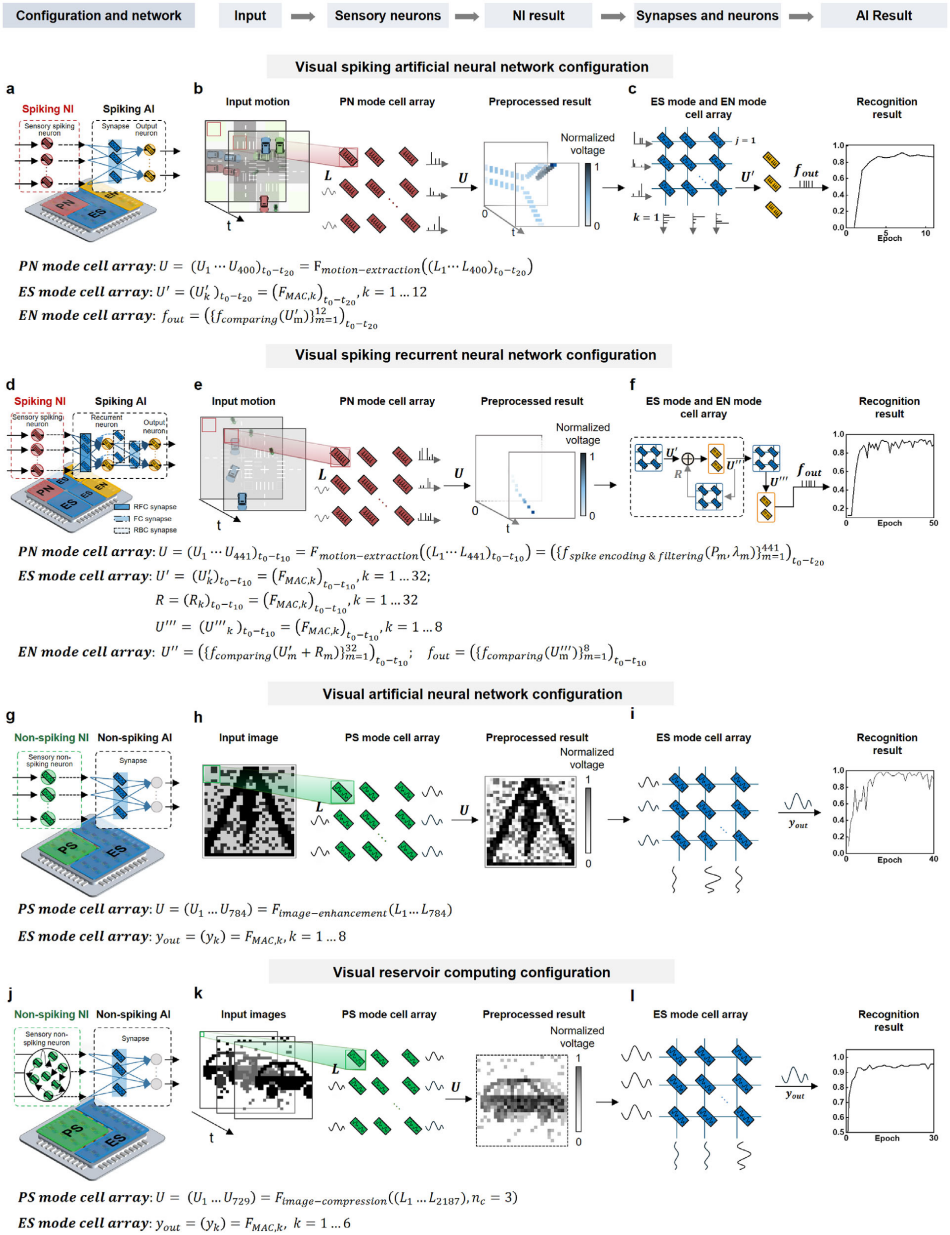
NI/AI hybrid configurations and visual algorithms for computing‐oriented scenarios. (a) Visual spiking artificial neural network configuration, wherein the cell array is configured into a PN mode cell array as the input sensory spiking neurons for the spiking NI, an ES mode cell array, and an EN mode cell array as the synapses and output neurons for the spiking AI. (b) NI‐based processing: illustration of the event‐based color‐mixed motion trajectory extraction and filtering enabled by the PN mode cell array, outputting spike maps with filtered and extracted motion trajectories (0‐T). (c) AI‐based processing: recognition accuracy of the configuration on 12 classes of mixed‐color vehicle motion images for the target cars. (d) Visual spiking recurrent neural network configuration, wherein the cell array is configured into a PN mode cell array as sensory spiking neurons for the spiking NI, an ES mode cell array, and an EN mode cell array for the spiking AI. (e) NI‐based processing: illustration of the event‐based target vehicle motion trajectory extraction and static dark background filtering, outputting spike maps with extracted and filtered motion trajectory (0‐T). (f) AI‐based processing: the prediction accuracy of the trained configuration on the future motion direction. (g) Visual artificial neural network configuration, wherein the cell array is configured into a PS mode cell array as the input sensory non‐spiking neurons for the non‐spiking NI and an ES mode cell array as the synapses for the AI. (h) NI‐based processing: illustration of the image enhancement enabled by PS mode cell array, outputting traffic sign image with enhanced body/background and body/noise contrasts. (i) AI‐based processing: classification accuracy of the trained visual artificial neural network configuration on eight categories of noisy traffic sign images. (j) Visual reservoir computing configuration, wherein the cell array is configured into a PS mode cell array as the sensory non‐spiking neurons for the non‐spiking NI, and an ES mode cell array as the synapses for the AI. (k) NI‐based processing: illustration of the image compression enabled by PS mode cell array, reducing the output image size to one‐third. (l) AI‐based processing: classification accuracy of the trained visual reservoir computing configuration on six categories of noisy traffic images.

**FIGURE 5:**
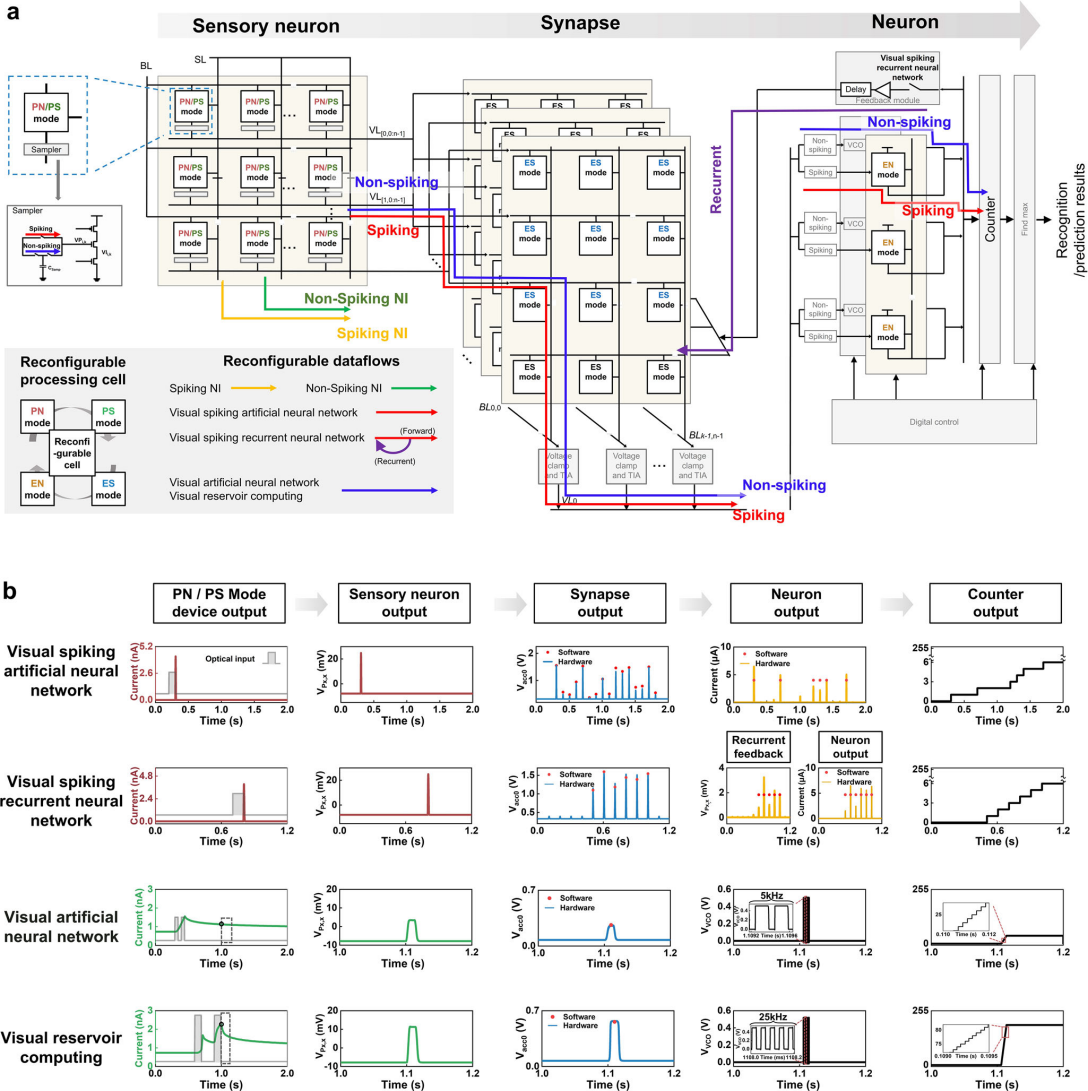
System‐level reconfigurability: reconfigurable vision system with device‐, cell‐, array‐ to system‐level reconfigurability. (a) Reconfigurable dataflow architecture, fully supporting spiking/non‐spiking/NI/AI processing and all six configurations. (b) Hardware simulation results of visual spiking artificial neural network, visual spiking recurrent neural network, visual artificial neural network, and visual reservoir computing modes.

Exploiting the behaviors in the reconfigurable cell array prototype, an on‐demand reconfigurable intelligent vision system is demonstrated. Firstly, system‐level visual algorithms are designed and results are simulated, as shown in Figures 3 and 4, based on the experimentally characterized behavior of the hardware prototype (Figure 2). Then, the system‐level architecture with reconfigurable signal paths and corresponding peripheral circuits that can fully support the system‐level algorithms is further designed for full hardware implementation verification, as shown in Figure 5.

#### 2.3.1 Imaging‐Oriented NI‐Centric Configurations

Toward high‐resolution smart imaging scenarios, the systems are reconfigured into spiking and non‐spiking NI‐centric architecture for frame‐free motion and frame‐based static processing, as shown in Figure 3. In these types of configurations, the cell array with in‐sensor computing capabilities can perform high‐resolution imaging with preprocessing. The frame rate and latency metrics for each NI‐centric configuration are provided in Table S5. Figure 3a,b shows the spiking NI configuration with all cells operate in PN mode and the demonstration of color‐mixed motion extraction and filtering task. Based on the device's wavelength‐dependent LIF spiking characteristics with intensity and integration time‐based thresholds, the PN mode cell array can intelligently capture, extract and encode the continuous motion information (*L*, from 0 to *T* period) of the colored targeted vehicles (red and blue cars), outputting high‐resolution voltage spike maps (*U*, from 0 to *T* period) with continuous trace and detailed object contour while filtering out the noisy vehicles (green motorcycle) and the static background. These motion capturing, extraction, and encoding processes are presented as:

(15) $$\begin{array}{ccc} U={\left(U_{1}\cdots U_{13456}\right)}_{t_{0}-t_{24}} & = & F_{\mathrm{moti}\mathrm{on}-\mathrm{extr}\mathrm{acti}\mathrm{on}}\;\left({\left(L_{1}\cdots L_{13456}\right)}_{t_{0}-t_{24}}\right)\\ & = & {\left({\left\{f_{\mathrm{encoding}\&\mathrm{filtering}}\left(P_{m},\;\lambda_{m}\right)\right\}}_{m=1}^{13456}\right)}_{t_{0}-t_{24}} \end{array}$$

with each device complied with the device‐level spike encoding and filtering algorithm $f_{\mathrm{encoding}\&\mathrm{filtering}}$ Equation (2), *m* is the device index.

Specifically, owing to the light‐intensity‐threshold and integration‐time‐threshold‐based firing *U_fire_* (*P*,*t*) Equation (3) in the PN mode device, the targeted moving vehicles only with both above‐threshold input intensity (*P*_1_,*P*_2_ > *P_th_*) and integration time (*t*_1_,*t*_2_ > *t_th_*) can be extracted by the device, generating spikes. Conversely, static dark background with the below‐threshold input intensity (*P*_4_ *< P_th_* |*t*_4_ > *t_th_*) and noisy motorcycle with a shorter integration time (*t*_3_ < *t_th_* |*P*_3_ > *P_th_*) result in no spikes and can be effectively filtered out. The motion information of detected targeted moving cars with different colors is consequently extracted and encoded into spikes with wavelength‐dependent amplitudes based on the device's capability represented as $f_{\mathrm{encoding}\&\mathrm{filtering}}$ Equation (2), where the spike amplitude is a function of the input wavelength *λ*. The resulting spike maps over time are illustrated in Figure 3b. The system can accurately extract and encode motion information into a frame‐free representation, faithfully representing the spatial features and temporal dynamics of the target objects.

In contrast, the devices and cells in non‐spiking NI configuration all operate in PS mode (Figure 3c), enabling high‐resolution frame‐based imaging and preprocessing including image contrast enhancement and image compression (Figure 3d). The image enhancement implemented with the PS mode array is demonstrated in Figure 3d‐(i), implemented with the device‐level optical contrast enhancement capability represented as *f*_*optical*−*contrast*−*enhancement*_(*P*, *t*) Equation (5). With the input image *L*, the PS mode device generates a slower‐decaying current for the body pixels than that for the noise pixels. This difference in decay rates enhances the contrast between the voltage signals from the body pixels and noise pixels at the sampling time. Meanwhile, it generates a rapidly‐decaying current for the background pixels, resulting in no current at the sampling time (*t_sample_*), therefore enhancing the contrast between the body and background pixels and improving the quality of the traffic sign image. The image enhancement process is described as:

(16) $$\begin{array}{ccc} U & = & F_{\mathrm{image}-\mathrm{enha}\mathrm{ncem}\mathrm{ent}}\left(L\right)\\ & = & {\left\{f_{\mathrm{opti}\mathrm{cal}-\mathrm{cont}\mathrm{rast}-\mathrm{enha}\mathrm{ncem}\mathrm{ent}}\left(P_{m},t=t_{\mathrm{samp}\mathrm{le}}\right)\right\}}_{m=1}^{13456} \end{array}$$

Figure 3d‐(ii) shows the vehicle image compression demonstration with a threefold data volume reduction enabled by the PS mode cell array. The image compression process *F*_*image*−*compression*_ is represented with the following algorithm as:

(17) $$U=F_{\mathrm{image}-\mathrm{comp}\mathrm{ress}\mathrm{ion}}\left(L\right)={\left\{f_{\mathrm{data}-\mathrm{comp}\mathrm{ress}\mathrm{ion}}\left(P_{m},n_{c}=3\right)\right\}}_{m=1}^{12321}$$

where each device complied with the device‐level data‐compression algorithm *f*_*data* − *compression*_(*P*, *n_c_*) (as detailed in Equations ((6), (7), (8))), and *m* and *n_c_* denotes the device index and the compression ratio, respectively. Specifically, during the image compression implementation, each device receives and processes a 3‐bit light information from three adjacent frames of the grayscale vehicle image stream, and compresses it into a 1‐bit conductance state, denoted as *f*_*data*−*compression*_(*P_m_*, *n_c_* =  3) for a device indexed by *m*. Therefore, the cell array can encode eight different 3‐bit optical streams ranging from “000” to “111” into eight distinct 1‐bit conductance states, thereby compressing the input images.

#### 2.3.2 AI Computing‐Oriented Hybrid NI/AI Configurations

Figure 4a–l demonstrates the AI computing‐oriented hybrid NI/AI configurations and co‐designed algorithms, including visual spiking recurrent neural network, visual spiking artificial neural network, visual artificial neural network, visual reservoir computing configurations, for accurate recognition scenarios. The computing‐oriented hybrid NI/AI configurations is designed with an NI/AI hierarchical architecture, featuring NI‐based imaging with low‐ to mid‐level processing, followed by AI‐based high‐level processing.

#### 2.3.3 Visual Spiking Artificial Neural Network (V‐SANN) Configuration

Figure 4a–c shows the visual spiking artificial neural network configuration for event‐based color‐mixed motion trajectory extraction, filtering, and recognition. The cell array is reconfigured into a PN mode cell array as the input sensory spiking neurons for the spiking NI, an ES mode cell array and an EN mode cell array as the synapses and output neurons for AI. More details about the network structure and hardware implementation can be found in Figure S15, with example scenarios in Figure S16.

As shown in Figure 4b, the PN mode cell array first extracts and encodes the continuous color‐mixed motion information (*L*, 0 − *T*) of target vehicles (cars) while filtering out the noisy motorcycle and static background based on the schemes depicted in Figure 3b. The output is a voltage spike map $U={\left(U_{1}\text{…}U_{400}\right)}_{t_{0}-t_{20}}$ with extracted motion trajectories. This process is represented as:

(18) $$U={\left(U_{1}\cdots U_{400}\right)}_{t_{0}-t_{20}}=F_{\mathrm{moti}\mathrm{on}-\mathrm{extr}\mathrm{acti}\mathrm{on}}\left({\left(L_{1}\cdots L_{400}\right)}_{t_{0}-t_{20}}\right)$$

These voltage spike maps from the PN mode cell array are subsequently input to the ES mode cell array for MAC operations (Figure S17). The MAC algorithm is represented as:

(19)

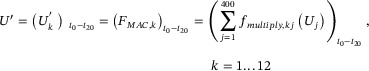

where each ES mode device performs a device‐level multiplication algorithm *f_multiply_*, as detailed in Equation (12). *k* is the column index, and *j* is the row index. The output voltages spikes are then processed by the EN mode cell array for selecting the above‐threshold signals and final decision‐making (Figure 4c). The final output voltage spike trains are described as
(20)

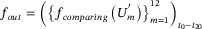

where each device complied with the device‐level algorithm *f_comparing_* Equation (14).

The spike rates of the EN mode cell array $\frac{f_{out}}{T}$ indicate the recognition result (Figure S18). The trained system achieves a recognition accuracy of 91.7% across 12 mixed‐color vehicle motion classes. Further details of the dataset and the signal flow are shown in Note S3.

#### 2.3.4 Visual Spiking Recurrent Neural Network (V‐SRNN) Configuration

Figure 4d–f shows the visual spiking recurrent neural network configuration for event‐based target vehicle (car) motion trajectory extraction and prediction. This configuration reconfigures the cell array into a PN mode cell array for spiking NI, and ES and EN mode cell arrays for spiking AI. The PN mode cell array serves as the sensory spiking neurons. The ES mode cell array serves as synapses, including recurrent forward‐connection (RFC) synapses, recurrent backward‐connection (RBC) synapses, and fully connected (FC) synapses, while the EN mode cell array serves as recurrent neurons and output neurons. This configuration processes ten continuous motion steps and predicts future motion directions at 11th step. Details of the network structure are shown in Figure S19, and examples of scenarios are shown in Figure S20.

As shown in Figure 4e, the PN mode cell array encodes the motion data from input motion image *L* into voltage spike signals and simultaneously filters the static background (Figure 3b), outputting voltage spike maps $\left(U={\left(U_{1}\text{…}U_{441}\right)}_{t_{0}-t_{10}}\right)$ with extracted motion trajectory. This process is represented as:

(21) $$U={\left(U_{1}\cdots U_{441}\right)}_{t_{0}-t_{10}}=F_{\mathrm{moti}\mathrm{on}-\mathrm{extr}\mathrm{acti}\mathrm{on}}\left({\left(L_{1}\cdots L_{441}\right)}_{t_{0}-t_{10}}\right)$$

The resulting voltage spike maps *U* are then fed to the ES mode cell arrays for MAC (Figure S21) and the EN mode cell arrays for final prediction (Figure 4f). The MAC process for ES mode cell array (row index *j*, column index *k*) is represented as:

(22)

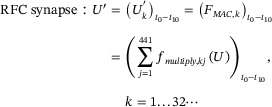

(23) $$\begin{array}{ccc} \mathrm{RBC}\;\mathrm{synapse}:R & = & {\left(R_{k}\right)}_{t_{0}-t_{10}}={\left(F_{MAC,k}\right)}_{t_{0}-t_{10}}\\ & = & {\left(\sum_{j=1}^{32}f_{multiply,kj}\left(U^{\prime \prime }\right)\right)}_{t_{0}-t_{10}},\\ & & k=1\text{…}32 \end{array}$$

(24) $$\begin{array}{ccc} \mathrm{FC}\;\mathrm{synapse}:\;U^{\prime \prime \prime } & = & {\left({U^{\prime \prime \prime }}_{k}\right)}_{t_{0}-t_{10}}\\ & = & {\left(F_{MAC,k}\right)}_{t_{0}-t_{10}}={\left(\;\sum_{j=1}^{32}f_{multiply,kj}\left(U^{\prime \prime }\right)\;\right)}_{t_{0}-t_{10}},\\ & & k=1\text{…}8 \end{array}$$

where *U*′, *R* and *U*′′′represent the MAC output voltage spikes for RFC synapses, RBC synapses and FC synapses, respectively.

The predicted motion direction of the 11^th^ step is determined by the spike rates of the EN mode output neurons. (Details can be found in Figure S22). This configuration achieves a prediction accuracy of 92.5%. Additional information on the dataset and processing workflow can be found in Note S4.

#### 2.3.5 Visual Artificial Neural Network (V‐ANN) Configuration

Figure 4g–i demonstrates the non‐spiking visual artificial neural network configuration for image enhancement and classification, wherein the cell array is configured into a PS mode cell array as the input sensory spiking neurons for the non‐spiking NI, and an ES mode cell array as the synapses for AI. The network architecture is detailed in Figure S23, with scenario examples provided in Figure S24.

As shown in Figure 4h, the PS mode cell array first in situ performs the contrast enhancement of the noisy traffic sign images *L* according to the processing schemes in Figure 3d, resulting in a voltage map $U=(U_{1}\text{…}U_{784})$. This contrast enhancement process is expressed as:

(25) $$U=\left(U_{1}\cdots U_{784}\right)=F_{\mathrm{image}-\mathrm{enha}\mathrm{ncem}\mathrm{ent}}\left(L_{1}\cdots L_{784}\right)\;$$

The voltage map *U* from the PS mode cell array is then fed into the ES mode cell array for MAC operations (Figure S25), outputting voltage signals *y_out_* with different amplitudes (Figure 4i). The highest amplitude indicates the final classified category. The MAC process for each ES mode cell array (row index *j*, column index *k*) in this configuration is represented as:

(26) $$y_{out}=\left(y_{k}\right)=F_{MAC,k}=\sum_{j=1}^{784}f_{multiply,kj}\left(U_{j}\right)\;,k=1\text{…}8$$

The trained visual artificial neural network correctly recognizes eight categories of noisy traffic sign images with a high accuracy of 97%. This represents an 18% improvement in classification accuracy compared with the system without non‐spiking NI‐based low‐level processing (Figure S26). Note S5 provides detailed information on the processing workflow.

#### 2.3.6 Visual Reservoir Computing (V‐RC) Configuration

Figure 4j–l demonstrates the non‐spiking visual reservoir computing configuration for image compression and classification, wherein the cell array is reconfigured into a PS mode cell array as the sensory non‐spiking neurons for the non‐spiking NI, and an ES mode cell array as the synapses for the reservoir computing‐based AI. Details of the network structure are shown in Figure S27. The PS mode cell array reduces the data volume of dynamic traffic images *L* (27 × 27 × 3) (Figure S28) based on the processing schemes depicted in Figure 3d, outputting the compressed image in the form of voltage map. The image compression process is expressed as:

(27) $$U=\left(U_{1}\cdots U_{729}\right)=F_{\mathrm{image}-\mathrm{comp}\mathrm{ress}\mathrm{ion}}\left(\left(L_{1}\cdots L_{2187}\right),n_{c}=3\right)$$

The voltage maps *U* from the PS mode cell array are fed into the ES mode cell array for MAC (Figure S29), generating output voltage signals *y_out_* (Figure 4l). The MAC process for each ES mode cell array (row index *j*, column index *k*) in this configuration is represented as:

(28) $$y_{\mathrm{out}}=\left(y_{k}\right)=F_{\mathrm{MAC},k}=\sum_{j=1}^{729}f_{\mathrm{mult}\mathrm{iply},\mathrm{kj}}\left(U_{j}\right)\;,k=1\cdots 6$$

After training, the visual reservoir computing configuration classifies all six categories of noisy traffic images with an accuracy of 95.8%. Compared to systems without NI‐based processing, the NI‐based image compression in the non‐spiking visual reservoir computing configuration can reduce the data volume at the front end, leading to a further reduced network size and training complexity, while maintaining comparable recognition accuracy (Figure S27). Detailed information about the processing workflow can be found in Note S6.

#### 2.3.7 Reconfigurable System Dataflow and Architecture

To support system‐level reconfigurability in full hardware aspect, we further design a reconfigurable dataflow architecture. Figure 5 shows the full hardware design at the system level that can support all the system‐level algorithms and versatile scenarios described in Figures 3 and 4. All the circuits in this vision system are designed using a commercial 180 nm CMOS Process Design Kit (PDK) and verified through state‐of‐the‐art EDA tools simulations.

The system's dataflow initiates with the reconfigurable spiking/non‐spiking NI processing path. As shown in Figure 5a, the PN or PS mode cells serve as sensory neurons for smart imaging and low‐ and mid‐level processing. Each sensory neuron is constructed by a PN or PS mode device cell, a bypassable capacitive sampler and a driver. The bypass capacitive sampler supports reconfiguring the dataflow path for spiking and non‐spiking NI. Specifically, the non‐spiking PS mode cell output is sampled at a configurable time, while the PN mode cell output is relayed directly. The NI configuration results can then be read from the driver as sensory neuron outputs.

When the system is reconfigured for NI/AI hybrid processing, the sensory neuron outputs are then fed into the synapse module, consisting of a 1T1R‐structured ES mode cell array for efficient MAC operations. For the visual spiking recurrent neural network cases, additional recurrent paths are facilitated between the synapse and neuron modules with a configurable delay. Finally, the synapse outputs are passed to the neuron module for making a decision, which includes a dual‐path spike generator and a unified decision‐making block for support both spiking and non‐spiking signals efficiently. The dual‐path spike generator outputs spike trains using an EN mode cell during spiking processing or converts the non‐spiking voltage signals to spike trains using a voltage‐controlled oscillator (VCO) during the non‐spiking cases. A decision is thus made by counting the number of output spikes and finding the maximum one using the unified decision‐making block. A more detailed system architecture, including circuit designs, is presented in Figure S30. Figure 5b shows the hardware implementation results of system operating modes (visual spiking artificial neural network mode, visual spiking recurrent neural network mode, visual artificial neural network mode and visual reservoir computing mode), demonstrating the expected behaviors of the system.

In NI‐based processing, our system can achieve 9.1–52.6 TOPS/W energy efficiency, which is 86–107 times higher than the currently reported CMOS state‐of‐the‐art. In AI‐based processing, our system shows an energy efficiency of 2.3–2.7 TOPS/W for spiking neural networks and 76.0–76.5 TOPS/W for non‐spiking neural networks. Moreover, our system can uniquely perform hybrid NI/AI processing, demonstrating 2.3–75.5 TOPS/W energy efficiency, which is 28–922 times better than the reported CMOS state‐of‐the‐art. The area efficiency is 2.29 × 10^4^ to 7.53 × 10^5^ OPS/F^2^, which is 2986–3900 times better than current CMOS state‐of‐the‐art reported neuromorphic vision systems (Tables S6–S14 and Notes S7 and S8) [37, 38, 39, 40, 41, 42].

## 3 Conclusion

In this work, we present a bio‐inspired, intelligent vision system that merges flexibility with exceptional area and power efficiency. By employing hardware‐algorithm co‐design at both device and system levels, our system achieves hierarchical reconfigurability across device, circuit, and system levels, enabling versatile NI‐centric and NI/AI hybrid processing. This adaptability allows the system to handle diverse environmental scenarios while delivering unprecedented performance improvements over conventional AI vision chips. Our design achieves high efficiency (up to 76.5 TOPS/W) and shows promising improvements in area utilization compared to existing reconfigurable vision chips, with area efficiency enhancements reaching up to three orders of magnitude. These results suggest opportunities for advancing neuromorphic vision systems. Looking ahead, further enhancements could expand the system's capabilities to support more complex models and broader visual scenarios, such as engineering richer device dynamics by synergistically modulating electrical and ionic dynamics to support spike‐timing‐dependent plasticity for online learning, and integrating additional functional modules to support a broader set of operations (e.g., normalization and complex activation functions).

## 4 Methods

### 4.1 Device Fabrication

The two‐terminal multi‐paradigm device consists of a Pd/CuO_x_/ITO stack. The bottom electrode was first patterned using optical lithography, followed by 35 nm‐thick Pd deposited via electron‐beam evaporation and a lift‐off process. Thereafter, the sandwich layer was patterned using optical lithography, followed by 40 nm CuO_x_ layer deposited via RF sputtering using a CuO target and a lift‐off process. Finally, the top electrode was formed by optical lithography patterning, deposition of 40 nm ITO via DC sputtering, followed by lift‐off process. The same process steps were used for the device array fabrication except that the bottom electrodes of the same column of devices were connected and the top electrodes of each device are connected individually.

### 4.2 Device Characterization

The optical LIF characterization in PN mode was conducted using a laser (LL375/450/520/638/808/1064, CCRAMAN) as the light source, a transimpedance amplifier (SR570, Stanford Research Systems) for current amplification, and a digital storage oscilloscope (MDO34, Tektronix) for precise signal capture. The optical current response measurements in PS mode were performed using a laser controlled by an arbitrary waveform generator (AFG31102, Tektronix), with current signals recorded using a Keithley 2636B source meter for high‐accuracy current measurements. For the ES mode, DC voltage sweep measurements were carried out using a Keithley 2636B source meter to characterize the resistive switching. Pulse measurements were conducted using an arbitrary waveform generator (AFG31102, Tektronix) to generate electrical pulses, with current responses recorded using a Keithley 2636B source meter. In the EN mode, pulse measurements were conducted using an arbitrary waveform generator (AFG31102, Tektronix) to generate electrical pulses, with current responses amplified by a transimpedance amplifier (SR570, Stanford Research Systems) and recorded using a digital storage oscilloscope (MDO34, Tektronix) for high‐resolution temporal analysis.

### 4.3 Hardware Prototype Platform

The platform includes custom peripheral circuit boards that are able to apply mode‐specific bias voltages and measure the corresponding output currents from individual multi‐paradigm devices within the array. The boards feature 12‐bit digital‐to‐analog converters (DACs) capable of generating voltage pulses from −5 to +5 V at sampling rates over 100 kS/s to apply bias voltages. Current readout from the devices is performed with 16‐bit analog‐to‐digital converters operating at 1 MS/s. The integrated high‐speed CMOS analog switches enable dynamic switching between array architectures, with switching times below 40 ns. Core control logic is implemented on a field‐programmable gate array (FPGA) with a 50 MHz system clock. The device size in the hardware prototype platform is 25 µm × 25 µm.

### 4.4 Visual Algorithms

Details of the visual algorithm implementation and network architecture can be found in the Supporting Information.

## Conflicts of Interest

The authors declare no conflicts of interest.

## Supporting information




**Supporting File 1**: advs74029‐sup‐0001‐SuppMat.docx.

## Data Availability

The data that support the findings of this study are available from the corresponding authors upon reasonable request. All codes used in simulations supporting this article are available from the corresponding authors upon reasonable request.
