# Supplementary material for: An On‐Demand Neuromorphic Vision System Enabled by a Multi‐Paradigm Neuromorphic Device and Hierarchical Reconfigurability Designed from Device to System Level
Source: Adv Sci (Weinh). 2026 Feb 11:e20448. Online ahead of print. doi: 10.1002/advs.202520448 (PMC13325477; doi:10.1002/advs.202520448)
Supplement: Supplementary file 1 — Supporting File 1: advs74029‐sup‐0001‐SuppMat.docx. [file ADVS-9999-e20448-s001.docx]

**An On-Demand Neuromorphic Vision System Enabled by A Multi-Paradigm Neuromorphic Device and Hierarchical Reconfigurability designed from Device to System Level**

Biyi Jiang^1†^, Jiayi Xu^1†^, Liang Ran^2†^, Xinhe Feng^2^, Khalil Harrabi^3,4^, Yida Li^1*^, Longyang Lin^1*^ and Feichi Zhou^1*^

^1^School of Microelectronics, Southern University of Science and Technology, Shenzhen, China

^2^Beijing Pixelcore Technology Co., Ltd, Beijing, China

^3^Department of Physics, College of Engineering and Physics, King Fahd University of Petroleum and Minerals, Dhahran, 31261, Saudi Arabia.

^4^Interdisciplinary Research Center for Advanced Quantum Computing, King Fahd University of Petroleum and Minerals (KFUPM), Dhahran, 31261, Saudi Arabia.

# Abbreviations

| **NI** | Neuromorphic imaging |
| --- | --- |
| **AI** | Artificial intelligence |
| **PN** | Photo-spiking neuron |
| **PS** | Photo-synaptic |
| **ES** | Electrical synaptic |
| **EN** | Electrical neuron |
| **V-SANN** | Visual spiking artificial neural network |
| **V-SRNN** | Visual spiking recurrent neural network |
| **V-ANN** | Visual artificial neural network |
| **V-RC** | Visual reservoir computing |

**Supplementary Table 1 | Detailed metrics of each mode of the cell in the vision system.**

|  |  | **PN** **mode** | **PS** **mode** | **ES** **mode** | **EN** **mode** |
| --- | --- | --- | --- | --- | --- |
| **Per** **cell** | **Circuit power consumption*** | 1.10 pW | 1.10 pW | 0.00 pW | 1.10 pW |
|  | **Device read** **voltage** | 0 V | - 0.04 V | 10 mV | >1.3 V |
|  | **Device power consumption per read event** | 0.00 W | 0.42 pW | 0.20 pW | 315.00 nW |
|  | **Cell Power consumption** | 1.10 pW | 1.52 pW | 0.20 pW | 315.00 nW |

* The leakage-based amplifier in the mode-control circuit consumes only 1.1 pW for the targeted 200 Hz bandwidth, obtained from simulation using Cadence Virtuoso with a commercial 180 nm CMOS PDK.

**Supplementary Table** **2 | Calculation details of energy and power of the cell.**

| **Mode** | **Metric** | **Calculation** |
| --- | --- | --- |
| PN mode | Circuit power consumption | P = 1.10 pW |
|  | Device power consumption per read event | P = 0.00 W (zero voltage bias) |
|  | Cell power consumption | 0.00 W + 1.10 pW = 1.10 pW |
| PS mode | Circuit power consumption | P = 1.10 pW |
|  | Device power consumption per read event | P = V × I × t_read_ / T = 0.04 V × 26.00 pA × 5.00 ms / 12.50 ms = 0.42 pW |
|  | Cell power consumption | 0.42 pW + 1.10 pW = 1.52 pW |
| ES mode | Circuit power consumption | P = 0 pW |
|  | Device power consumption per read event | P = V × I = 0.01 V × 20.00 pA = 200.00 fW |
|  | Cell power consumption | 200.00 fW |
| EN mode | Circuit power consumption | P = 1.10 pW |
|  | Device power consumption per read event | P = E / t_read_ = 157. 50 pJ / 500.00 µs = 315.00 nW |
|  | Cell power consumption | 315.00 nW |

**Supplementary Table 3 | Comparisons of the state-of-the-art emerging reconfigurable neuromorphic devices with our multi-paradigm device**

|  | | ^1^ | ^2^ | ^3^ | ^4^ | **This work** |
| --- | --- | --- | --- | --- | --- | --- |
| **Configuration** | | MoTe_2_/P(VDF-TrFE) transistor | V/VO_x_/HfWO_x_/  Pt + capacitor | Phototransistor + Ta/TaO_x_/NbO_x_/W | TiN/ZnO/  TiO_x_/Pd | Pd/CuO_x_/ITO |
|  |  | 1T | 1R+1C | 1T  + 1R | 1R | 1R (single device) |
| **Reconfigurability**  **(dynamics)** | | PS+ES mode | Only ES and EN modes | Only PS and PN modes | Only PS and ES modes | **PN+PS+ES+EN modes** |
| **Energy per operation** | **PN mode** | Not support | Not support | - | Not support | **0 pJ** |
|  | **PS mode** | 0 pJ | Not support | - | 2 nJ | **152 fJ** |
|  | **ES mode** | - | 60 fJ | Not support | 2 nJ | **4 fJ** |
|  | **EN mode** | Not support | 1 nJ | Not support | Not support | **157. 50 pJ** |
| **CMOS-BEOL compatible** | | Yes | Yes | Yes | Yes | **Yes** |
| **Photoresponse** | | 340 - 1310 nm | No | 450-800 nm | 405-650 nm | **375 nm – 1064 nm** |

**Supplementary Table** **4 | Comparisons of the electrical and photoresponsive spiking neurons with our multi-paradigm device in PN mode^3,5-11^.**


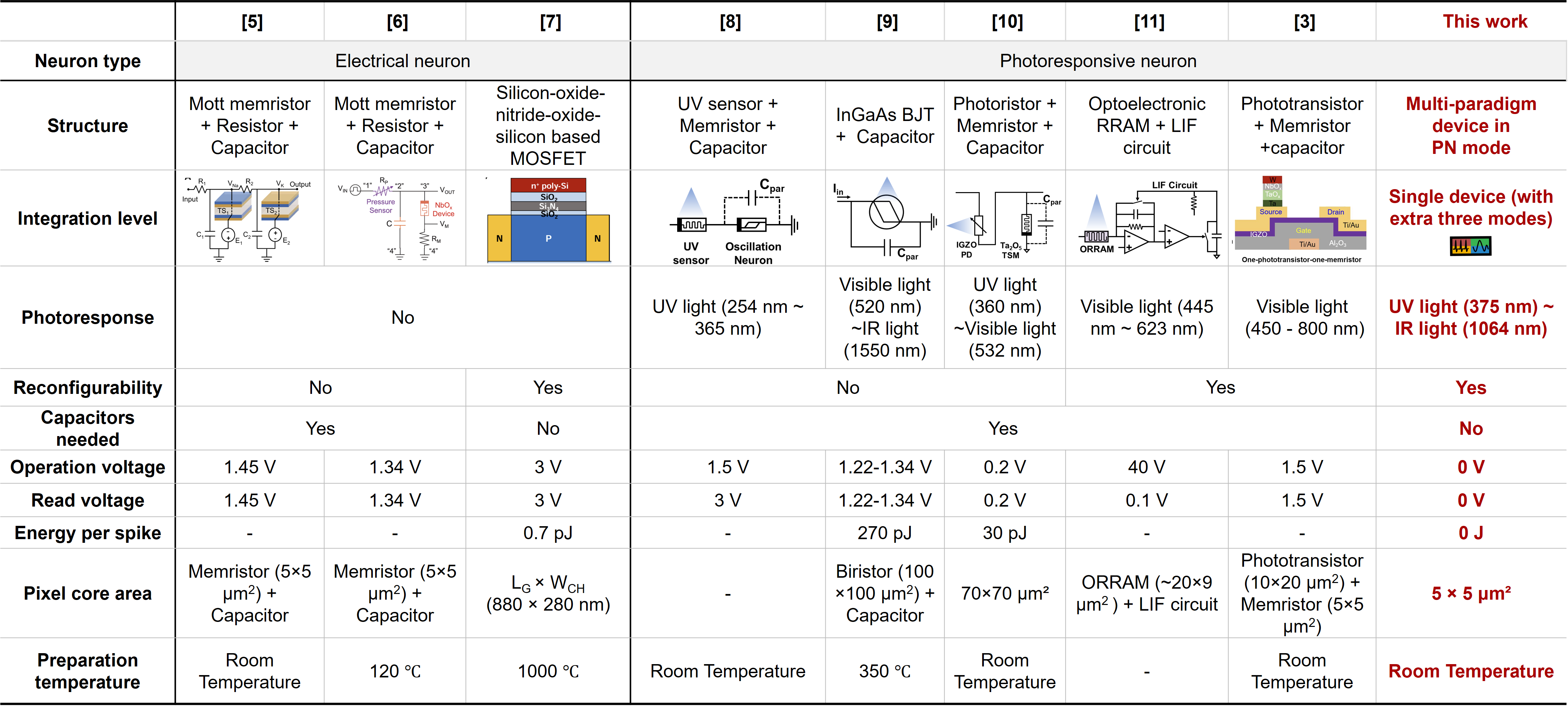


**Supplementary Table 5 | Performance metrics of NI-based processing functionalities.**

| Array configuration | NI-based processing | Frame Rate | Latency |
| --- | --- | --- | --- |
| Spiking mode | Motion extraction and filtering | 10 fps | 0.1 s |
| Non-spiking mode | Image enhancement | Up to 80 fps | ~ 12.5 ms |
|  | Image compression | 80 fps | ~ 12.5 ms |

**Supplementary Table 6 |** **Comparisons of the representative reconfigurable vision systems with our work** **^3,4,12-18^.**





* The power consumption is calculated using 5 µm × 5 µm multi-paradigm devices. Power consumption can be further reduced as the device is scaled down. The image size for spiking NI is 116 × 116, while for non-spiking NI it is 111 × 111. The image size for V-SANN (visual spiking artificial neural network configuration) is 20 × 20, for V-SRNN (visual spiking recurrent neural network configuration) is 21 × 21, for V-ANN (visual artificial neural network configuration) is 28 × 28, and for V-RC (visual reservoir computing configuration.) is 27 × 27. The V-SANN and V-SRNN are estimated at a frequency of 10Hz, while the V-ANN and V-RC are estimated at 80Hz. The power consumption of the peripheral circuits is obtained using Cadence Virtuoso @ 180nm.

** For AI-based processing, the power consumption is 84.8 nW for V-SANN, 227.5 nW for V-SRNN, 26.3 nW for V-ANN and 18.4 nW for V-RC.

*** For AI-based processing, the energy efficiency is 2.3 TOPS W^−1^ @ 10 Hz for V-SANN, 2.7 TOPS W^−1^ @ 10 Hz for V-SRNN, 76.5 TOPS W^−1^ @ 80 Hz for V-ANN and 76.0 TOPS W^−1^ @ 80 Hz for V-RC.

**** For hybrid NI/AI processing, the power consumption is 85.2 nW for V-SANN, 228 nW for V-SRNN, 27.4 nW for V-ANN, and 19.5 nW for V-RC.

***** For hybrid NI/AI processing, the energy efficiency is 2.3 TOPS W^−1^ @ 10 Hz for V-SANN, 2.7 TOPS W^−1^ @ 10 Hz for V-SRNN and 75.5 TOPS W^−1^ @ 80 Hz for V-ANN and 74.7 TOPS W^−1^ @ 80 Hz for V-RC.

**Supplementary Table 7 | Calculation Table for Spiking NI configuration.**

| **Metrics** | **Calculation details** |
| --- | --- |
| Operations per second | N_OP, PN_ = 13456 × 10 Hz = 134560 OPS |
| Power | P_Total_ = 1.1 pW × 13456 = 14.8 nW |
| Power Efficiency | Efficiency = N_OP, PN_ / P_Total_ = 134560OPS / 1.480 nW = 9.09 TOPS/W |

**Supplementary Table 8 | Calculation Table for Non-spiking NI configuration.**

| **Metrics** | **Calculation details** |
| --- | --- |
| Operations per second | N_OP, PS_ = 12321 × 80 Hz = 985680 OPS |
| Power | P_Total_ = 1.52 pW × 12321 = 18.73 nW |
| Power Efficiency | Efficiency = N_OP, PS_ / P_Total_ = 985680 / 18.73 nW = 52.63 TOPS/W |

**Supplementary Table 9 | Calculation Table for visual spiking artificial neural network configuration.**

| **Metrics** | **Calculation details** | | |
| --- | --- | --- | --- |
| Operations per second | NI-based processing | PN mode cell array | N_OP, PN_ = 400 × 1 OP × 10 Hz = 4000 OPS |
|  | AI-based processing | ES mode cell array | N_OP, ES_ = (4800 × 2 × 2) OP × 10 Hz = 192000 OPS |
|  |  | EN mode cell array | N_OP, EN_ = 12 × 1 OP = 12 OPS |
|  |  | Digital circuits | N_OP, Digital circuits_ = 24 × 1 OP × 10 Hz = 240 OPS |
|  | Total | | N_OP, Total_ = N_OP, PN_ + N_OP, ES_ + N_OP, EN_ + N_OP, Digital circuits_ = 196252 OPS |
| Power | NI-based processing | PN mode cell array | P_PN_ = 1.10 × 10^-12^ W × 400 =0.44 nW |
|  | AI-based processing | ES mode cell array | P_ES_ = (4800 × 2/10 × 19 pW) + (12 × 291 pW) = 0.217 µW |
|  |  | EN mode cell array | P_EN_ = 0.2 × 315 nW + 12 × 1.1 pW = 63 nW |
|  |  | Digital circuits | 24.72 pW |
|  | Total | | P_Total_ = P_PN_ + P_ES_ + P_EN_ + P_digital_circuits_ = 85.2 nW |
| Power efficiency | NI-based processing | | N_OP, PN_ / P_PN_ = 4000 OPS / 0.44 nW = 9.09 TOPS/W |
|  | AI-based processing | | (N_OP, ES_ + N_OP, EN_ + N_OP,Digital circuits_) / (P_ES_ + P_EN_ + P_digital_circuits_) = 192252 OPS / 84.8 nW = 2.27 TOPS/W |
|  | Hybrid NI/AI computing | | N_OP, Total_ / P_Total_ = 196252 OPS / 85.2 nW = 2.30 TOPS/W |

**Supplementary Table 10 | Calculation Table for visual spiking recurrent neural network configuration.**

| **Metrics** | **Calculation details** | | |
| --- | --- | --- | --- |
| Operations per second | NI-based processing | PN mode cell array | N_OP, PN_ = 441 × 1 OP × 10 Hz = 4410 OPS |
|  | AI-based processing | ES mode cell array | N_OP, ES_ = (14112 + 1024 + 256) × 2 × 2 OP × 10 Hz = 615680 OPS |
|  |  | EN mode cell array | N_OP, EN_ = (32 + 8) × 1 OP = 40 OPS |
|  |  | Digital circuits | N_OP, Digital circuits_ = 16 × 1 OP × 10 Hz = 160 OPS |
|  | Total | | N_OP, Total_ = N_OP, PN_ + N_OP, ES_ + N_OP, EN_ + N_OP, Digital circuits_ = 620290 OPS |
| Power | NI-based processing | PN mode cell array | P_PN_ = 1.10 × 10^-12^ W × 441 = 485 pW |
|  | AI-based processing | ES mode cell array | P_ES_ = P_ES_RFC_ + P_ES_RBC_ + _PES_FC_ = (14112 × 2/10 × 19 pW) + (1024 × 2/10 × 19 pW) + (32 × 291 pW) + (256 × 2/10 × 19 pW) + (8 × 291 pW) = 70.1 nW |
|  |  | EN mode cell array | P_EN_ = 32 × 0.125 × 0.1 × 315 nW + 32 × 1.1 pW + 8 × 0.125 × 0.1 × 315 nW + 8 × 1.1 pW = 158 nW |
|  |  | Digital circuits | 16.48 pW |
|  | Total | | P_Total_ = P_PN_ + P_ES_ + P_EN_ + P_digital_circuits_ = 228 nW |
| Power efficiency | NI-based processing | | N_OP, PN_ / P_PN_ = 4410 OPS / 485 pW = 9.09 TOPS/W |
|  | AI-based processing | | (N_OP, ES_ + N_OP, EN_ + P_digital_circuits_) / (P_ES_ + P_EN_ + P_digital_circuits_) = 615880 OPS / 228 nW = 2.70 TOPS/W |
|  | Hybrid NI/AI computing | | N_OP, Total_ / P_Total_ = 620290 OPS / 228 nW = 2.72 TOPS/W |

**Supplementary Table 11 | Calculation Table for visual artificial neural network configuration.**

| **Metrics** | **Calculation details** | | |
| --- | --- | --- | --- |
| Operations per second | NI-based processing | PS mode cell array | N_OP, PS_ = 784 × 1 OP × 80 Hz = 62720 OPS |
|  | AI-based processing | ES mode cell array | N_OP, ES_ = (6272 × 2 × 2) OP × 80 Hz = 2007040 OPS |
|  |  | Digital circuits | N_OP, Digital circuits_ = 24 × 1 OP × 80 Hz = 1920 OPS |
|  | Total | | N_OP, Total_ = N_OP, PS_ + N_OP, ES_ + N_OP, Digital circuits_ = 2071680 OPS |
| Power | NI-based processing | PS mode cell array | P_PS_ = 1.52 × 10^-12^ W × 784 = 1.19 nW |
|  | AI-based processing | ES mode cell array | P_ES_ = (6272 × 2/10 × 19 pW) + (8 × 291 pW) = 26.2 nW |
|  |  | Digital circuits | 92.48 pW |
|  | Total | | P_Total_ = P_PS_ + P_ES_ + P_digital_circuits_ = 27.4 nW |
| Power efficiency | NI-based processing | | N_OP, PS_ / P_PS_= 62720 OPS / 1.19 nW = 52.63 TOPS/W |
|  | AI-based processing | | (N_OP, ES_ + N_OP, Digital circuits_) / (P_ES_ + P_digital_circuits_) = 2008960 OPS / 26.3 nW = 76.52 TOPS/W |
|  | Hybrid NI/AI computing | | N_OP, Total_ / P_Total_ = 2071680 OPS / 27.4 nW = 75.48 TOPS/W |

**Supplementary Table** **12 | Calculation Table for visual reservoir computing configuration.**

| **Metrics** | **Calculation details** | | |
| --- | --- | --- | --- |
| Operations per second | NI-based processing | PS mode cell array | N_OP, PS_ = 729 × 1 OP × 80 Hz = 58320 OPS |
|  | AI-based processing | ES mode cell array | N_OP, ES_ = (4374 × 2 × 2) OP × 80 Hz = 1399680 OPS |
|  |  | Digital circuits | N_OP, Digital circuits_ = 18 × 1 OP × 80 Hz = 1440 OPS |
|  | Total | | N_OP, Total_ = N_OP, PS_ + N_OP, ES_ + N_OP, Digital circuits_ = 1459440 OPS |
| Power | NI-based processing | PS mode cell array | P_PS_ = 1.52 pW × 729 = 1.11 nW |
|  | AI-based processing | ES mode cell array | P_ES_ = (4374 × 2/10 × 19 pW) + (6 × 291 pW) = 18.4 nW |
|  |  | Digital circuits | 68.16 pW |
|  | Total | | P_Total_ = P_PS_ + P_ES_ + P_digital_circuits_ = 19.5 nW |
| Power efficiency | NI-based processing | | N_OP, PS_ / P_PS_ = 58320 OPS / 1.11 nW = 52.63 TOPS/W |
|  | AI-based processing | | (N_OP, ES_ + N_OP, Digital circuits_) / (P_ES_ + P_digital_circuits_) = 1401120 OPS / 18.4 nW = 76.0 TOPS/W |
|  | Hybrid NI/AI computing | | N_OP, Total_ / P_Total_ = 1459440 OPS / 19.5 nW = 74.68 TOPS/W |

**Supplementary Table 13 | Calculation Table for area efficiency.**

| **Metric** | **Min Value** | **Max Value** |
| --- | --- | --- |
| Operations per Second per Watt (OPS/W) | 2.3 × 10^12^ | 7.55 × 10^13^ |
| Total Area (um^2^) | 3.2495 × 10^6^ | |
| Scaled Area (F^2^) | 1.0029 × 10^8^ | |
| Area Efficiency (OPS/F^2^) = Operations Per second (OPS) / Feature size (F^2^) | 2.2933 × 10^4^ | 7.5279 × 10^5^ |

**Supplementary Table** **14 | Detailed metrics of the system performances.**

|  | **Spiking NI** | **Non-spiking NI** | **V-SANN** | **V-SRNN** | **V-ANN** | **V-RC** |
| --- | --- | --- | --- | --- | --- | --- |
| **System power consumption** | 14.8 nW | 18.7 nW | 85.2 nW | 228.2 nW | 27.4 nW | 19.5 nW |
| **Energy efficiency** | 9.1 TOPSW | 52.6  TOPS/W | 2.3 TOPS/W | 2.7 TOPS/W | 75.5 TOPS/W | 74.7 TOPS/W |


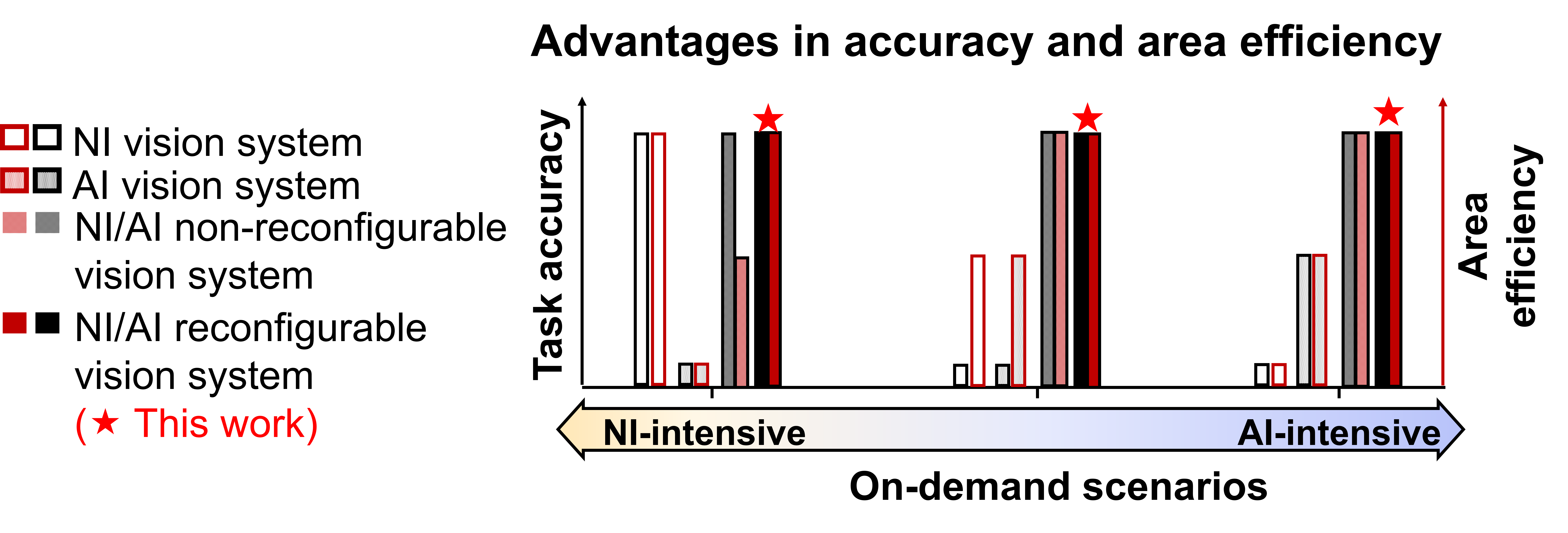


Supplementary Fig. 1 | Comparisons of the task accuracy and area efficiency of the NI/AI reconfigurable and other NI/AI nonreconfigurable intelligent vision systems. Our proposed system with spiking/non-spiking/NI/AI reconfigurability across device to system levels exhibits advantages in task accuracy as well as area efficiency.


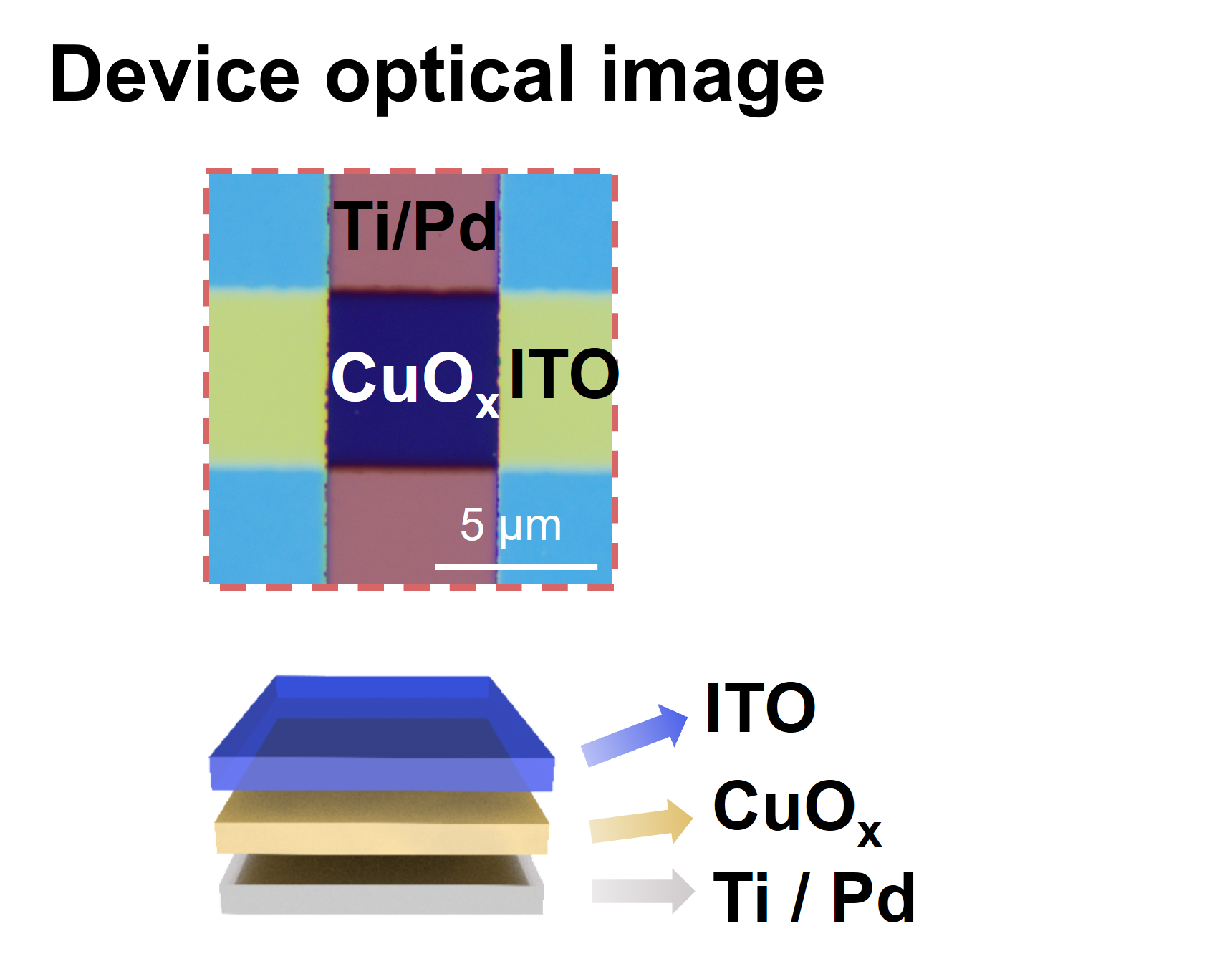


Supplementary Fig. 2 | Optical image of the multi-paradigm Pd/CuO_x_/ITO device.


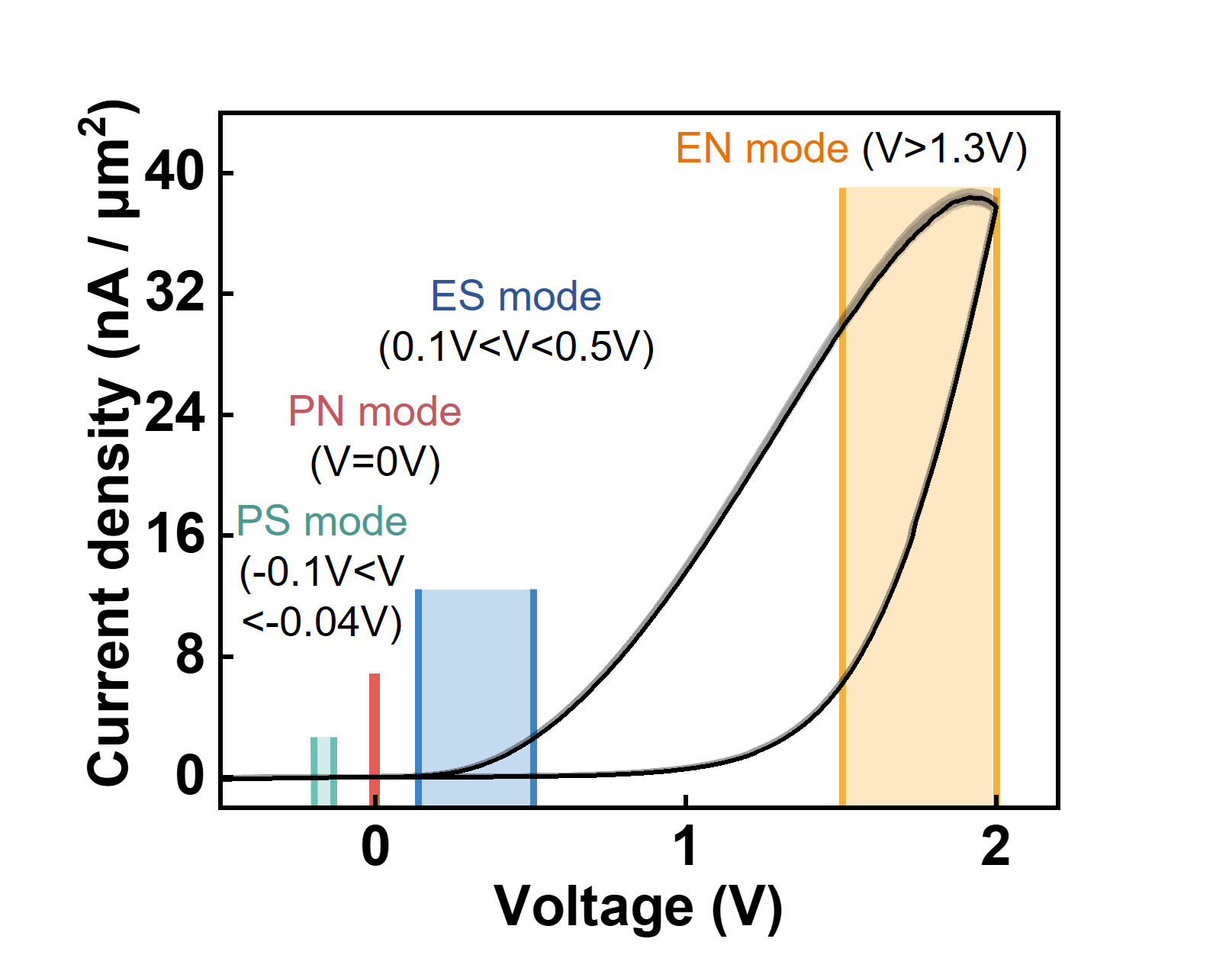


Supplementary Fig. 3 | Basic I-V characteristics of the device with a bidirectional DC sweeping voltage from 0 V to 2 V to 0 V to -2 V to 0 V with 50 cycles. Four modalities under different voltage regimes: PN mode at 0V, PS mode at -0.1 V to -0.04 V, ES mode at the range of 0.1-0.5 V and EN mode at a higher voltage range of >1.3V.


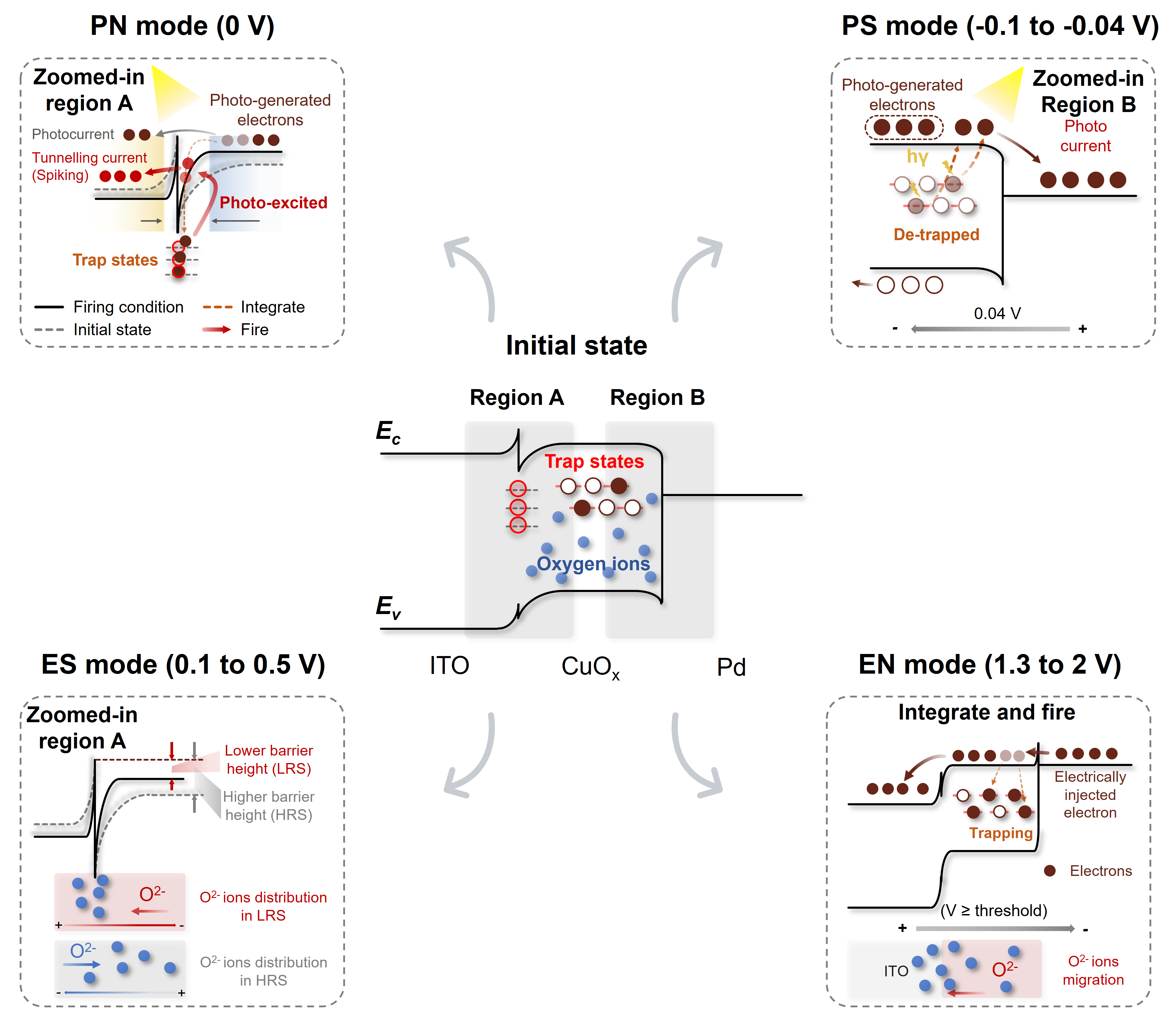


Supplementary Fig. 4 | Schematic illustration of the working mechanism of four modes. Middle: initial state of the device without bias and light illumination, with distributed interfacial and bulk trap states, and oxygen ions. Top left: PN mode photoresponsive LIF mechanism (0 V bias), indicating the integration and firing processes. Top right: PS mode photoresponse mechanism under the light illumination (-0.1 to -0.04 V bias). Bottom left: ES switching mechanisms, indicating the transition from HRS to LRS (0.1 to 0.5 V bias). Bottom right: EN mode electrical LIF behaviour mechanism, indicating the integration and fire process (1.3 - 2 V bias).


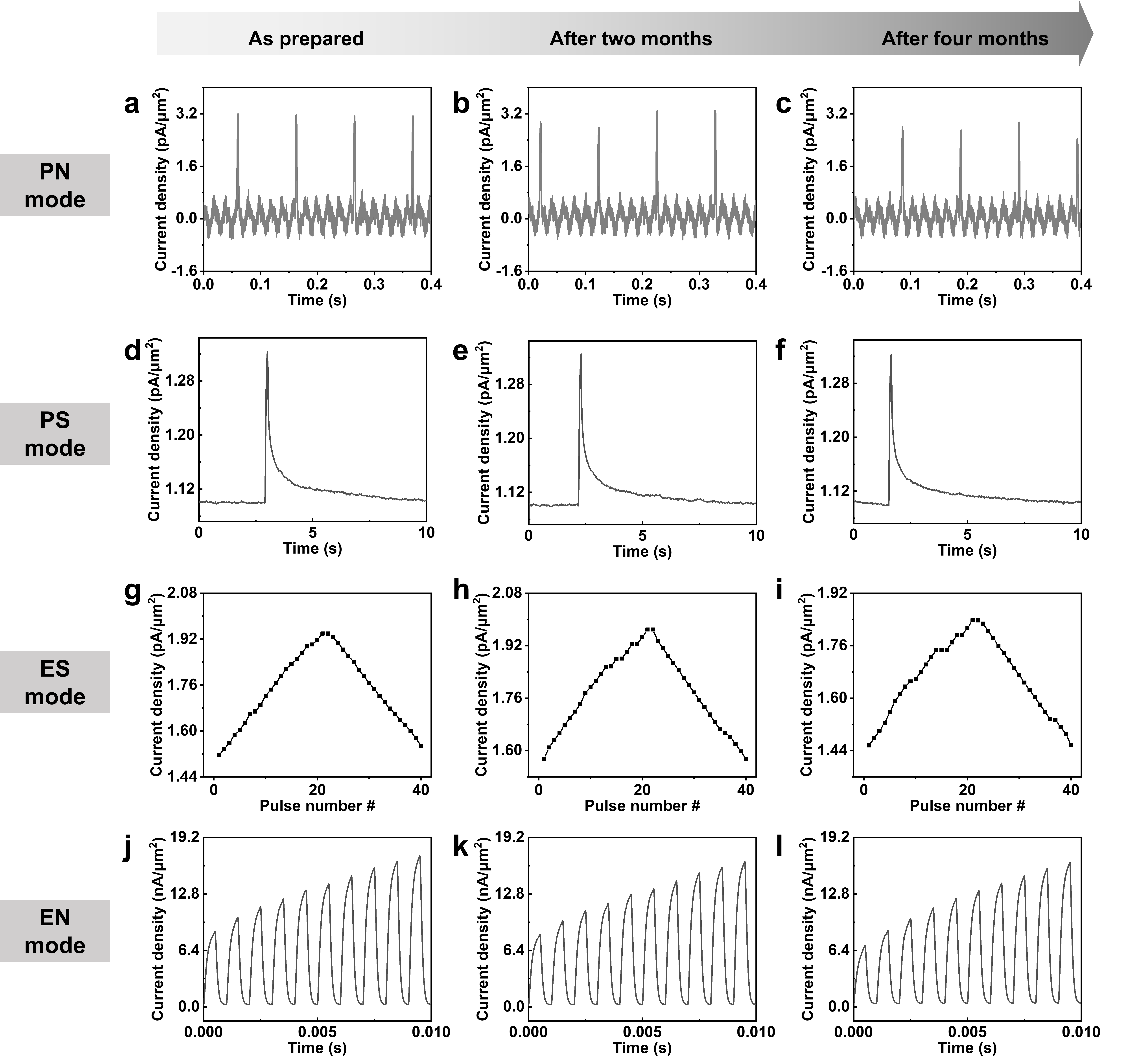


**Supplementary Fig. 5 |** **Time stability of the multi-paradigm device at different operating modes over four months.** (a-c) PN mode behaviours under 638 nm, 20 mW/cm^2^ light illumination, (d-f) PS mode triggered by 100 light pulses (638 nm, 120 mW/cm^2^, pulse width of 1 ms and interval of 1 ms) and a -40 mV read voltage, (g-i) LTP and LTD behaviours of ES mode, programmed by ±0.5 V voltage pulses (pulse width of 50 ms), and (j-l) EN mode behaviours triggered by 10 voltage pulses (1.5 V, pulse width of 0.5 ms and interval of 0.5 ms).


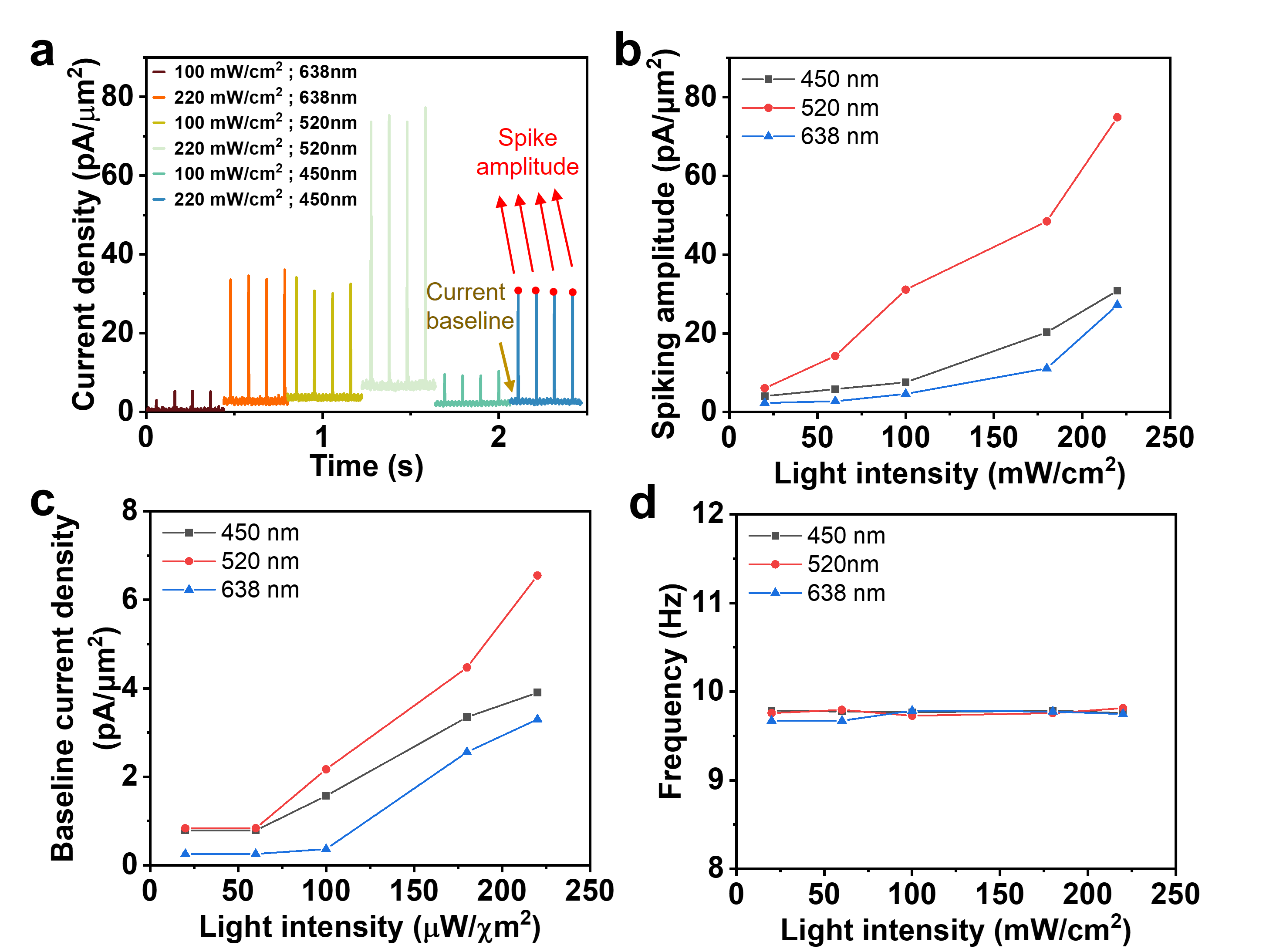


Supplementary Fig. 6 | Photoresponsive spiking characteristics of the multi-paradigm device in PN mode under different light wavelengths and intensities. Photoresponsive spiking characteristics under (a) 450 nm, 520 nm, and 638 nm light, each tested at different light intensities (100 mW/cm^2^ and 220 mW/cm^2^). Summary plots of (b) spiking amplitude and (c) current baseline density and (d) frequency as a function of light intensity.


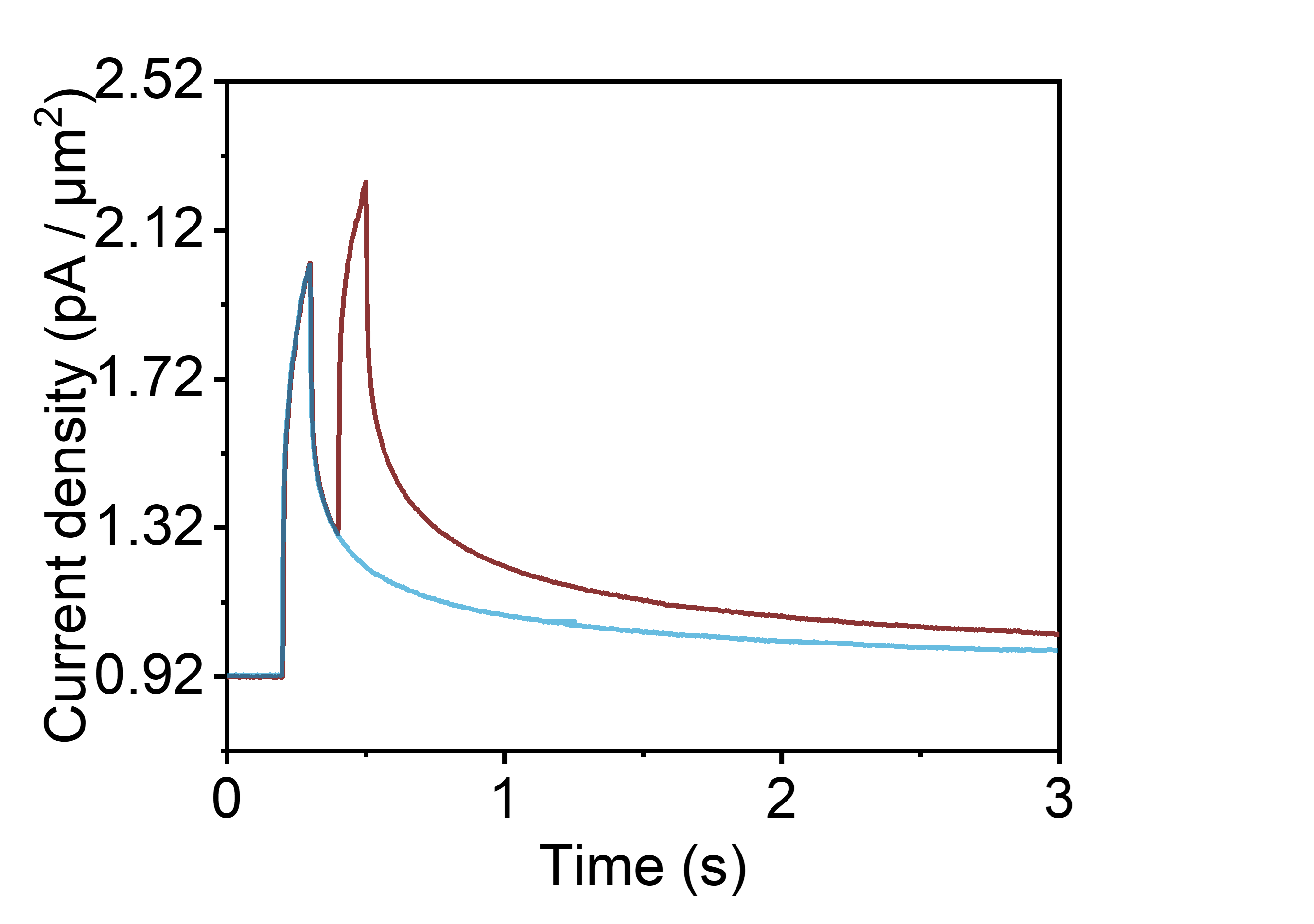


Supplementary Fig. 7 | Basic current response of multi-paradigm device in PS mode under single and multiple optical light pulses. The light intensity used is 1200 mW/cm^2^, and the pulse width is 100 ms. The wavelength is 520 nm. The read voltage is -40 mV.


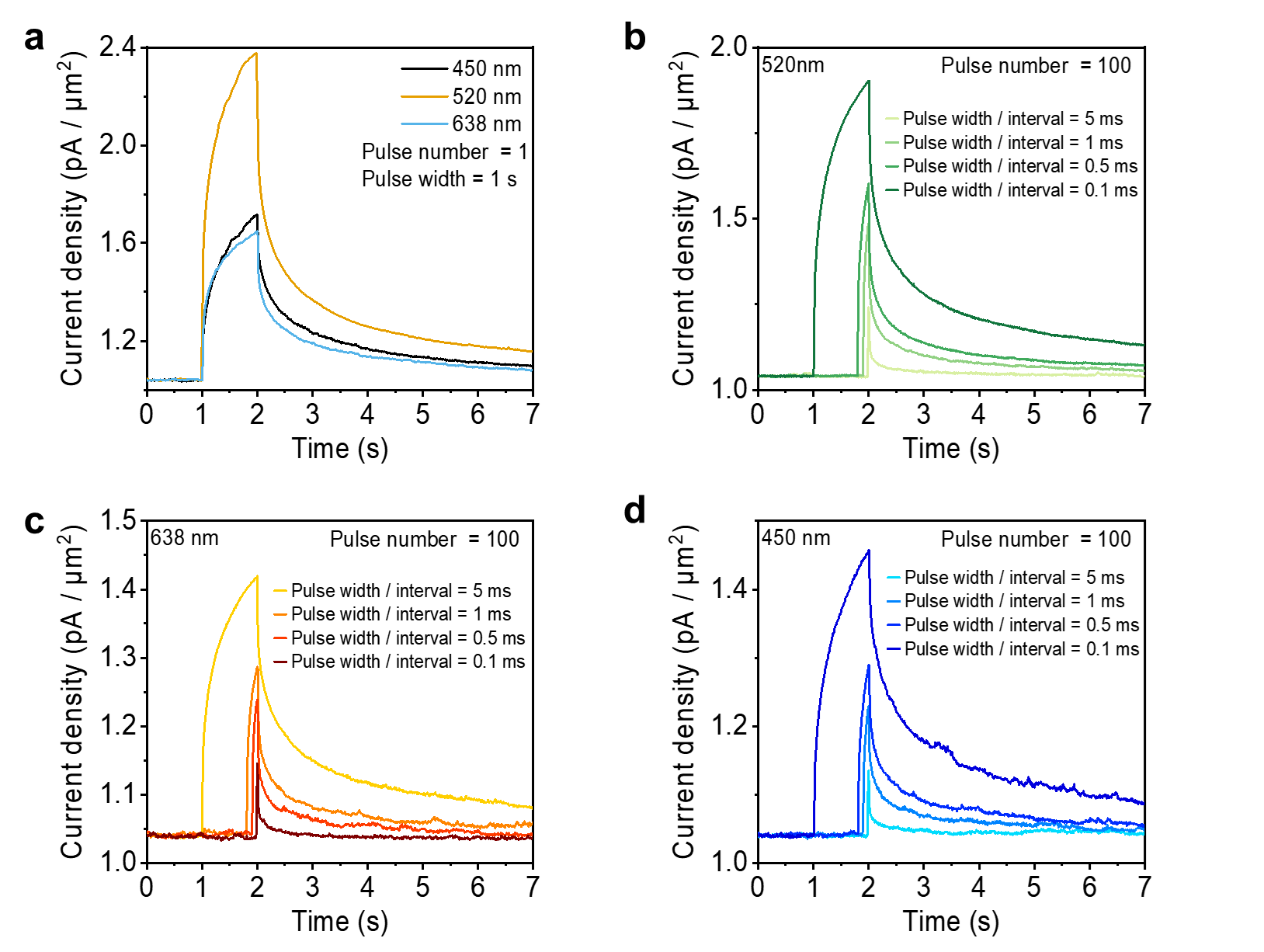


Supplementary Fig. 8 | Light wavelength, pulse width and pulse interval-dependent PS mode characteristics. (a) Current responses under different input wavelengths (450 nm, 520 nm, and 638 nm) with the same pulse width (1 s), light intensity 120 mW/cm^2^. (b) Current responses under 520 nm illumination with varying pulse widths and intervals, light intensity 120 mW/cm^2^. (c) Current responses under 638 nm illumination with varying pulse widths and intervals, light intensity 120 mW/cm^2^. (d) Current responses under 450 nm illumination with varying pulse widths and intervals, light intensity 120 mW/cm^2^. All measurements are performed under a read voltage of -40 mV.


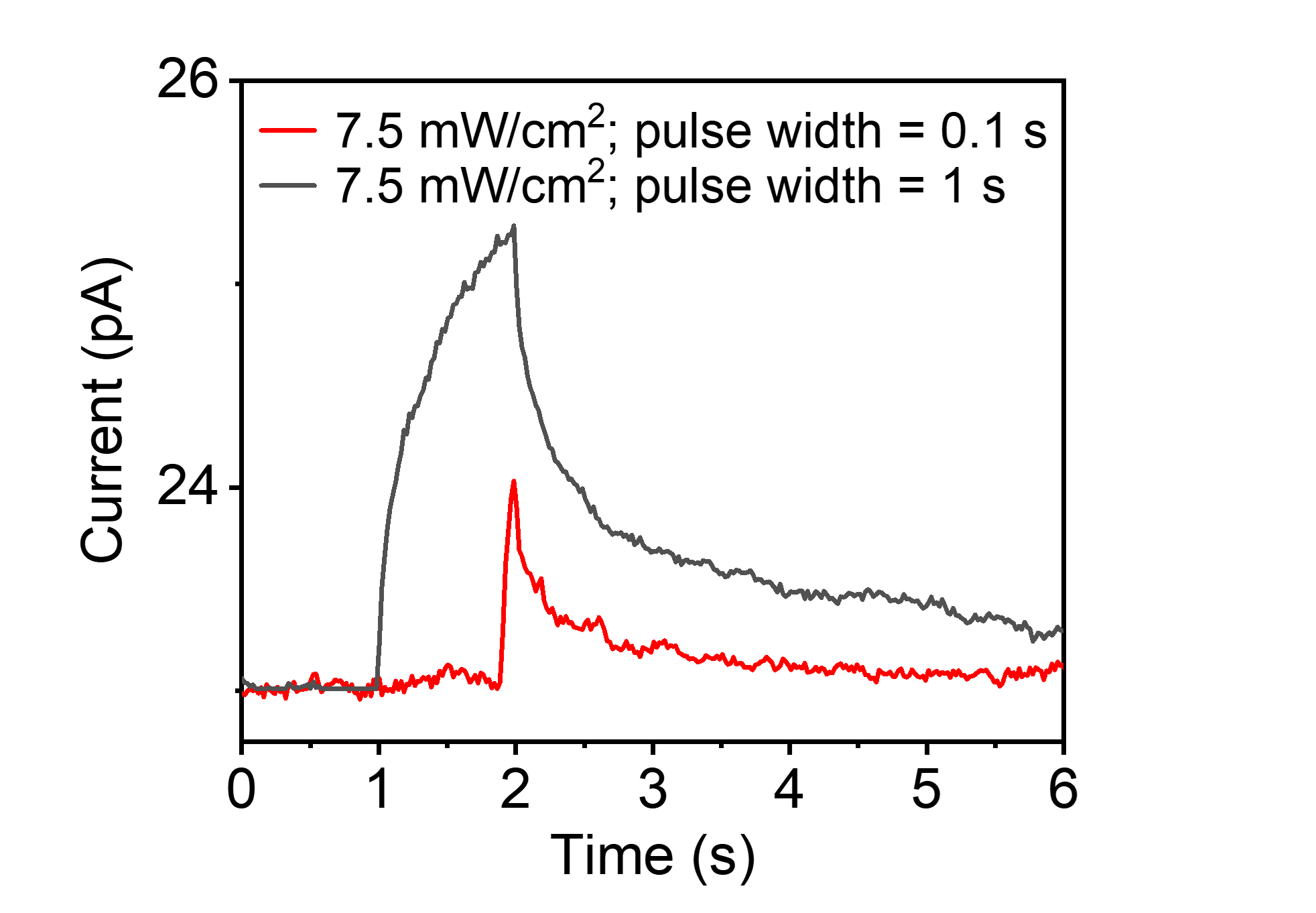


Supplementary Fig. 9 | Device photoresponse in PS mode under 520 nm with an intensity of 7.5 mW/cm^2^ with pulse frequency at 1 Hz and 10 Hz (50% duty cycle). The read voltage is -40 mV.

***
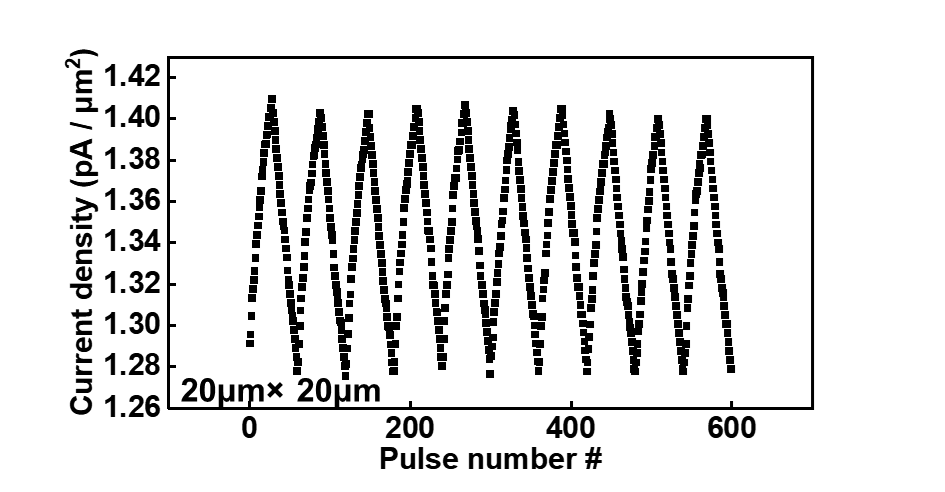
***

Supplementary Fig. 10 | Cyclic LTP and LTD of device in ES mode. The device is programmed by 30 positive pulses (+ 0.5 V/100 ms) and 30 negative pulses (- 0.5 V/100 ms) for 10 cycles with a read voltage of 0.01 V.

***
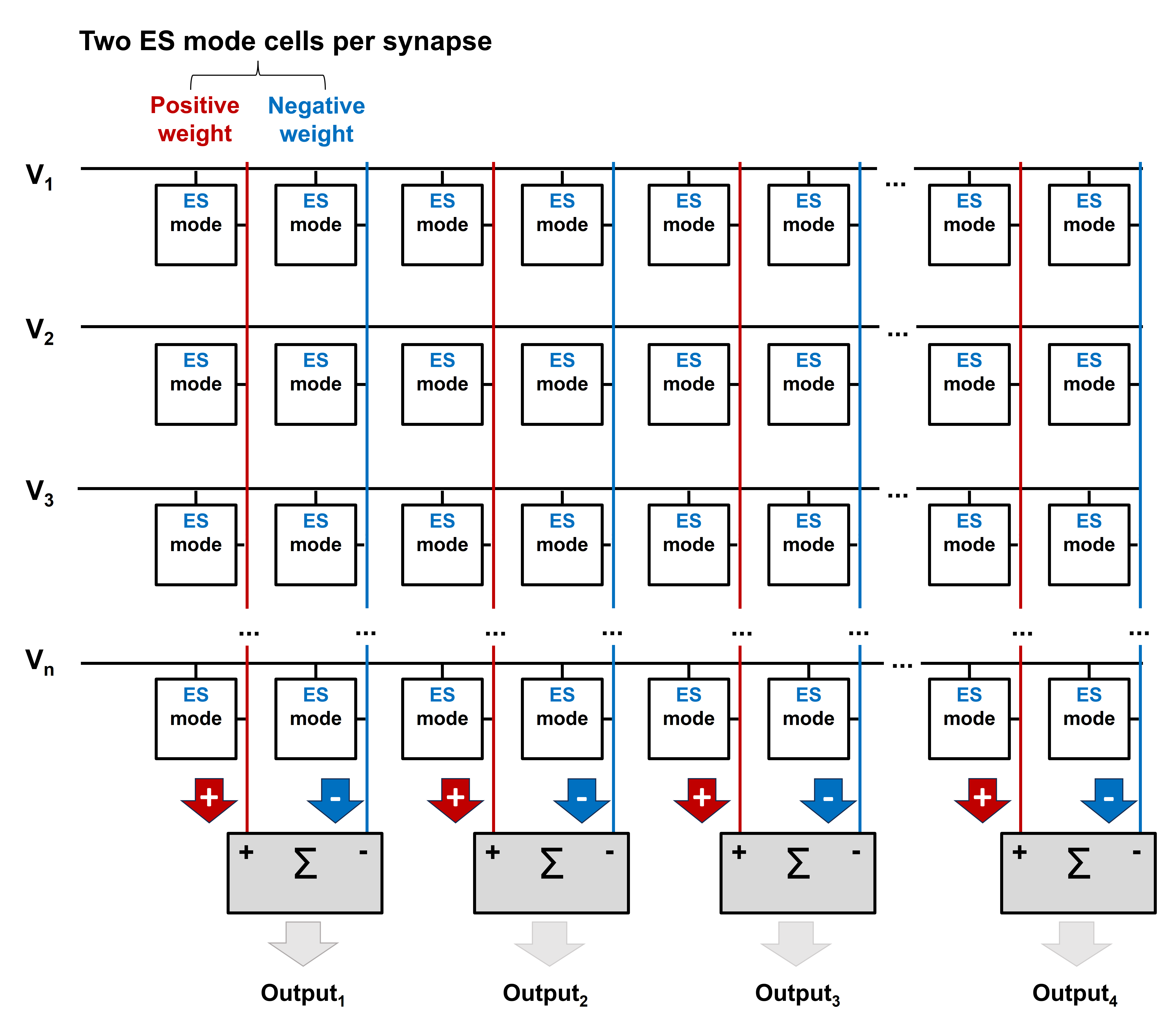
***

Supplementary Fig. 11 | ES mode cell array architecture for signed weights representation.


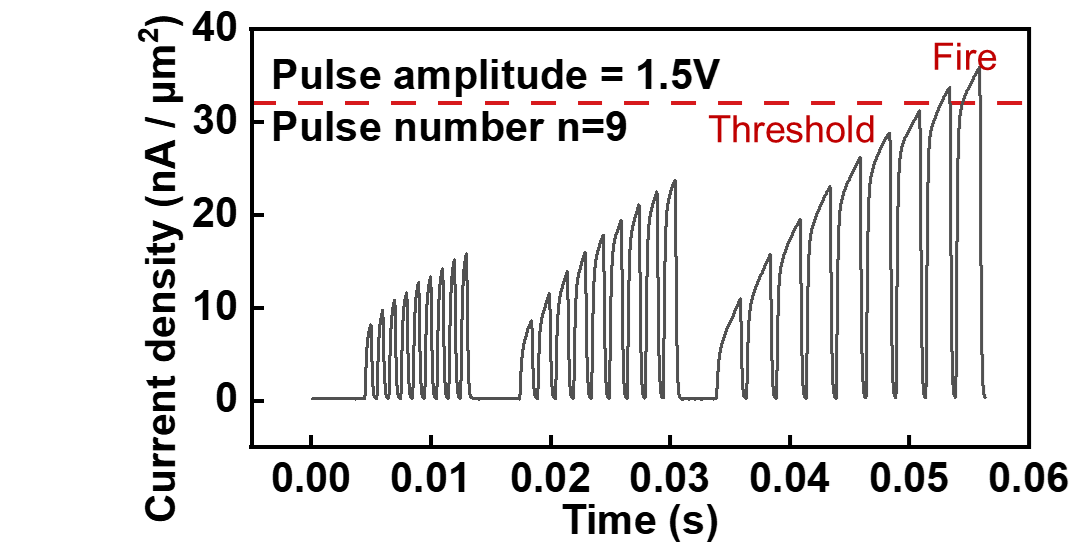


Supplementary Fig. 12 | EN mode characteristics in the multi-paradigm device at different electrical pulse widths and intervals (500 µs/500 µs, 1000 µs/500 µs, and 2000 µs/500 µs) with a pulse number of 9.


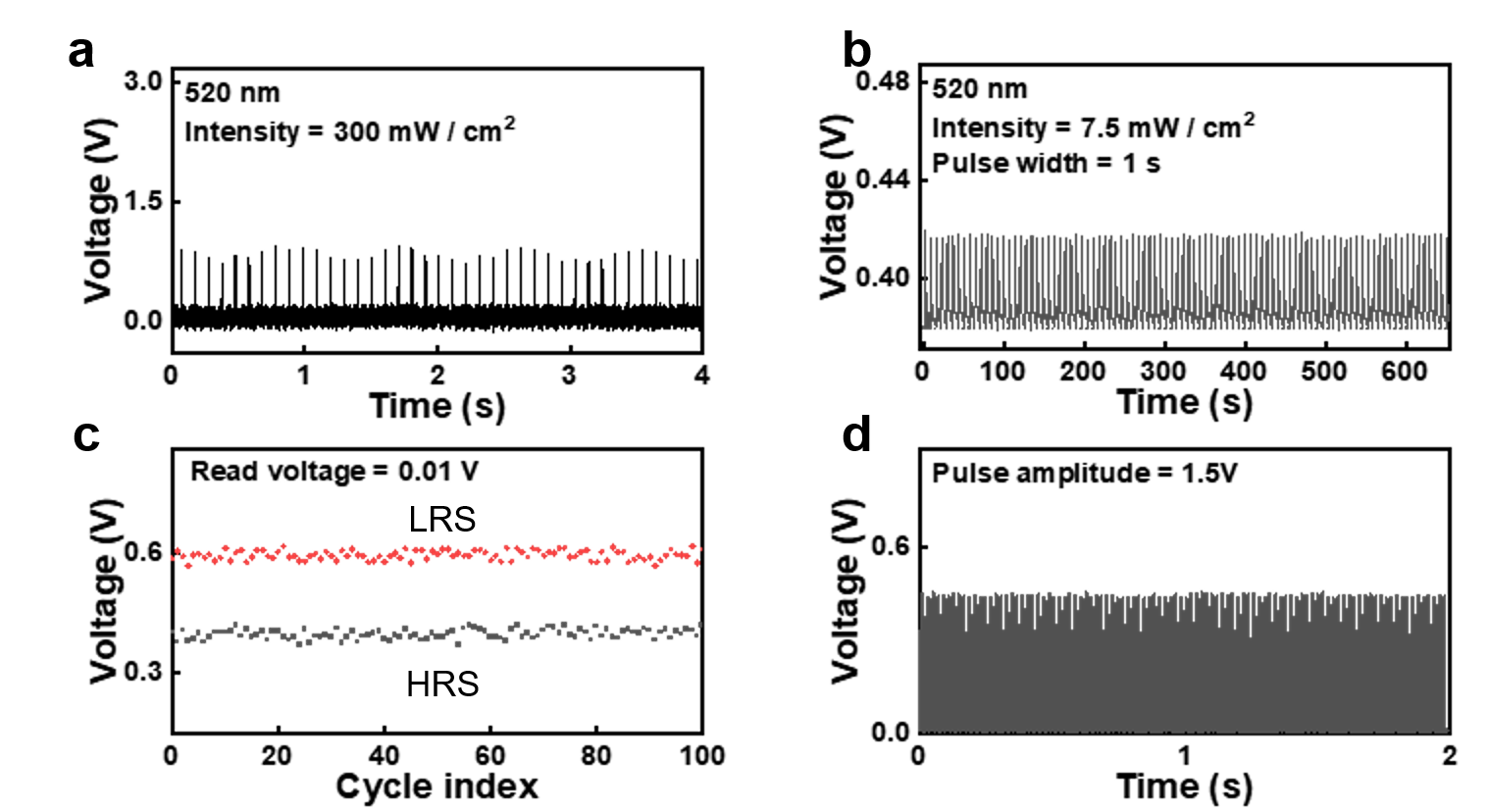


**Supplementary Fig.** **13 | Cycle-to-cycle performance of the multi-paradigm cell.** The devices in the array are read by the board-level dynamic control circuit in our prototype, outputting voltage signals. (a) Cyclic optical LIF spiking of PN mode under 520 nm illumination (300 mW/cm^2^). (b) Cyclic photoresponse of PS mode cell triggered by 450 nm light pulses (7.5 mW/cm^2^, pulse width of 1 s), with a read voltage of -40 mV. (c) Cyclic HRS and LRS behaviour of ES mode cell, programmed by 0 to ±0.5 V voltage sweeps and read at 0.01 V. (d) Cyclic voltage response of EN mode cell under 10 voltage pulses (1.5 V, 0.5 ms width and interval) for each cycle.


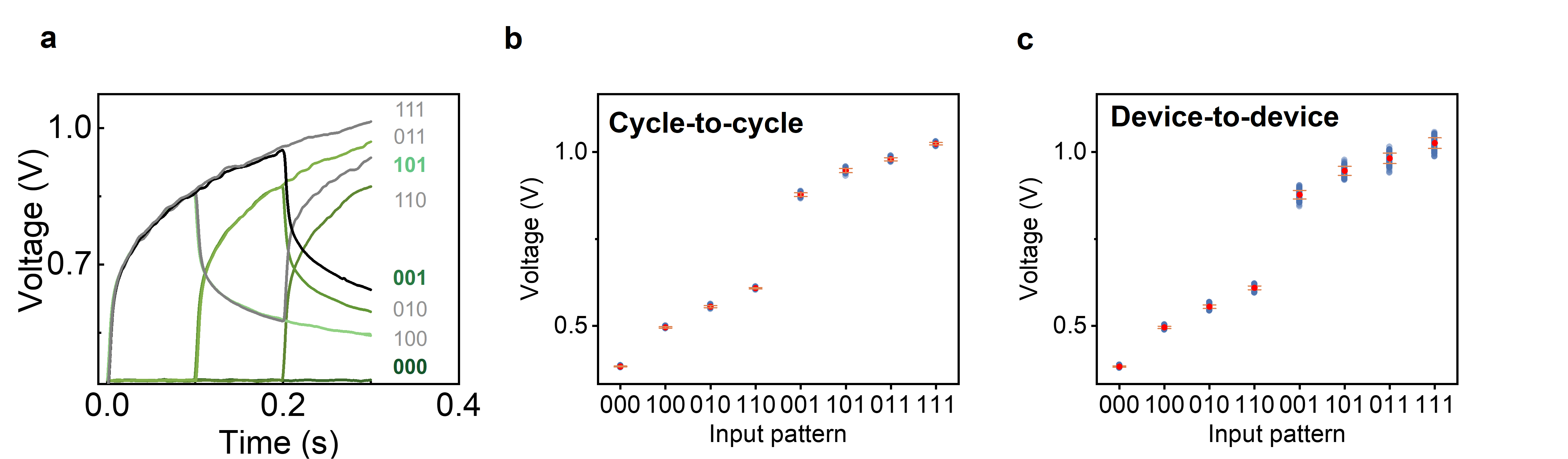


**Supplementary Fig.** **14 | Cycle-to-cycle and device-to-device variations for data encoding.** (a) 3-bit data encoding in the cell. The cell was tested with input optical patterns ranging from 000 to 111, where ‘1’ corresponds to a 100 ms, 520 nm light pulse at 1200 mW/cm^2^ and ‘0’ corresponds to a light pulse of 0 mW/cm^2^. (b) Cycle-to-cycle variation of a single device under repeated encoding operations. Blue scatter points represent the encoded voltages obtained from 50 repeated cycles. The red dot indicates the mean value, and the orange line shows the error bar. (b) Device-to-device variation across the 12×12 array. Each blue scatter point represents the encoding result of an individual device. The red dot indicates the mean value, and the orange line shows the error bar.


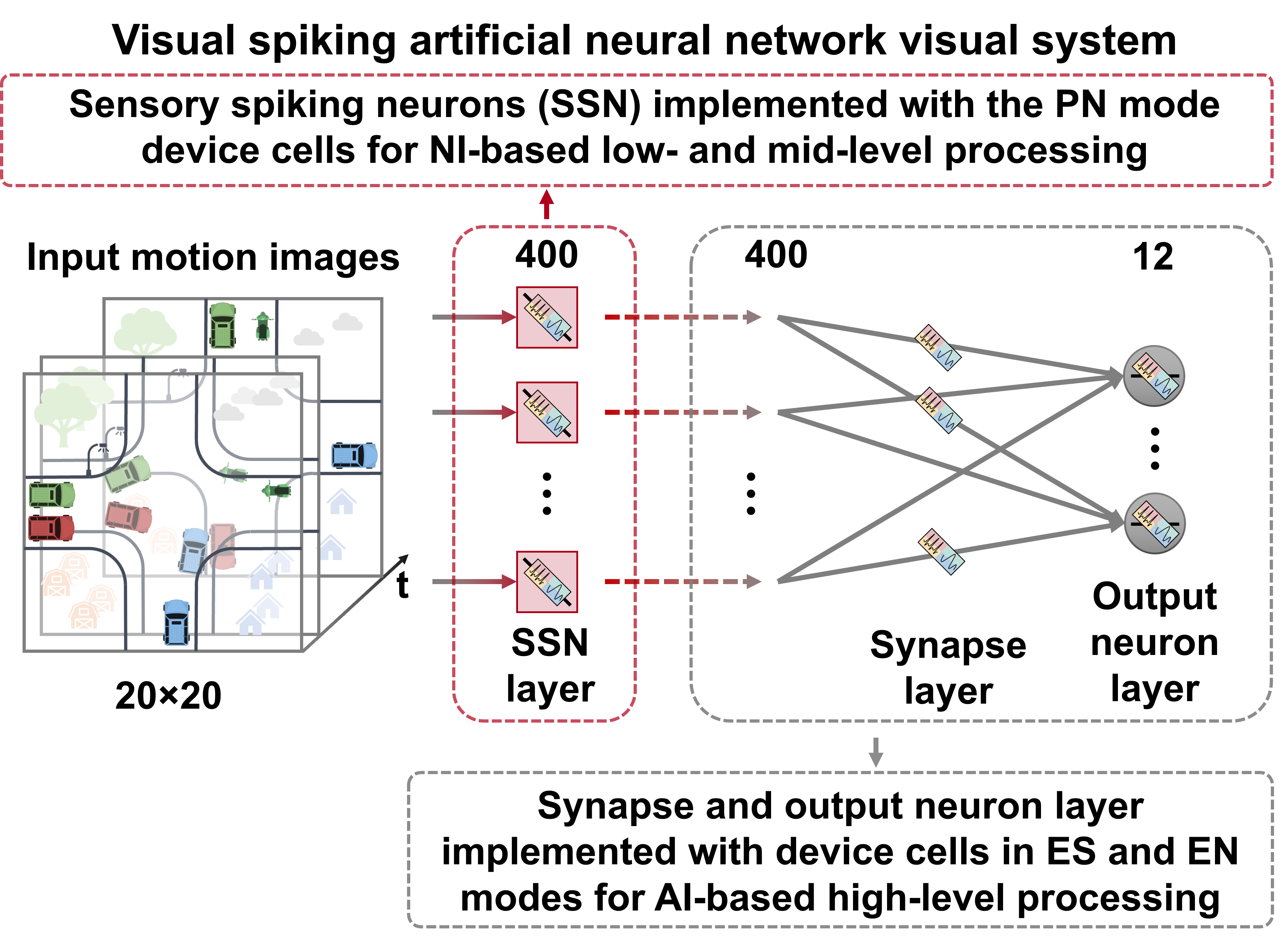


Supplementary Fig. 15 | Network structure and hardware implementation of the visual spiking artificial neural network system. The visual spiking artificial neural network has 400 input sensory spiking neurons corresponding to the 20 × 20 PN mode device cell array, and 12 output neurons realized by the EN mode device cell array. The synapses that connect the input and output layers are implemented by the ES mode cell array.


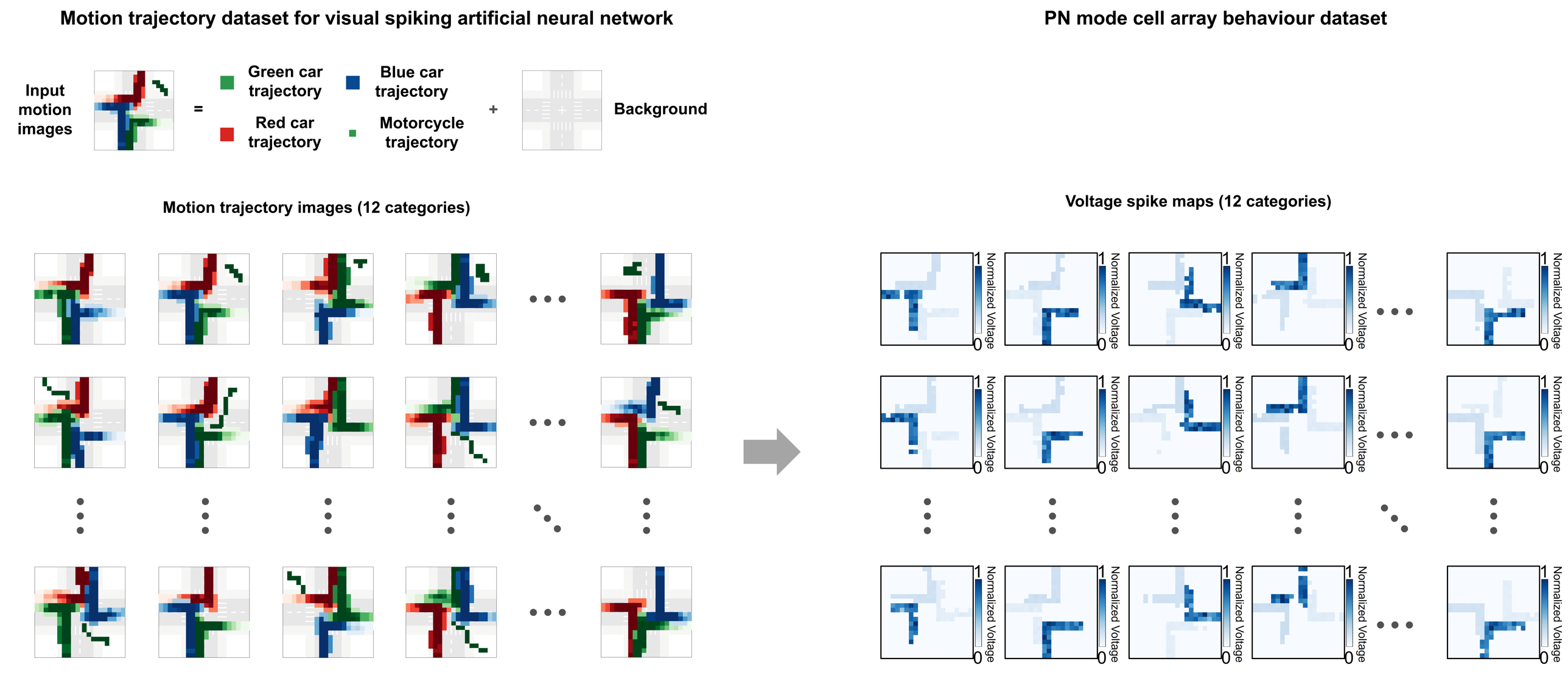


Supplementary Fig. 16 | Visual spiking artificial neural network dataset. The motion trajectory dataset consists of 12 different categories of colour-mixed motion images, each comprising 20 consecutive motion steps. The size of each motion image is 20 × 20 pixels. Similarly, the PN mode cell array behaviour dataset consists of 12 different categories of spike map patterns, each with the same resolution of 20 × 20 pixels, containing 20 sequential spikes. 480 of these spike maps were used for training the AI section in this configuration, and 120 spike maps were used for testing the AI section of this configuration.


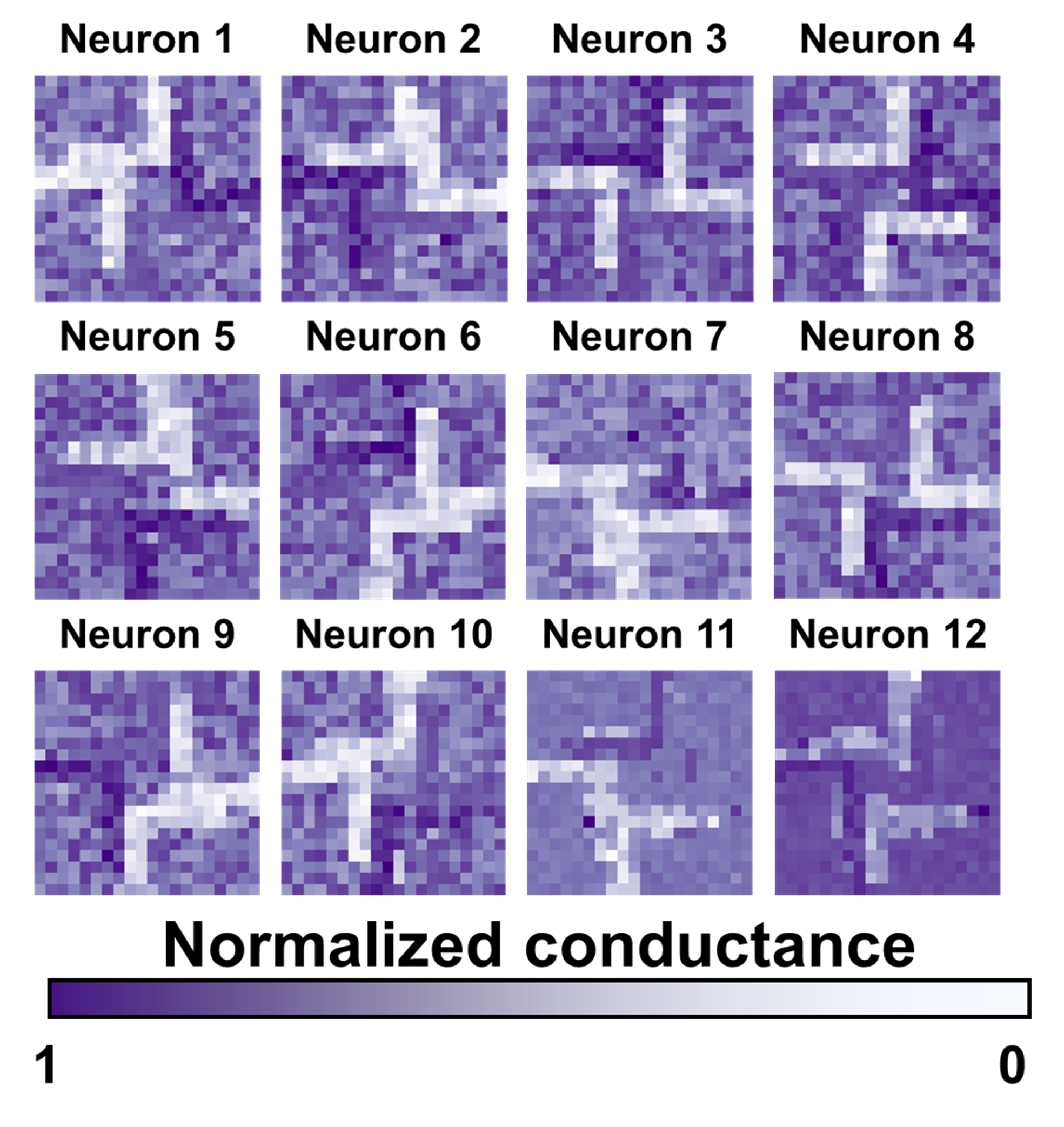


Supplementary Fig. 17 | Weight maps of visual spiking artificial neural network synapse layer.


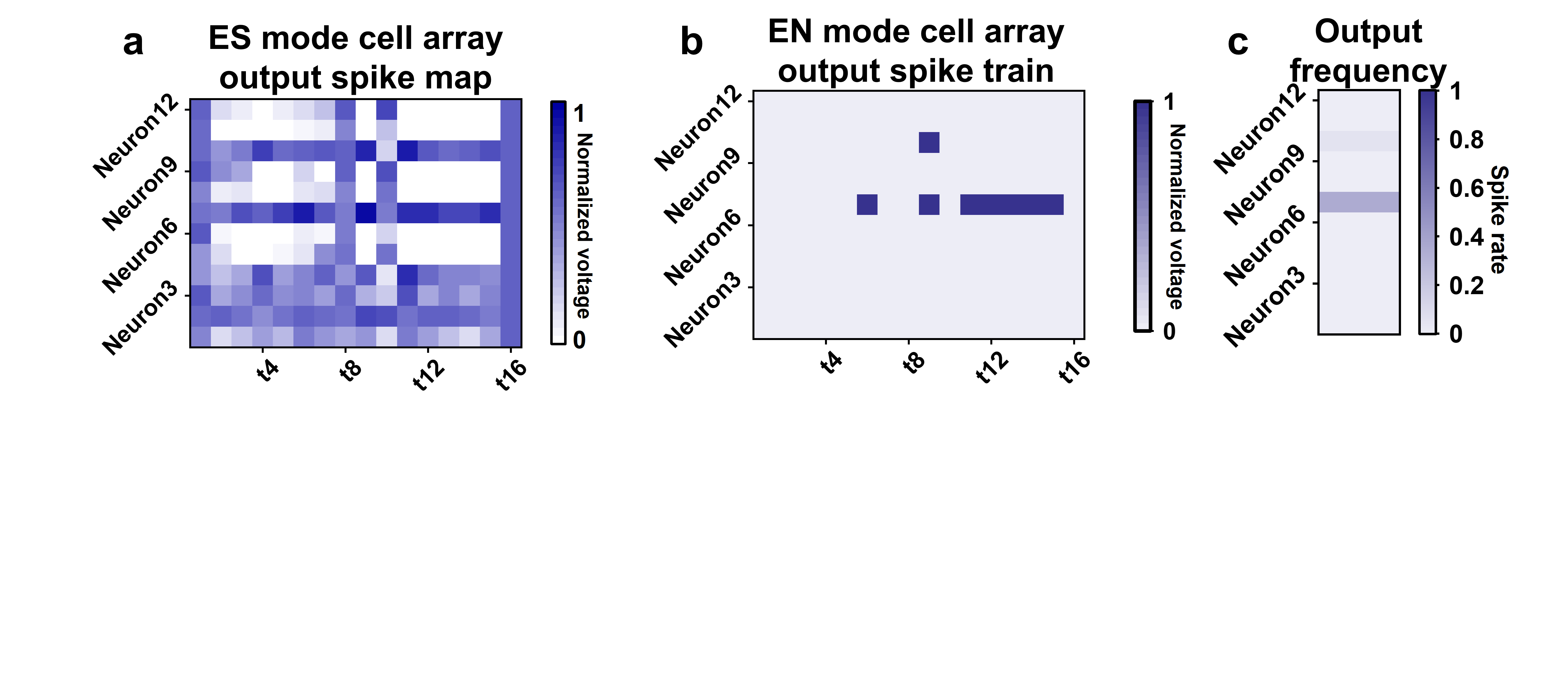


Supplementary Fig. 18 | Spike maps from ES and EN mode cell array. (a) The output voltage spike maps from the ES mode cell array and (b) the output spike trains from the EN mode cell array and the corresponding (c) output frequency, generated using an image from the test dataset labelled as class 5.





Supplementary Fig. 19 | Network structure of visual spiking recurrent neural network. The input sensory spiking neuron layer of visual spiking recurrent neural network configuration comprises 441 sensory spiking neurons implemented by the PN mode cell array. The recurrent layer is composed of 32 recurrent neurons, 441 × 32 forward recurrent synapses, and 32 × 32 backward recurrent synapses. The recurrent neurons and recurrent synapses are implemented by the EN mode cell array and ES mode cell array, respectively. The final fully connected layer consists of 8 output neurons implemented with the EN mode cell array and 256 (32 × 8) synapses implemented by the ES mode cell array.


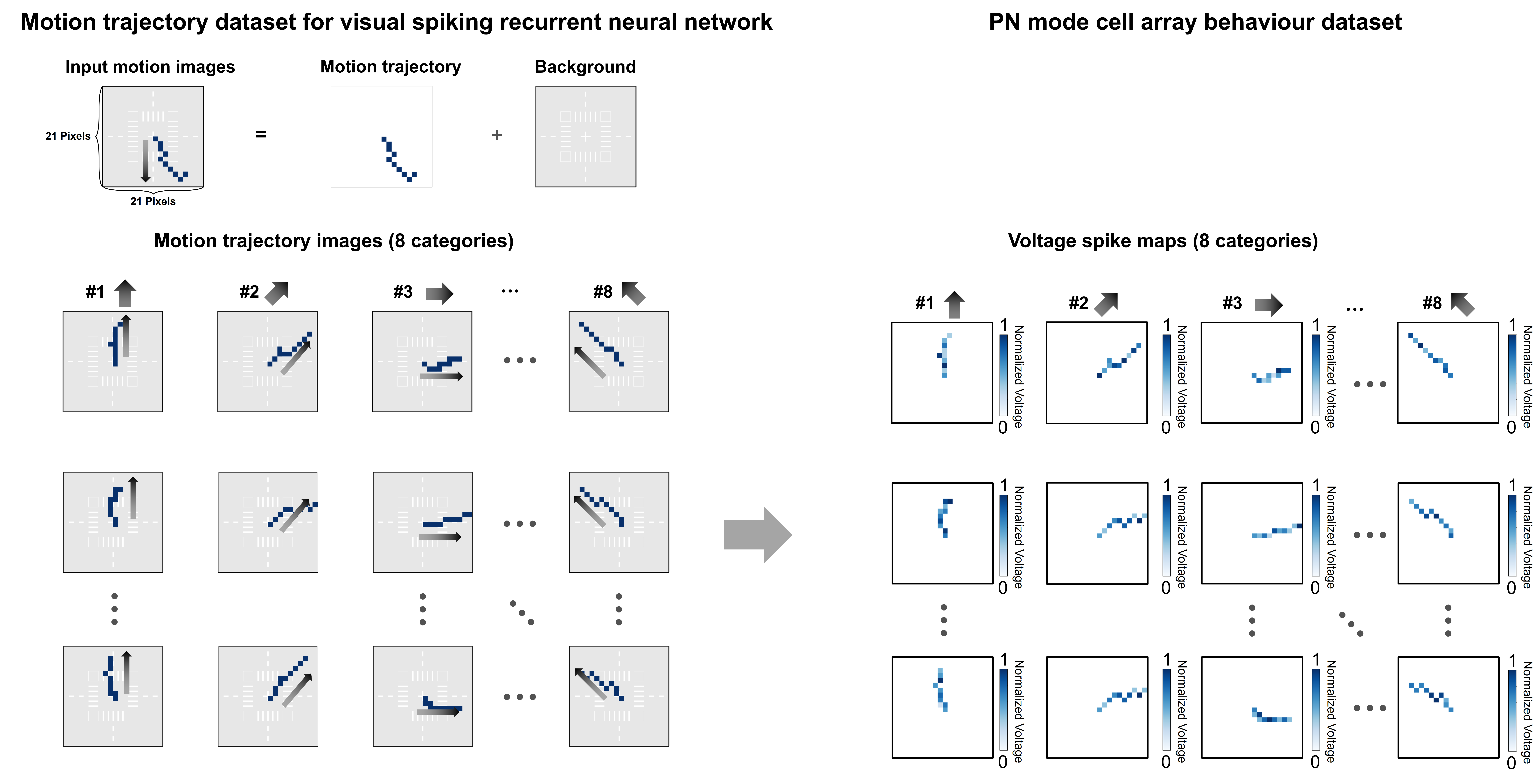


Supplementary Fig. 20 | Visual spiking recurrent neural network datasets. The motion trajectory dataset contains 1,640 distinct motion images (t_0_ to t_10_) with 8 possible moving directions. Each motion image (21 × 21) contains 10 sequential vehicle motion steps. Similarly, the simulated PN mode cell array behaviour dataset consists of 8 possible spike maps (21 × 21) containing 10 sequential spikes. 1,312 of these spike maps were used for training the AI section in this configuration, and the rest of the 328 spike maps were used for testing the AI section of this configuration.


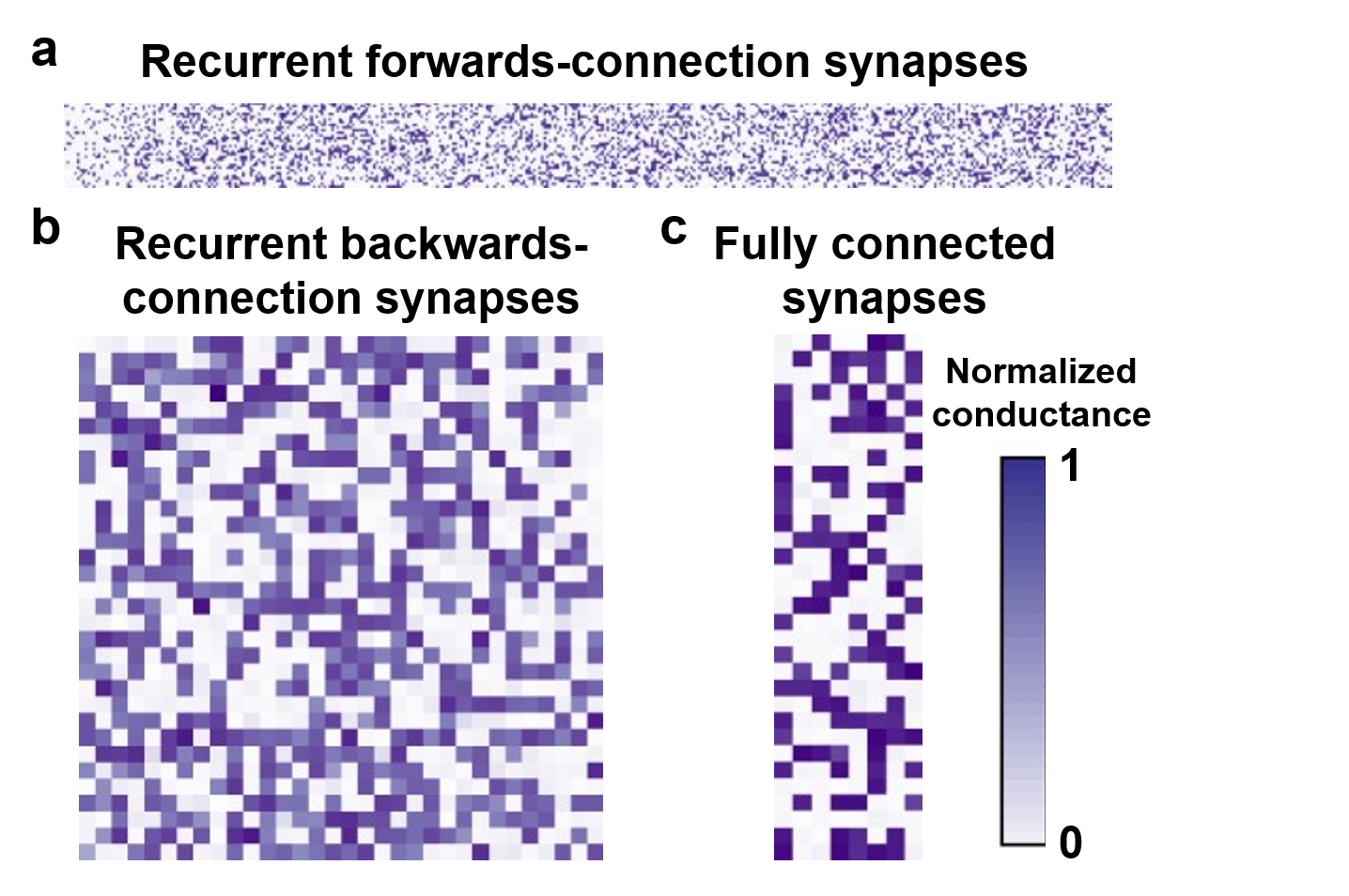


Supplementary Fig. 21 | Weight maps of visual spiking recurrent neural network. (a) recurrent forwards-connection synapses, (b) recurrent backwards-connection synapses and (c) fully connected synapses.


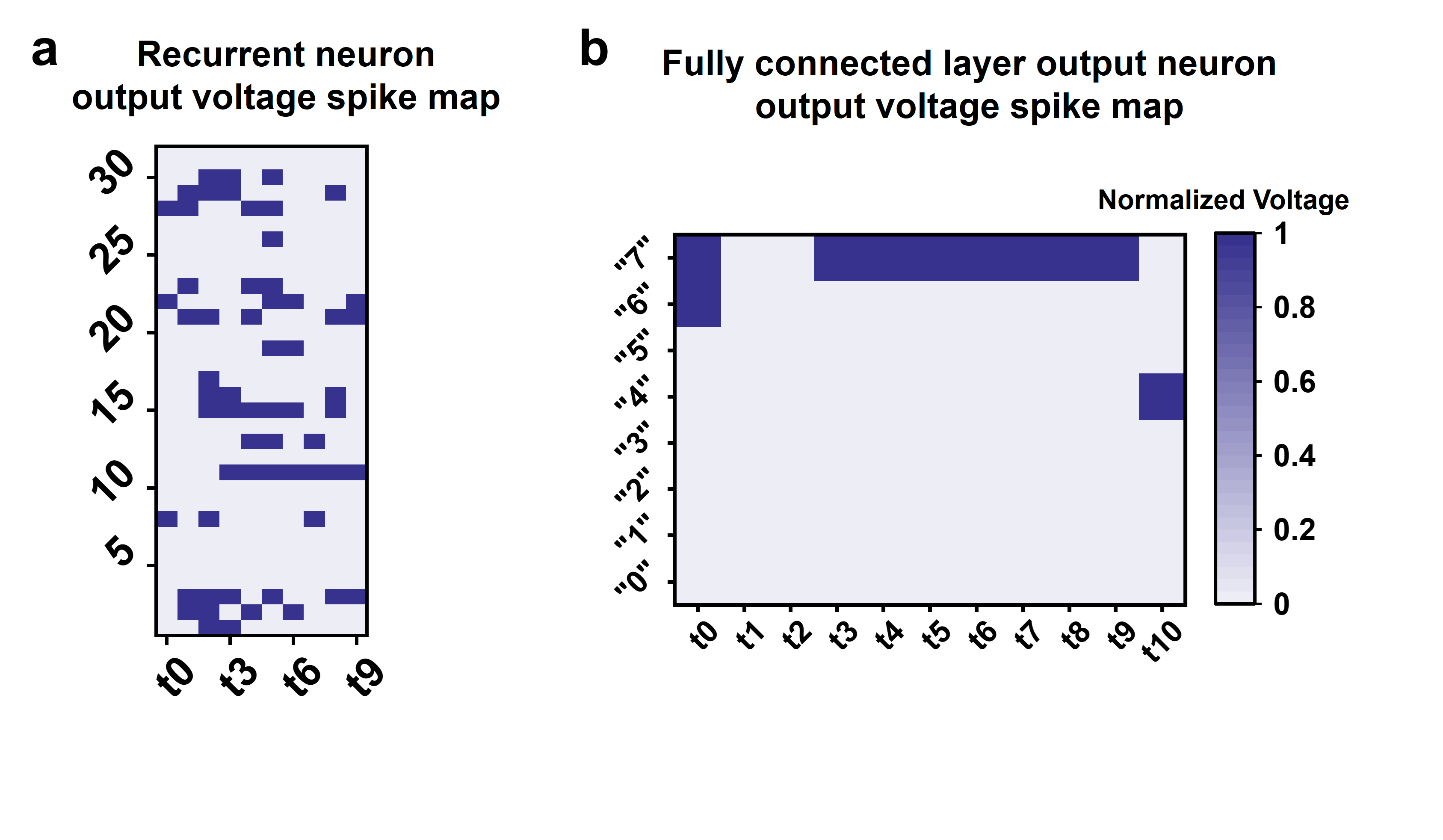


Supplementary Fig. 22 | Spike maps of visual spiking recurrent neural network configuration. (a) The output voltage spike maps of the recurrent neuron and (b) the output voltage spike map of the fully connected layer output neuron generated using an image from the test dataset labelled as class 7.


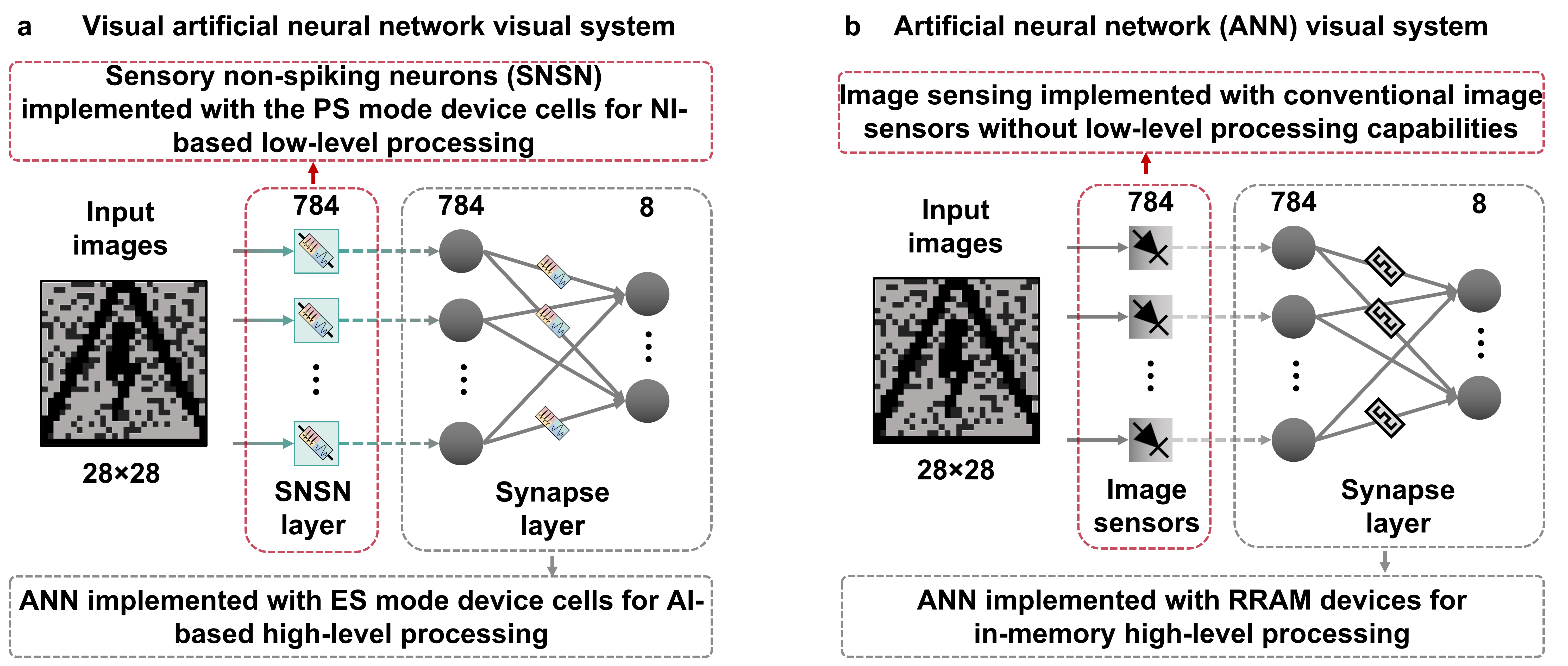


Supplementary Fig. 23 | Network structure and implementations of visual artificial neural network. The visual artificial neural network consists of an input sensory non-spiking neuron layer (784 neurons) and a synapse layer with 784 × 8 connections. Two different implementations of the visual artificial neural network-based visual system and a traditional artificial neural network (ANN) visual system are demonstrated: one with multi-paradigm devices and one without. (a) visual artificial neural network implemented with PS mode device cells for the sensory non-spiking neuron layer and ES mode device cells for the synapse layer. (b) Traditional ANN implemented with conventional image sensors for the input layer and electrical resistive random-access memory (RRAM) devices for the synapse layer.


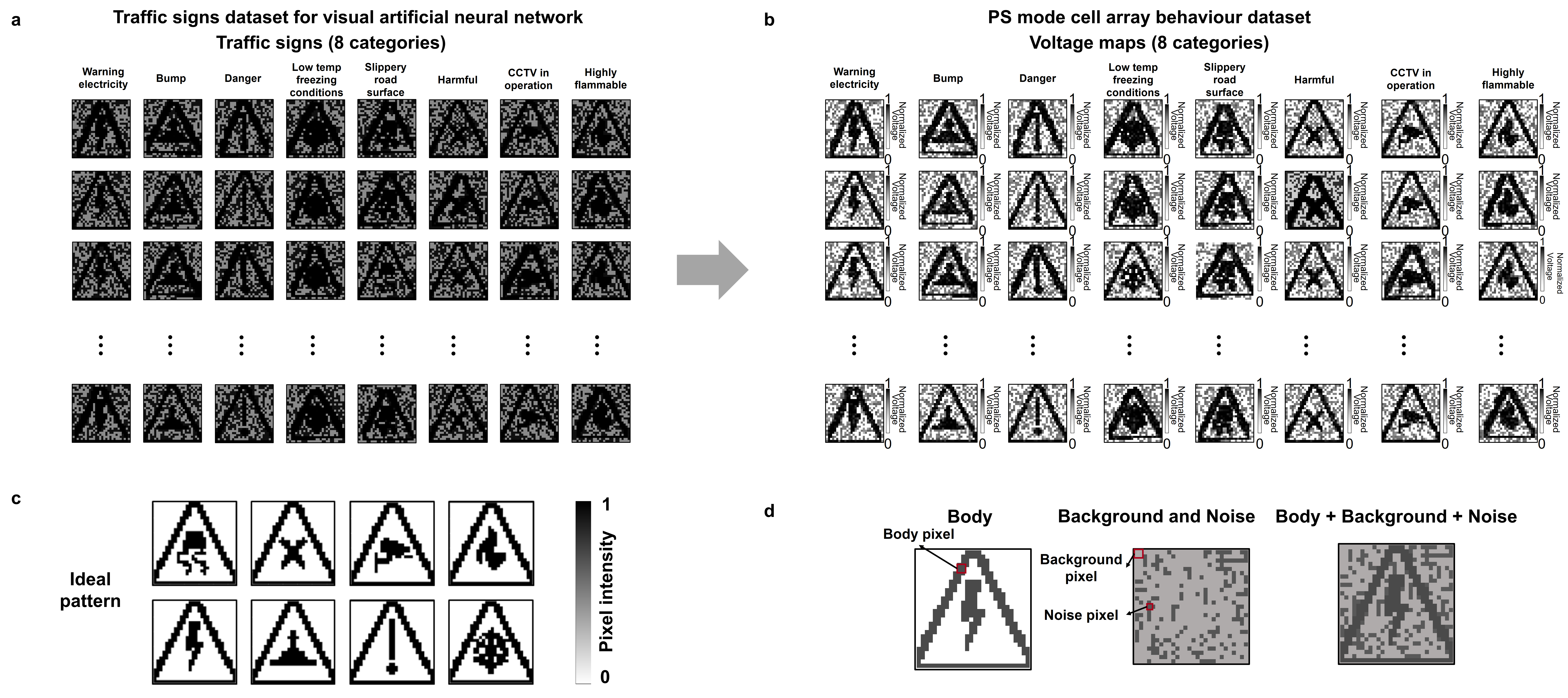


Supplementary Fig. 24 | Visual artificial neural network datasets. (a) The traffic sign image dataset includes 8 categories of 28 × 28 noisy traffic sign images (warning electricity, bump, danger, low temperature/freezing conditions, slippery road surface, harmful, CCTV in operation, and highly flammable). (b) The simulated PS mode cell array behaviour dataset consists of 8 possible voltage map patterns, each with a resolution of 28 × 28 pixels. In the dataset, 192 samples are used for training, 39 for validation, and 48 for testing. (c) Ideal patterns for the 8 traffic sign image categories. (d) Example of a noisy traffic sign image.


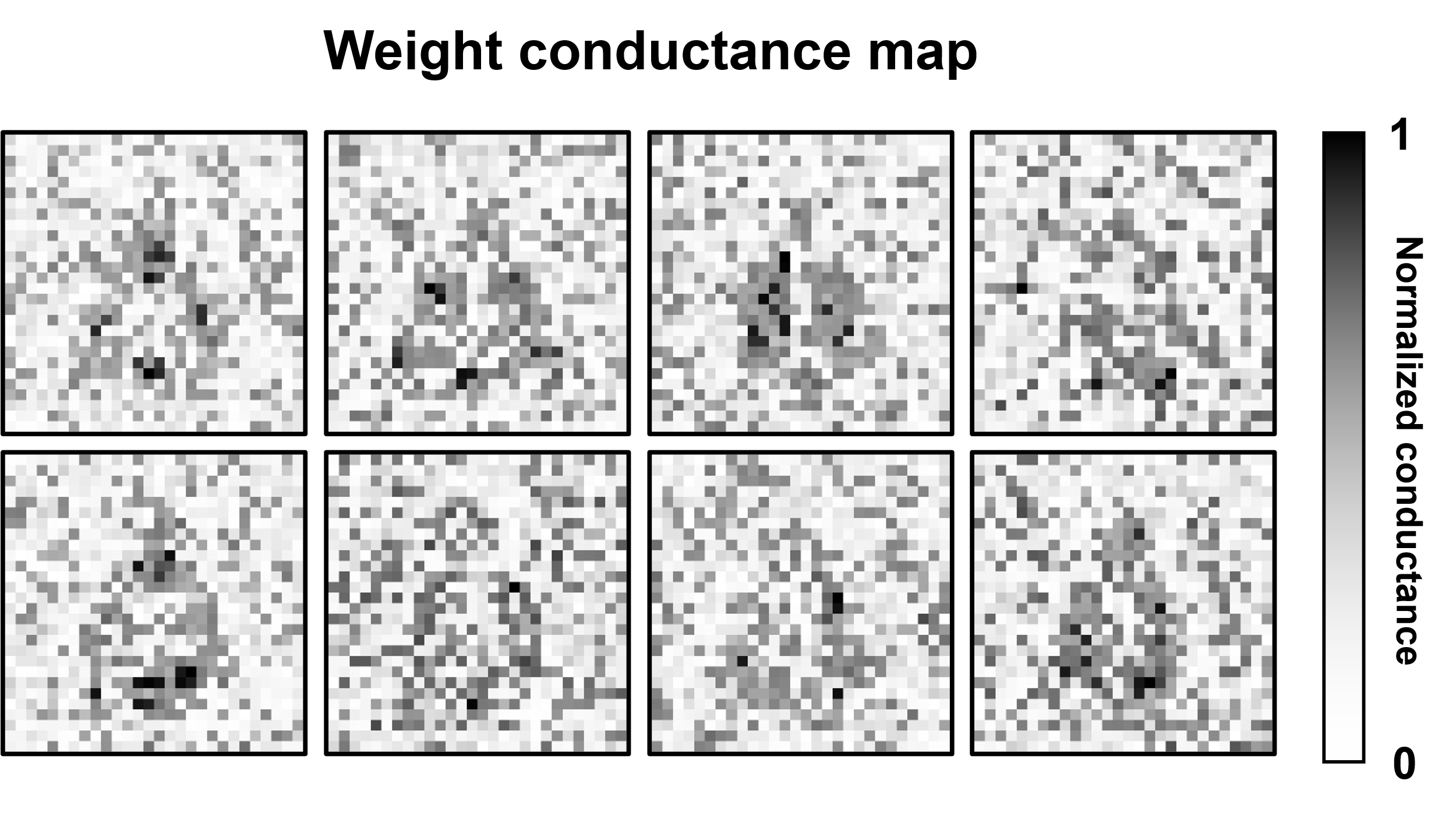


Supplementary Fig. 25 | Weight maps of visual artificial neural network synapse layer.


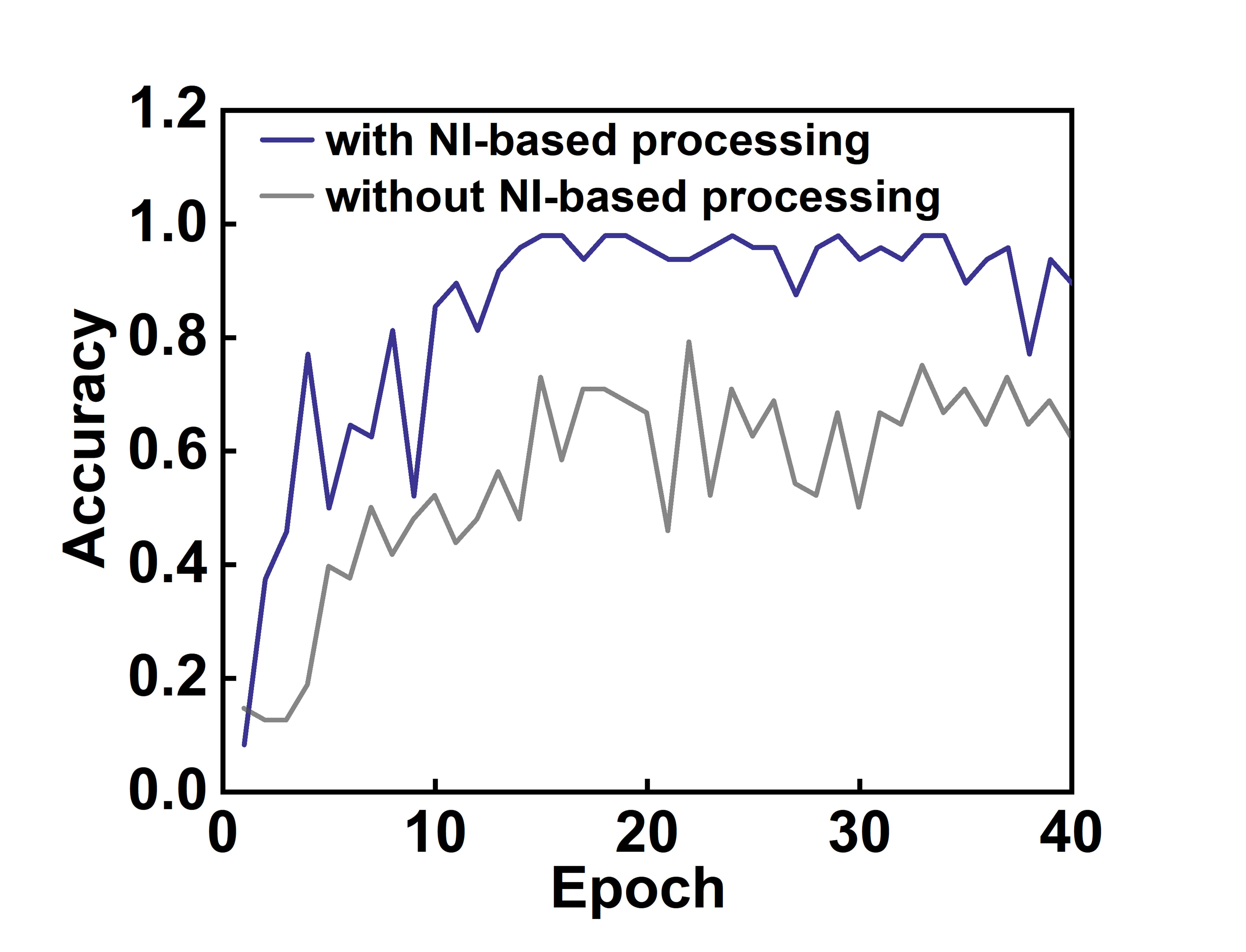


Supplementary Fig. 26 | Comparison of classification accuracy of visual artificial neural network-based system with and without NI-based low-level processing. The system with NI-based processing achieves higher classification accuracy of 97% when recognizing noisy traffic sign images, representing a > 10 % improvement compared to the system without non-spiking NI-based low-level processing functions.


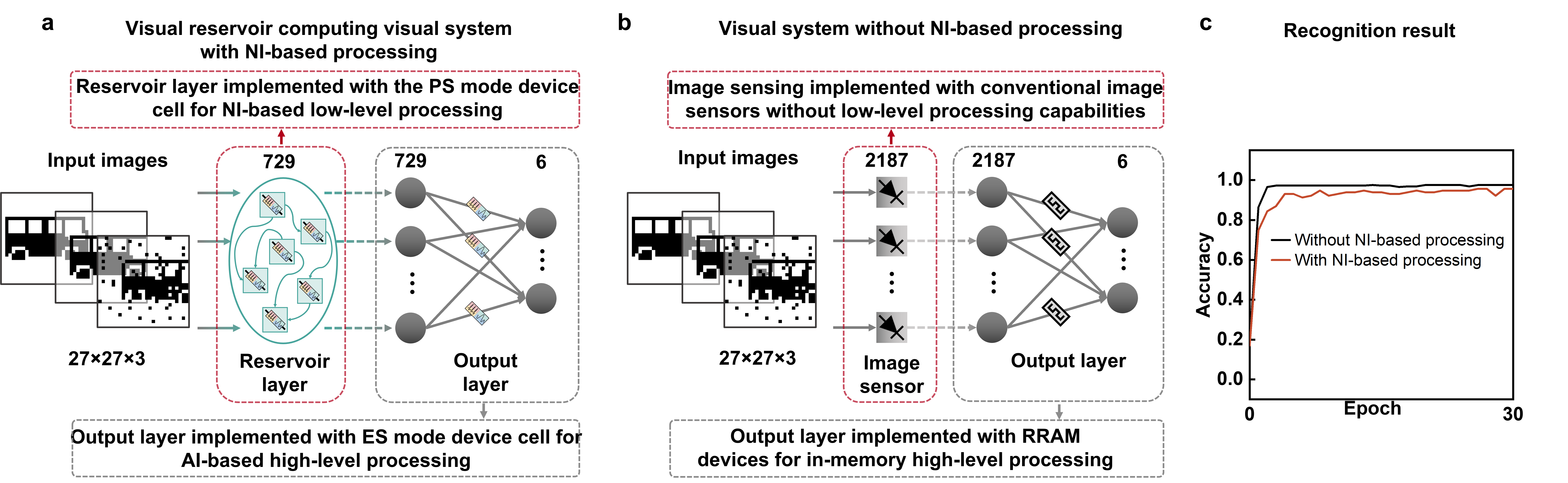


Supplementary Fig. 27 | Comparison of the network structures and recognition accuracy of (a) Visual reservoir computing-based visual system with NI-based processing fully implemented with multi-paradigm devices and (b) a visual system without NI-based processing implemented with conventional image sensors and electrical RRAMs. (c) Recognition accuracy of (a) and (b) on the same traffic sign recognition task. The accuracy is 95.8% for the visual reservoir computing-based visual system and 97.5% for the visual system without NI-based processing. The visual reservoir computing-based visual system has a smaller AI network size of 4374 synapses (729 × 6) compared to 13122 synapses (2187 × 6) for the visual system without NI-based processing, while achieving comparable recognition accuracy.


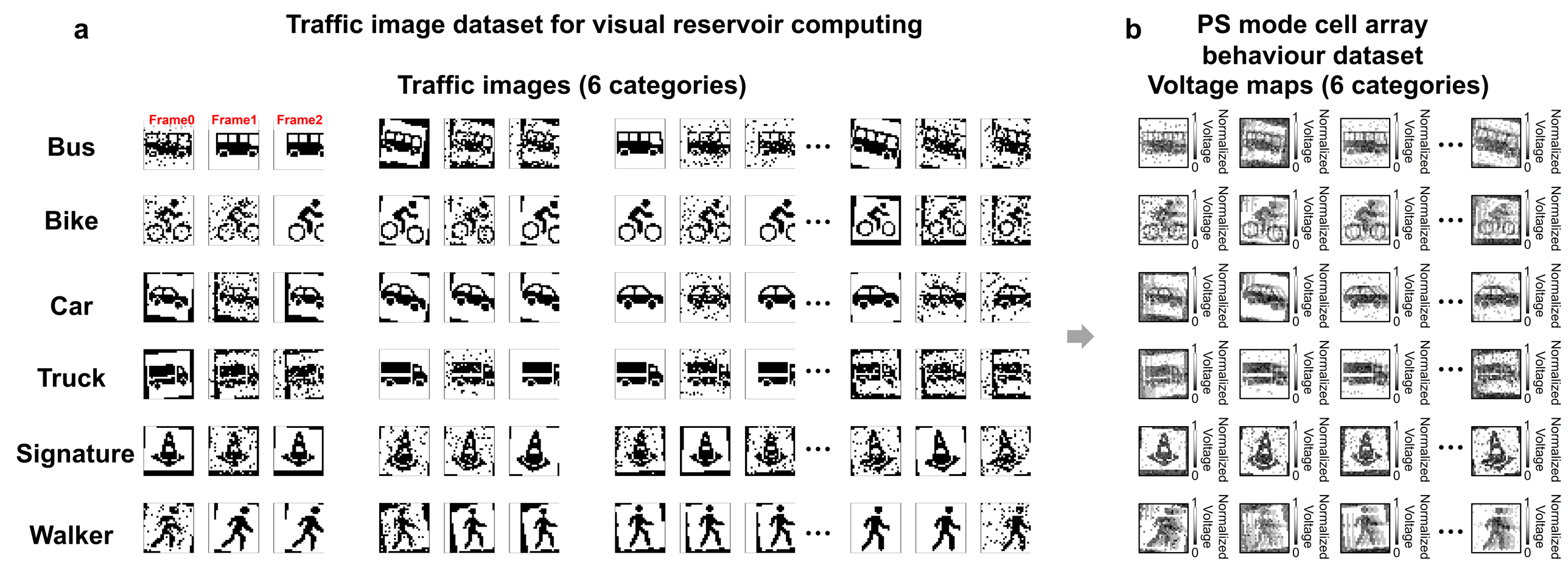


**Supplementary Fig. 28 | The traffic image dataset and the simulated PS mode behaviour dataset for visual reservoir computing.** (a) The traffic image dataset includes six categories (bus, bike, car, truck, sign, walker). Image noise includes perspective distortion (applied with 50% probability), random rotation (angle ∈ [–15°, 15°], applied with 50% probability), and salt-and-pepper noise (50% probability, amount = 0.2). (b) Simulated voltage maps generated from the PS mode cell array, with all device-to-device variation and cycle-to-cycle variation considered. These voltage maps serve as the PS-mode array behaviour dataset used to train the reservoir computing network in this configuration.


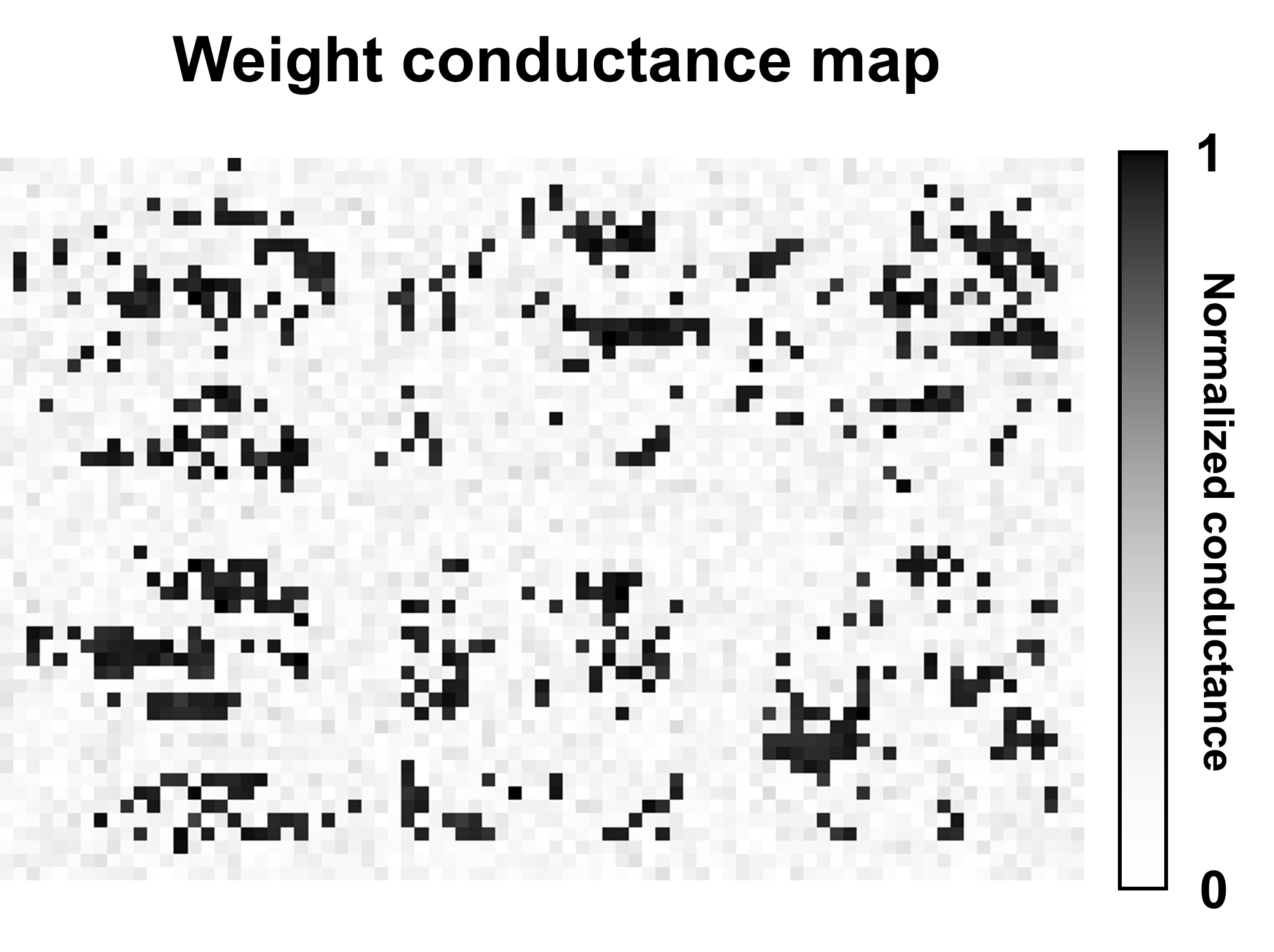


**Supplementary Fig. 29 | Weight map of visual reservoir computing output layer.** Cell variability was incorporated during simulation by adding Gaussian noise to the conductance values in the visual reservoir computing output layer, with the noise variance extracted from experimental measurements of the ES mode cell array, reflecting both cell-to-cell and cycle-to-cycle noise.


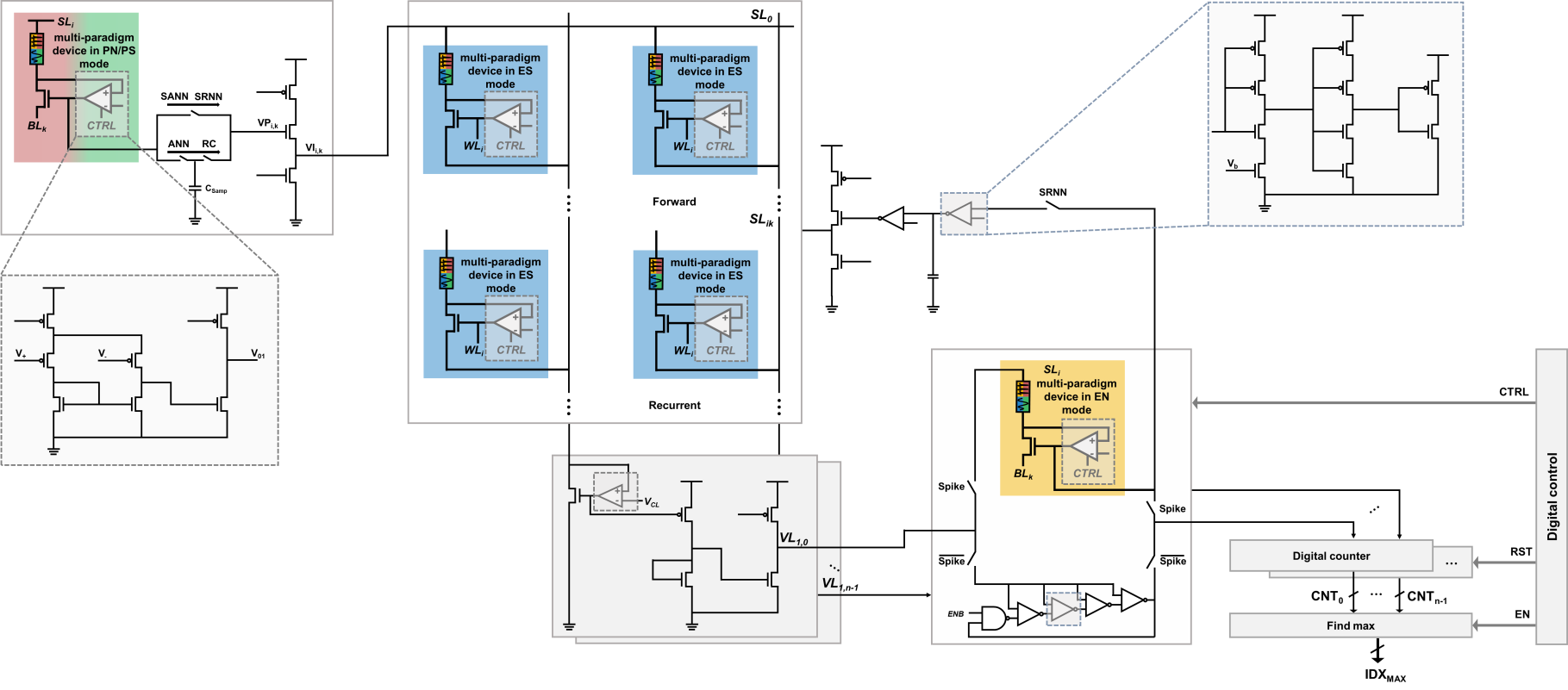


Supplementary Fig. 30 | The detailed system architecture of the reconfigurable vision system.

**Supplementary Note 1 - Mechanisms of the four sensory computing modes of multi-paradigm device**

**Photo-spiking neuron (PN) mode (0 V):** The photoresponsive LIF behaviour arises from the dynamic trapping and de-trapping of electrons at the ITO/CuO_x_ interface (Supplementary Fig. 4, top left). Upon illumination, photoexcited electrons are generated in CuO_x_, some of which are collected by the ITO electrode, producing a baseline photocurrent. At the same time, the remaining electrons occupy trap states at the ITO/CuO_x_ interface. As these interfacial trap states become populated, the barrier width and effective barrier height (the electron barrier seen from the CuO_x_ side to the ITO side) at the interface gradually decrease, corresponding to the integration phase. Once the barrier narrows sufficiently and reaches a tunnelling threshold, the trapped electrons are detrapped and released by the illumination and tunnel through the ITO/CuO_x_ interfacial barrier, resulting in a sharp spiking current (the firing event). Afterwards, the barrier at the ITO/CuOₓ interface naturally returns to its original width, completing a self-recovery cycle.

**Photo-synaptic (PS) mode (-0.1 to -0.04 V):** The observed photosynaptic response originates from the capture and release dynamics of photo-generated electrons within the trap states of CuO_x_ (Supplementary Fig. 4, top right). When exposed to light, electron–hole pairs are generated in the CuO_x_ film. These charge carriers are driven apart by a weak applied electric field, directing electrons toward the Pd electrode and holes toward the ITO electrode, thereby enhancing the photocurrent. Simultaneously, electrons within the trap states of the CuO_x_ are also excited and contribute to the photocurrent. As illumination continues, the current increase begins to slow down due to saturation of both photogeneration and de-trapping processes. Once the light is turned off, the free carriers rapidly recombine, while some excess electrons become re-trapped in CuO_x_ trap states, resulting in a non-linear decay of the current over time, characteristic of short-term synaptic plasticity.

**Electrical synaptic (ES) mode (0.1 to 0.5 V):** The analogue-type resistive switching originates from the change of the interfacial energy barrier, which is regulated by the movement of oxygen ions within the CuO_x_ layer (Supplementary Fig. 4, bottom left). In the initial state, the random distribution of oxygen ions results in moderate resistance. The set and reset operations are described in the following two stages, where 1) Set to low resistance state (LRS): when a small positive voltage is applied to the ITO electrode, oxygen ions migrate towards and into the ITO electrode. This results in a net hole doping effect, causing the Fermi level in the CuO_x_ layer to shift downwards, reducing the effective electron barrier height; 2) Reset to high resistance state (HRS): when a negative voltage is applied, the oxygen ions move back into the CuO_x_ layer eliminating the net hole doping effect, thus raising the effective electron barrier height.

**Electrical neuron (EN) mode (1.3 to 2 V):** The LIF behaviour in the electrical domain arises from the modulation of the interfacial energy barrier and the dynamic trapping of electrons within the CuO_x_ film (Supplementary Fig. 4, bottom right). Initially, the device resides in a high-resistance state (HRS). When a sequence of positive voltage pulses is applied to the ITO electrode, the barrier for electron injection from the Pd side is effectively lowered, allowing electrons to migrate into the CuO_x_ layer and drift toward the ITO electrode. During this process, a portion of the injected electrons is captured in trap states within CuO_x_, producing a net doping effect that enhances the CuO_x_ film conductivity. Meanwhile, oxygen ions move towards and into the ITO under the applied bias, reducing the effective barrier height. The continuous electron trapping and oxygen ion migration processes correspond to the leaky integration process. Upon cessation of the voltage pulses, the device enters a recovery phase.

**Supplementary Note** **2 - Calculation details of power for the cell**

Supplementary Table 1 shows the evaluation of power consumption for the cell in each operational mode. The detailed calculation is provided in Supplementary Table 2. For the cell-level evaluation, we consider the combined power consumption of the device and the mode-control circuit. Specifically, the power consumption of the mode-control circuit is 1.1 pW at a target bandwidth of 200 Hz, as obtained through circuit simulation using Cadence Virtuoso with a commercial 180 nm CMOS PDK.

**Supplementary Note 3 - Details of the visual spiking artificial neural network visual algorithm**

***Network Structure and Hardware Implementation:***

Supplementary Fig. 15 shows the details of the system in visual spiking artificial neural network configuration. The input sensory spiking neurons are implemented by the 20 × 20 PN mode cell array and 12 output neurons are implemented by the EN mode cell array. The synapses (400 × 12=4800) connecting the input and output neuron layers are implemented by the ES mode cell array. The PN mode cell array first encodes the continuous colour-mixed motion information into event-based voltage spiking signals and executes the colour-mixed motion trajectory extraction and filtering. Then, the voltage spikes are input into the ES mode cell array and EN mode cell array for AI-based high-level processing.

***Motion trajectory dataset:***

Examples of the scenarios in the motion trajectory dataset for visual spiking artificial neural network configuration are shown in Supplementary Fig. 16. In total, 12 classes of mixed-colour vehicle motion images are presented, consisting of target vehicles (car) and noisy vehicles (motorcycle) with 3 colours and 4 possible moving directions. The dataset contains 600 images (12 categories) with each car moving in a different direction across 20 sequential motion steps. Each image is 20 × 20 pixels in size. Similarly, the simulated PN mode cell array output includes 12 categories of spike map patterns, each with a resolution of 20 × 20 pixels and consisting of 20 sequential spikes. Among the 600 spike maps, 480 were used for training the AI section in this configuration, and 120 were reserved for testing the AI section of this configuration.

***Workflow of NI-based low- and mid-level processing scheme and AI-based high-level processing scheme:***

The PN mode cell array senses and processes the optical signals in the colour-mixed motion images $L$ (size 20 × 20) into voltage spiking signals, denoted as $U=\left( U_{1}\ldots U_{400} \right)_{t_{0}-t_{20}}$, representing the spiking activity of the PN mode cell array over the 20 motion steps ($t_{0}$ to $t_{20}$).  For each motion step ($t_{s}$ to $t_{s+1}$), the light intensities from the input image are encoded into a corresponding spike map $U_{t_{s}-t_{s+1}}$, which contains processed and extracted motion information.

$U$is then sent into ES mode cell array for AI-based MAC operations: ${U^{\mathbf{'}}= \left( U_{k}^{'} \right)}_{t_{0}-t_{20}}=\left( F_{MAC, k} \right)_{t_{0}-t_{20}}, k=1\ldots12$, where $F_{MAC, k}$ denotes the MAC operation for each column of ES mode cell array, while $U_{k}^{'}$ denotes the output voltage signals from each column of ES mode cell array. The index $k$ refers to the column number. Finally, $U^{\mathbf{'}}$ are sent to the EN mode cell array for threshold comparing and final decision making : $f_{out}=\left( {{\text{\{}f_{comparing}(U}_{m}^{'})\text{\}}}_{m=1}^{12} \right)_{t_{0}-t_{20}}$.The output neuron with the highest spike rate ($f_{out}$) during the interval from $t_{0}$ to $t_{20}$ indicates the final recognition result. There are 12 neurons in total, corresponding to 12 different classes. The corresponding weight maps are illustrated in Supplementary Fig. 17. The output voltage spike maps and spike trains from the ES mode and EN mode cell arrays, using a test image from the dataset's class 5, are presented in Supplementary Fig. 18.

**Supplementary Note 4 - Details of the visual spiking recurrent neural network visual algorithm**

***Network Structure and Hardware Implementation:***

Supplementary Fig. 19 shows details of the visual spiking recurrent neural network visual system. The input sensory spiking neurons are implemented by a 21 × 21 PN mode cell array to encode the motion data and filter out the static background, executing NI-based low- and mid-level processing. The recurrent layers consisting of 32 recurrent neurons, 441 × 32 forward recurrent synapses, and 32 × 32 backward recurrent synapses are implemented by the ES and EN mode cell arrays. The fully connected layers consisting of 8 output neurons and 256 (32 × 8) synapses are implemented with the EN mode and ES mode cell array for final prediction.

***Motion trajectory dataset:***

Examples of the scenarios in the motion trajectory dataset for visual spiking recurrent neural network are shown in Supplementary Fig. 20. The input continuous vehicle motion image contains both vehicle motion trajectory information and static background information. The motion trajectory dataset contains 1,640 distinct motion images with 8 possible moving directions. Similarly, the PN mode cell array behaviour dataset consists of 8 possible spike map patterns (21 × 21), each containing 10 sequential spikes. 1312 of these spike maps were used for training the AI section in this configuration, and the rest of the 328 spike maps were used for testing the performance of this configuration.

***Workflow of NI-based low- and mid-level processing scheme and AI-based high-level processing scheme:***

The PN mode cell array senses and processes the optical signals in the 21 × 21 input motion image ($L\text{ = }{\text{(}L_{1}\cdots L_{441}\text{)}}_{t_{0}-t_{10}}$) into voltage spiking signals ($\text{U =}{{(U}_{1}\cdots U_{441})}_{t_{0}-t_{10}}$). Each input motion image ($t_{0}$ to $t_{10}$) includes 10 continuous motion steps in total, with each motion step occupying a single period ($t_{s}$ to $t_{s+1}$). The optical signals of the input motion image within the period of one motion step (indexed as $s$) are represented as ${L_{t_{s}-t_{s+1}}=\text{(}L_{1}\cdots L_{441}\text{)}}_{\text{t}_{\text{s}}\text{-}\text{t}_{\text{s+1}}}$_._  $L_{t_{s}-t_{s+1}}$ are first sensed and pre-processed by the PN mode cell array into voltage spike signals. The output voltage spike signals of PN mode cell array are denoted as $U_{t_{s}-t_{s+1}}=\left( U_{1}\ldots U_{441} \right)_{t_{s}-t_{s+1}}$. $U_{t_{s}-t_{s+1}}$ is then input into the recurrent forwards-connection synapse (ES mode cell array) (441 × 32) for MAC operations, outputting voltage spiking signals ${U_{t_{s}-t_{s+1}}^{'}= \left( U_{k}^{'} \right)}_{t_{s}-t_{s+1}}, k=1\ldots32$. Meanwhile, the output voltage spike signals of the recurrent neurons array during the previous time step ($t_{s-1}-t_{s}$) are also input into the recurrent backwards-connection synapse array (32 × 32) for MAC operations, outputting voltage spike signals ${R_{t_{s}-t_{s+1}}=\left( R_{k} \right)}_{t_{s}-t_{s+1}}, k=1\ldots32$. The voltage spike signals $U_{t_{s}-t_{s+1}}^{'}$and $R_{t_{s}-t_{s+1}}$from the recurrent forwards-connection synapse array and recurrent backwards-connection synapse array are simultaneously input to the recurrent neurons array, outputting voltage spike signals ${U_{t_{s}-t_{s+1}}^{''}= \left( U_{k}^{''} \right)}_{t_{s}-t_{s+1}} ,k=1\ldots32$. $U_{t_{s}-t_{s+1}}^{''}$ is then input into the fully connected (FC) synapse array (32 × 8) for MAC operation, outputting voltage spiking signals ${U_{t_{s}-t_{s+1}}^{'''}= \left( U_{k}^{'''} \right)}_{t_{s}-t_{s+1}} ,k=1\ldots8$. Finally, $U_{t_{s}-t_{s+1}}^{'''}$ are input to the output neuron array (EN mode cell array). The output neuron that has the highest spike count ($f_{out}$) during $t_{0}$ to $t_{10}$ indicates to the predicted motion direction at $t_{10}-t_{11}$_._ The corresponding weight maps of the RFC, RBC, and FC synapse arrays are shown in Supplementary Fig. 21. The output voltage spike maps from the EN mode cell array are shown in Supplementary Fig. 22.

**Supplementary Note 5 - Details of the visual artificial neural network visual algorithm**

***Network Structure and Hardware Implementation:***

Supplementary Fig. 23 shows the details of the visual artificial neural network visual system fully implemented with the multi-paradigm device. The visual artificial neural network consists of an input sensory non-spiking neuron layer (784) and a synapse layer (784 × 8). The PS mode device cell array and ES mode device cell array implement the sensory non-spiking neuron layer and the synapse layer, respectively.

***Traffic sign image dataset:***

The datasets used in visual artificial neural network are shown in Supplementary Fig. 24. The traffic sign image dataset includes eight classes of 28 × 28 noisy traffic sign images: warning electricity, bump, danger, low-temperature freezing conditions, slippery road surface, harmful, CCTV in operation, and highly flammable. The image noise includes perspective distortion (applied with 50% probability), and salt-and-pepper noise (amount = 0.5). The simulated PS mode cell array behaviour dataset (28 × 28 voltage maps) is generated based on experimental measurements of the PS mode cell in the prototype. Both cycle-to-cycle and cell-to-cell variations are extracted and modelled as Gaussian noise, and added during the simulation to emulate realistic cell-level variation. The PS mode cell array behaviour dataset is used to train the AI section in this configuration.

In the PS mode cell array behaviour dataset, 192 samples are used for training, 39 for validation, and 48 for testing. Training is conducted using the Adam optimizer (learning rate = 0.001, β_1_ = 0.9, β_2_ = 0.999, and ε = 1×10^-8^) and CrossEntropyLoss. A maximum of 50 epochs is used, and early stopping is applied based on the validation loss, with a patience of 15 epochs. The final model is selected from the epoch that achieves the highest validation accuracy before any overfitting is observed. Training accuracy and training loss are also monitored to avoid underfitting.

***Workflow of NI-based low-level processing scheme and AI-based high-level processing scheme:***

The input noisy traffic sign images (28×28) ($L=\left( L_{1}\ldots L_{784} \right)$) are sensed and pre-processed by the PS mode cell array. The output voltage map from the PS mode cell array can be denoted as $U=\left( U_{1}\ldots U_{784} \right)$. $U$ is then input into the ES mode cell array for MAC operations: $y_{out}=\left( y_{k} \right)=F_{MAC,k}, k=1\ldots8$, where $F_{MAC,k}$ denotes the MAC operation performed by each column of ES mode cell array, and $y_{k}$ represents the output voltage amplitude from each column of the ES mode cell array. The output voltage signals from ES mode cell array are represented as $y_{out}$. The output voltage signal with the highest amplitude corresponds to the final recognized category of the input noisy traffic sign. Supplementary Fig. 25 depicts the weight maps stored in the ES mode cell array.

Supplementary Fig. 26 illustrates the comparison of classification accuracy between the non-spiking NI/AI visual artificial neural network configuration with NI-based pre-processing and an ANN visual system without pre-processing. The NI/AI visual artificial neural network achieves a classification accuracy of 97% in recognizing eight categories of traffic sign images, representing a 18% improvement over the traditional artificial neural network system.

**Supplementary Note 6 - Details of the visual reservoir computing visual algorithm**

***Network Structure and Hardware Implementation:***

Supplementary Fig. 27 shows the details of visual reservoir computing visual system. The visual reservoir computing configuration consists of a reservoir layer (729 = 27 × 27×3 / 3) and an output layer (729 × 6). The PS mode device cell array implements the reservoir layer and the ES mode device cell array implements the output layer.

***Traffic image dataset:***

Supplementary Fig. 28 presents the traffic image dataset for visual reservoir computing, which includes six categories of noisy traffic images—bus, bike, car, truck, sign, and walker. Image noise comprises perspective distortion, random rotation (angle ∈ [–15°, 15°]), and salt-and-pepper noise (amount = 0.2), each applied with 50% probability. The simulated voltage output of the PS mode cell array was used as the PS mode behaviour dataset for training the AI section in this configuration, featuring six categories of voltage map patterns. During simulation, both cell-to-cell and cycle-to-cycle noise during compression operation are incorporated as Gaussian noise extracted from small-scale measurements.

Of the total dataset, 480 images are used for training, 115 for validation, and 120 for testing. Training is conducted using the Adam optimizer (β_1_ = 0.9, β_2_ = 0.999, and ε = 1×10^-8^), a learning rate of 0.001, and the CrossEntropyLoss. Models are trained for a maximum of 30 epochs. Validation loss is monitored at each epoch, and early stopping is applied with a patience of 15 epochs to prevent overfitting. Training accuracy and training loss are also monitored to avoid underfitting.

***Workflow of NI-based low-level processing scheme and AI-based high-level processing scheme:***

The input images (27 × 27 × 3) ($L=\left( L_{1}\ldots L_{2187} \right)$) are sensed and compressed by the PS mode cell array. Each PS mode cell can sense and encode a light pulse sequence, consisting of 3 successive light pulses from the neighbouring 3 pixels, into a single analogue conductance state. The output voltage signal from PS mode cell array$\text{ }U=\left( U_{1}\ldots U_{729} \right)$ is sent into ES mode cell array for MAC operations:$y_{out}=\left( y_{k} \right)=F_{MAC,k}, k=1\ldots6$, where $F_{MAC,k}$ denotes the MAC operation performed by each column of ES mode cell array. The index $k$ denotes the column number. The output voltage signals from each column of ES mode cell array are represented as $y_{k}$, which correspond to the likelihood of the image belonging to one of the six possible classes. The column with the highest voltage signal determines the final classification result. Supplementary Fig. 29 depicts the weight maps stored in the ES mode cell array.

**Supplementary Note 7 – Calculation details of system power consumption and energy efficiency**

The evaluation of the power consumption and energy efficiency of the system across various configurations in Supplementary Table 6 is detailed in Supplementary Table 7-12.

For multiply and accumulate (MAC) operations in the ES mode cell array, OPS (Operations per Second) are calculated as: Operations per Second = (Size of the ES mode cell array × 2 × 2)×f, where f is the operating frequency in Hz. The first multiplication factor ×2 arises from the two distinct operations of multiplication and accumulation in each MAC operation performed by the ES mode cell array, while the second multiplication factor ×2 accounts for the use of two separate columns to represent signed weights, one for positive weights and the other for negative weights (Supplementary Fig. 11).

**Supplementary Note** **8** **– Area Efficiency Calculation**

For this work, area efficiency was estimated as:

$$\text{Area Efficiency (OPS/}F^{2})=\frac{\text{Opeartions per second }}{\text{Feature size (F²)}}$$

Feature size was obtained by scaling system area to the adopted technology node (180 nm). The Supplementary Table 13 shows the minimum and maximum values for area efficiency based on the system configurations.

**References**

1 Wu, G. *et al.* Ferroelectric-defined reconfigurable homojunctions for in-memory sensing and computing. *Nature Materials* **22**, 1499-1506 (2023). https://doi.org/10.1038/s41563-023-01676-0

2 Fu, Y. *et al.* Reconfigurable Synaptic and Neuronal Functions in a V/VOx/HfWOx/Pt Memristor for Nonpolar Spiking Convolutional Neural Network. *Advanced Functional Materials* **32**, 2111996 (2022). https://doi.org/https://doi.org/10.1002/adfm.202111996

3 Dang, B. *et al.* Reconfigurable in-sensor processing based on a multi-phototransistor–one-memristor array. *Nature Electronics* **7**, 991-1003 (2024). https://doi.org/10.1038/s41928-024-01280-3

4 Huang, H. *et al.* Fully integrated multi-mode optoelectronic memristor array for diversified in-sensor computing. *Nature Nanotechnology* **20**, 93-103 (2025). https://doi.org/10.1038/s41565-024-01794-z

5 Yang, Y. *et al.* Firing feature-driven neural circuits with scalable memristive neurons for robotic obstacle avoidance. *Nature Communications* **15**, 4318 (2024).

6 Zhu, J. *et al.* A heterogeneously integrated spiking neuron array for multimode‐fused perception and object classification. *Advanced Materials* **34**, 2200481 (2022).

7 Han, J.-K. *et al.* Cointegration of single-transistor neurons and synapses by nanoscale CMOS fabrication for highly scalable neuromorphic hardware. *Science Advances* **7**, eabg8836 (2021). https://doi.org/doi:10.1126/sciadv.abg8836

8 Wu, Q. *et al.* Spike Encoding with Optic Sensory Neurons Enable a Pulse Coupled Neural Network for Ultraviolet Image Segmentation. *Nano Letters* **20**, 8015-8023 (2020). https://doi.org/10.1021/acs.nanolett.0c02892

9 Han, J.-K. *et al.* in *2021 IEEE International Electron Devices Meeting (IEDM).* 1-4 (IEEE).

10 Wang, X. *et al.* Vertically integrated spiking cone photoreceptor arrays for color perception. *Nat Commun* **14**, 3444 (2023). https://doi.org/10.1038/s41467-023-39143-8

11 John, R. A. *et al.* Optogenetics inspired transition metal dichalcogenide neuristors for in-memory deep recurrent neural networks. *Nature Communications* **11**, 3211 (2020). https://doi.org/10.1038/s41467-020-16985-0

12 Yamazaki, T. *et al.* in *2017 IEEE International Solid-State Circuits Conference (ISSCC).* 82-83.

13 Yang, X. *et al.* A Bio-Inspired Spiking Vision Chip Based on SPAD Imaging and Direct Spike Computing for Versatile Edge Vision. *IEEE Journal of Solid-State Circuits* **59**, 1883-1898 (2024). https://doi.org/10.1109/JSSC.2023.3340018

14 Murakami, H. *et al.* in *2022 IEEE International Solid-State Circuits Conference (ISSCC).* 104-106.

15 Bose, L., Dudek, P., Carey, S. J. & Chen, J. in *2023 IEEE/CVF Conference on Computer Vision and Pattern Recognition Workshops (CVPRW).* 3995-3996.

16 Carey, S. J., Lopich, A., Barr, D. R. W., Wang, B. & Dudek, P. in *2013 Symposium on VLSI Circuits.* C182-C183.

17 Millet, L. *et al.* A 5500-frames/s 85-GOPS/W 3-D Stacked BSI Vision Chip Based on Parallel In-Focal-Plane Acquisition and Processing. *IEEE Journal of Solid-State Circuits* **54**, 1096-1105 (2019). https://doi.org/10.1109/JSSC.2018.2886325

18 Li, Z. *et al.* A reconfigurable heterostructure transistor array for monocular 3D parallax reconstruction. *Nature Electronics* **8**, 46-55 (2025). https://doi.org/10.1038/s41928-024-01261-6
